# Re-sequencing Expands Our Understanding of the Phenotypic Impact of Variants at GWAS Loci

**DOI:** 10.1371/journal.pgen.1004147

**Published:** 2014-01-30

**Authors:** Susan K. Service, Tanya M. Teslovich, Christian Fuchsberger, Vasily Ramensky, Pranav Yajnik, Daniel C. Koboldt, David E. Larson, Qunyuan Zhang, Ling Lin, Ryan Welch, Li Ding, Michael D. McLellan, Michele O'Laughlin, Catrina Fronick, Lucinda L. Fulton, Vincent Magrini, Amy Swift, Paul Elliott, Marjo-Riitta Jarvelin, Marika Kaakinen, Mark I. McCarthy, Leena Peltonen, Anneli Pouta, Lori L. Bonnycastle, Francis S. Collins, Narisu Narisu, Heather M. Stringham, Jaakko Tuomilehto, Samuli Ripatti, Robert S. Fulton, Chiara Sabatti, Richard K. Wilson, Michael Boehnke, Nelson B. Freimer

**Affiliations:** 1 Center for Neurobehavioral Genetics, Semel Institute for Neuroscience and Human Behavior, University of California Los Angeles, Los Angeles, California, United States of America; 2 Department of Biostatistics and Center for Statistical Genetics, University of Michigan, Ann Arbor, Michigan, United States of America; 3 The Genome Institute at Washington University, St. Louis, Missouri, United States of America; 4 Genome Technology Branch, National Human Genome Research Institute, Bethesda, Maryland, United States of America; 5 Department of Epidemiology and Biostatistics, MRC-PHE Centre for Environment and Health, School of Public Health, Imperial College London, London, United Kingdom; 6 Faculty of Medicine, St Mary's Campus, Imperial College London, London, United Kingdom; 7 Institute of Health Sciences, University of Oulu, Oulu, Finland; 8 Biocenter Oulu, University of Oulu, Oulu, Finland; 9 Unit of Primary Care, Oulu University Hospital, Oulu, Finland; 10 Department of Children and Young People and Families, National Institute for Health and Welfare, Oulu, Finland; 11 Oxford Centre for Diabetes, Endocrinology and Metabolism, University of Oxford, Churchill Hospital, Headington, Oxford, United Kingdom; 12 Wellcome Trust Centre for Human Genetics, University of Oxford, Oxford, United Kingdom; 13 Oxford NIHR Biomedical Research Centre, Churchill Hospital, Headington, Oxford, United Kingdom; 14 Institute for Molecular Medicine Finland, FIMM, University of Helsinki, Helsinki, Finland; 15 The Program for Human and Population Genetics, The Broad Institute of MIT and Harvard, Cambridge, Massachusetts, United States of America; 16 Institute of Clinical Medicine/Obstetrics and Gynecology, University of Oulu, Oulu, Finland; 17 Hjelt Institute, University of Helsinki, Helsinki, Finland; 18 Wellcome Trust Sanger Institute, Hinxton, United Kingdom; 19 Department of Health and Research Policy, Stanford University, Stanford, California, United States of America; Baylor College of Medicine, United States of America

## Abstract

Genome-wide association studies (GWAS) have identified >500 common variants associated with quantitative metabolic traits, but in aggregate such variants explain at most 20–30% of the heritable component of population variation in these traits. To further investigate the impact of genotypic variation on metabolic traits, we conducted re-sequencing studies in >6,000 members of a Finnish population cohort (The Northern Finland Birth Cohort of 1966 [NFBC]) and a type 2 diabetes case-control sample (The Finland-United States Investigation of NIDDM Genetics [FUSION] study). By sequencing the coding sequence and 5′ and 3′ untranslated regions of 78 genes at 17 GWAS loci associated with one or more of six metabolic traits (serum levels of fasting HDL-C, LDL-C, total cholesterol, triglycerides, plasma glucose, and insulin), and conducting both single-variant and gene-level association tests, we obtained a more complete understanding of phenotype-genotype associations at eight of these loci. At all eight of these loci, the identification of new associations provides significant evidence for multiple genetic signals to one or more phenotypes, and at two loci, in the genes *ABCA1* and *CETP*, we found significant gene-level evidence of association to non-synonymous variants with MAF<1%. Additionally, two potentially deleterious variants that demonstrated significant associations (rs138726309, a missense variant in *G6PC2*, and rs28933094, a missense variant in *LIPC*) were considerably more common in these Finnish samples than in European reference populations, supporting our prior hypothesis that deleterious variants could attain high frequencies in this isolated population, likely due to the effects of population bottlenecks. Our results highlight the value of large, well-phenotyped samples for rare-variant association analysis, and the challenge of evaluating the phenotypic impact of such variants.

## Author Summary

Abnormal serum levels of various metabolites, including measures relevant to cholesterol, other fats, and sugars, are known to be risk factors for cardiovascular disease and type 2 diabetes. Identification of the genes that play a role in generating such abnormalities could advance the development of new treatment and prevention strategies for these disorders. Investigations of common genetic variants carried out in large sets of research subjects have successfully pinpointed such genes within many regions of the human genome. However, these studies often have not led to the identification of the specific genetic variations affecting metabolic traits. To attempt to detect such causal variations, we sequenced genes in 17 genomic regions implicated in metabolic traits in >6,000 people from Finland. By conducting statistical analyses relating specific variations (individually and grouped by gene) to the measures for these metabolic traits observed in the study subjects, we added to our understanding of how genotypes affect these traits. Our findings support a long-held hypothesis that the unique history of the Finnish population provides important advantages for analyzing the relationship between genetic variations and biomedically important traits.

## Introduction

Genome-wide association studies (GWAS) based on common single nucleotide polymorphisms (SNPs) have unequivocally demonstrated the contribution of thousands of loci to risk for common diseases and to variation in quantitative traits. However for most such complex phenotypes, the variants identified to date appear to explain only a fraction of heritable variation, suggesting an important role for variants not assessed in GWAS. In particular, the hypothesis that currently unidentified low-frequency genetic variants may have a major impact on complex phenotypes has stimulated extensive efforts to discover such variants through next-generation sequencing.

Over the next several years it will increasingly become feasible to conduct comprehensive variant discovery through exome or whole genome re-sequencing studies. Such studies have the potential to demonstrate the impact on complex phenotypes of genes, pathways, and networks that GWAS have not yet implicated in these phenotypes. However it is increasingly clear that identifying associations at genome-wide or exome-wide thresholds of statistical significance will require large samples, and thus these experiments remain very costly. Although targeted re-sequencing studies of large samples do not provide the same likelihood of implicating novel genes as do genome-wide or exome-wide sequencing, they offer an excellent opportunity to obtain an initial picture of the relative phenotypic impact of variants across the complete allele frequency spectrum, in regions of interest. Such studies require evaluation of a relatively limited number of variants and, if prior evidence indicates that variants within the targeted region contribute to the phenotype, require a less stringent statistical threshold.

Genes within loci for which GWAS have shown significant associations represent logical foci for investigations across the allelic frequency spectrum. Several genes are now known to harbor both rare variants responsible for Mendelian disorders and common variants associated with related phenotypes [1], [2]. Resequencing of such genes may suggest particular variants as contributors to the GWAS signal, and may identify variants whose association with the phenotype is independent of the GWAS signal. Together, such variants provide starting points to investigate the heritable component of biological processes underlying the associated phenotypes.

We therefore undertook a re-sequencing study of Finnish cohorts, targeting loci identified from GWAS of quantitative metabolic traits, including: fasting blood levels of lipids and lipoproteins (triglycerides, TG; high-density lipoprotein cholesterol, HDL-C; low-density lipoprotein cholesterol, LDL-C; and total cholesterol, TC), glucose (FG), and insulin (FI). Several of these traits (TG, HDL-C, and FG) are components of the metabolic syndrome, an aggregation of variables that increase risk for type 2 diabetes (T2D) and cardiovascular diseases [3]. We report here the results of such targeted re-sequencing of >6,000 individuals drawn from a population cohort (the 1966 Northern Finland Birth Cohort, NFBC; [4]) and a T2D case-control sample (the Finland-United States Investigation of NIDDM Genetics study, FUSION; [5], [6], which included 919 individuals with T2D and 919 normal glucose-tolerant controls). In these individuals, we sequenced the coding regions of 78 genes selected from 17 loci that showed genome-wide significant association to one or more of the designated quantitative metabolic traits in GWAS meta-analyses that included these studies [7], [8]. Details on how we selected loci and genes within loci for re-sequencing can be found in Text S1.

We focused on these Finnish cohorts for two reasons, both of which concern the relationships expected between population history and the distribution of rare variants within a study sample. First, when a founder population has expanded recently from severe bottlenecks, as in Finland, many variants may disappear from the population while others increase rapidly in frequency owing to subsampling and genetic drift. Thus, while the overall number of rare variant sites observed in sequencing studies of the Finnish population is smaller than in other European populations [9], some deleterious variants are observed at a much higher frequency in Finland than in other populations. These variants include the mutations responsible for about 40 rare Mendelian disorders, the so-called “Finnish disease heritage” [10], [11]. We hypothesized that some variants with a large effect on quantitative metabolic phenotypes would also have attained a relatively high frequency in the Finnish population, so that by re-sequencing Finnish samples we could identify novel associations that might be unfeasible to detect in comparably sized samples from most other populations.

Second, the availability of information specifying the birthplace of most members of the NFBC and FUSION cohorts (or their parents) addresses the recently raised concern that unidentified population substructure may pose a particular issue in association analyses of rare variants (e.g. those with frequency <1%) [12]. This concern reflects the expectation that such variants have generally arisen more recently than common variants and are therefore more likely to differ in frequency between study populations; this concern is mainly relevant in studies where the geographical origin of the subjects is unknown [12]. Indeed, previous studies in Finnish samples (including NFBC) have shown that the available birthplace data provide a highly accurate delineation of population substructure [7], [10].

## Results

### Characteristics of study cohorts and re-sequencing summary statistics

Principal components analysis (PCA) using 122 k SNPs typed on genome-wide arrays revealed that the NFBC and FUSION samples overlap broadly in the first two PC dimensions (Figure S1). Phenotype distributions also overlap considerably between the cohorts (Table S1), and comparison of mean residual values after regressing the combined sample on age, age^2^, and sex showed no significant differences between NFBC and FUSION for any phenotype (p>0.77 for all comparisons; see Text S1), after excluding T2D cases from analysis of FG and FI.

We selected for re-sequencing the protein-coding regions and 5′ and 3′ untranslated regions (UTRs) of the genes within 17 loci that had previously demonstrated significant association (p<5×10^−8^) in GWAS to one or more metabolic phenotype (Table 1); TG (eight loci), HDL-C (nine loci), LDL-C (six loci), TC (nine loci), FG (six loci), and FI (one locus) [7], [8], [13]–[15]. The selection of the loci depended on the evidence from meta-analyses of several independent studies, but for eight of them, NFBC alone showed genome-wide significant association to one or more of the six phenotypes. We defined loci as the regions bracketed by the nearest recombination hotspots (>10 cM/Mb) on both sides of the reported GWAS SNPs. The numbers of genes included in the GWAS loci so defined ranged from one (four loci) to 50 (the *MADD* locus). As we did not have the resources to sequence all possible genes at each locus, we sequenced the genes nearest to the SNPs that showed genome-wide significant association with these phenotypes (see Text S1 for more detail), for a total of ∼270 kb of sequence.

**Table 1:** Overview of quantitative trait loci investigated in this study.

Locus^1^	Chr	5′ boundary (Build 37) (Mb)	Size (kb)	ROI^2^ (kb)	Genes Targeted/Total^3^	# Validated Variant Sites^4^	Associated Trait(s)^5^: Array SNP	Array SNP MAF^6^	Array SNP P-value^6^
*CELSR2*	1	109.656946	782.534	36.464	9/21	254	LDL-C:rs646776	.22	3.0E-17
							TC:rs646776		6.7E-13
*GALNT2*	1	230.273866	164.123	4.530	1/1	67	HDL-C:rs611229	.41	3.9E-05
							TG:rs4846930		2.3E-02
*GCKR*	2	26.893100	1594.61	2.189	1/42	12	FG:rs7588910	.33	2.8E-02
							TC:rs780090	.05	2.0E-03
							TG:rs1260326	.35	1.2E-12
*ABCG8*	2	43.458071	819.384	38.183	7/7	231	LDL-C:rs6756629	.09	9.8E-06
							TC:rs6756629		1.2E-05
*G6PC2*	2	169.312969	557.867	17.240	5/5	97	FG:rs560887	.31	1.1E-10
*LPL*	8	19.518908	458.35	3.747	1/3	43	HDL-C:rs10096633	.10	1.7E-06
							TG:rs10096633		5.4E-09
*ABCA1*	9	107.543376	201.285	11.176	1/1	73	HDL:rs2575875	.30	3.4E-05
							TC:rs2740486	.45	1.5E-03
*PANK1*	10	91.343009	62.133	3.684	1/3	12	FI:rs1075374	.21	3.2E-05
*CRY2*	11	45.706162	210.619	10.997	3/4	60	FG:rs2696935	.16	4.8E-04
*MADD*	11	46.273702	5320.50	47.317	15/50	325	FG:rs2696935	.16	4.8E-04
							HDL-C:rs7946766	.19	2.5E-06
*FADS1*	11	61.282504	446.559	9.273	3/10	43	FG:rs2072114	.29	5.6E-04
							HDL-C:rs509360	.34	2.3E-03
							LDL-C:rs174546	.43	1.0E-06
							TC:rs174546	.43	5.7E-05
							TG:rs6591657	.15	2.5E-03
*MTNR1B*	11	92.664875	62.999	1.662	1/1	15	FG:rs7121092	.42	3.1E-08
*APOA1*	11	116.462585	640.063	5.057	4/11	55	HDL-C:rs12805061	.27	1.2E-04
							LDL-C:rs11216267	.46	8.3E-08
							TC:rs11216267	.46	6.1E-07
							TG:rs12805061	.27	8.4E-07
*MVK*	12	109.641978	474.532	6.093	2/7	54	HDL-C:rs12314392	.41	3.0E-03
*LIPC*	15	58.541083	319.88	2.175	1/1	27	HDL-C:rs1532085	.44	1.0E-12
							TC:rs261336	.23	2.6E-03
							TG:rs261336	.23	3.4E-04
*CETP*	16	56.733941	383.464	16.686	5/5	148	HDL-C:rs3764261	.27	7.3E-38
							LDL-C:rs12445698	.19	1.7E-02
							TC:rs12445698	.19	1.4E-03
							TG:rs1561140	.47	2.1E-02
*NCAN*	19	19.250145	628.669	55.570	18/20	336	LDL-C:rs12610185	.06	2.3E-03
							TC:rs2228603	.07	3.0E-05
							TG:rs2304130	.06	1.3E-05

1 The loci are named according to the first gene in the region of interest, starting at the 5′ end of the region.

2 ROI = region of interest. Number of kb sequenced for the locus.

3 Total Genes is the number of genes in the locus; Targeted Genes is the number of genes investigated in this study.

4 Please see Table S3 for a complete listing of all variant sites, along with their MAF and annotation.

5 Phenotype abbreviations: TC = total cholesterol, LDL-C = low density lipoprotein cholesterol, HDL-C = high density lipoprotein cholesterol, TG = triglycerides, FG = Fasting Glucose, FI = Fasting Insulin. Previous association evidence for lipid traits is from Kathiresan *et al.* 2008, Willer *et al.* 2008 and Teslovich *et al.* 2010; for glucose Dupuis *et al.* 2010, and for insulin Sabatti *et al.* 2009. The “array SNP” is the GWAS array-genotyped SNP (not sequence variant) with the smallest p-value to indicated traits in this study and is not necessarily the same SNP highlighted in previous studies.

6 Array SNP MAF and p-values are taken from the current study.

We conducted targeted Illumina sequencing using 150 bp probes designed to capture primarily coding sequence, in whole-genome amplified (WGA) DNA from 6,958 individuals; 6,123 of these individuals (4,447 NFBC, 836 FUSION normal glucose tolerant controls, and 840 FUSION T2D cases) passed quality control procedures (Text S1). Mean depth of coverage (per bp per person) per gene ranged from 31×–285× (Table S2, Figure S2, and Text S1). On average, 96% of sequenced base pairs within a gene had genotype quality score ≥50 in ≥75% of subjects; some genes were covered at this level for as few as 60% of base pairs (Table S2). After this initial quality control process, we identified 2,221 variant sites, 1,779 (80%) with MAF<1%.

### Validation of rare variants

It is difficult to distinguish between low count variants and sequencing artifacts, and we reasoned that such artifacts might be increased in our study given that all DNAs had been whole-genome amplified (WGA). We therefore attempted to validate low count variants by PCR-amplification of the putative variant site in genomic DNA from variant carriers (or WGA DNA if genomic DNA was not available) and sequencing using a different platform (Roche 454 FLX). We sequenced all variants identified in ≤3 individuals in our sample and not reported in dbSNP version 135 (N = 1,104, Text S1), and considered validation for the sites as (1) their being variable and (2) the specific non-reference genotypes being correct as called.

Overall, we validated 89.5% of these 1,104 sites including 100% of the 91 sites with variants present three times and 271 of 273 (99.3%) corresponding non-reference genotypes; 205 of 207 (99.5%) of the 207 sites with variants present twice and 397 of 414 (95.9%) corresponding non-reference genotypes. Among singletons, we validated 691 of 806 (85.7%) non-reference genotypes; however, 336 of these validated only in WGA DNA (the only DNA source available for these samples). Conservatively, we excluded from further analyses these 336 WGA-only singleton sites, along with 104 singleton sites that were refuted (49 sites), not covered (20 sites), or found to be WGA artifacts (35 sites). Eleven additional singleton sites were found to be homozygous alternative when validated, bringing the number of retained singleton sites to 366 and the total number of retained sites (among the 1,104 for which validation was attempted) to 663.

After validation, we included a total of 1,780 variable sites for further analysis. The subsequently released dbSNP version 137 included 76 of our non-validated sites: our experiments had directly refuted four of these sites, we had not adequately covered five of them, and we had validated 67 sites only in WGA DNA. We re-included the 72 non-refuted sites, bringing the total number of validated polymorphic sites for subsequent analysis to 1,852 (Table S3).

To quantify the increase in rare variation information provided by sequencing compared with genotyping, we calculated the overlap between variants found in this study and those observed in a larger Finnish sample: 9,660 Finnish participants from the population-based Metabolic Syndrome in Men (METSIM) study [16] who were genotyped with the Illumina ExomeChip. The ExomeChip captured only 346 (19%) of the 1,852 polymorphic sites that we identified through sequencing.

### Characteristics of sequence variants

The majority of sequence variants (1,114, 60%) were in coding sequence (37% non-synonymous [NS] and 23% synonymous) while 738 (40%) were in introns or UTRs (Figure S3). PolyPhen2 [17] predicted 236 variants to have a deleterious impact: 213 missense “probably damaging” and 23 nonsense variants. Of these 236 variants, 21 (19 missense and two nonsense) were present in homozygous form in at least one individual. For all 21 of these variants, the phenotype distributions for rare-allele homozygotes overlapped with the phenotype distributions of the common-allele homozygotes (Figure S4), suggesting these variants are not sufficient to cause extreme phenotypes. A total of 1,410 of the 1,852 validated variants (76%) had MAF<1%, including 486 (26%) singleton and 217 (12%) doubleton variants (Figure S5). Nucleotide diversity, as estimated by Watterson's measure θ_W_ = 7.1×10^−4^ was larger than the pair wise heterozygosity estimator θ_π_ = 3.5×10^−4^, reflecting the abundance of singleton sites.

We observed less overall variation than that seen in earlier sequencing studies of individuals of European descent; one variant site in every 147 bp sequenced, as compared to every 21 bp [9], 57 bp [18] or 83 bp [19]. While the sample size in the study of Nelson *et al.* [9] was larger (12,514 European Americans) than that of our study, the sample sizes in Tennessen *et al.* [19] and Fu *et al.* [18] were smaller (1,351 and 4,298 European Americans, respectively; note that the samples sequenced in the latter two studies represented two different data releases from the same dataset). Nelson *et al.* observed that in the Finnish samples in their study, the number of variant sites per kb of sequence, was about one-third that of similar sized samples from southern Europe. Thus, while differences in sequencing coverage and in the number of sequencing artifacts could partially account for our observation of reduced numbers of variant sites compared to other studies, the results of Nelson *et al.* suggest that the Finnish population bottleneck may have played a larger role.

The reduced variation observed in our study compared to the three previous studies, primarily reflects numbers of rare variants. Nelson *et al.* report that 95% of their variant sites were rare (MAF<0.5%), with 74% seen in only one or two copies. Similarly, Tennessen *et al.* report that 72% of variant sites were seen in ≤3 copies. In our study, by contrast, 72% of variants were rare, 38% were seen in one or two copies, and 44% were seen in ≤3 copies.

By down-sampling our data [20] to match the sample sizes of Tennessen *et al.* and Fu *et al.*, and down sampling the data of Nelson *et al.* to match our sample size, we directly compared our site-frequency spectra (SFS) with those observed in these three studies. We caution against over-interpretation of these SFS, as they can be impacted by differences between studies in the choice of genes sequenced, variant ascertainment, and coverage. Nevertheless, in our sample, a substantially lower percentage of coding variants have MAF<1% than in any of the other three studies (Table S4). Conversely, in our sample we observe a higher proportion of so called “Goldilocks alleles”: variants with MAF 0.5–2%, a frequency sufficient for single-variant analyses of potentially large-effect variants [21]. For example, while Nelson *et al.* report that 1.1% of NS variants are Goldilocks alleles, we observe that 7.4% of NS variants fall in this frequency range.

While we observe fewer rare variants than these other sequencing studies, the proportion of NS variants among rare coding variants in our study (65%; 95% CI = 62%–68%) is similar to that seen in Nelson *et al.* (63%). The proportion of rare variants predicted to be functional is also roughly similar between our study and other studies. For example, Tennessen *et al.* report that almost 96% of SNVs predicted to be functional have MAF<0.5%, and state an odds ratio of 4.2 that such rare variants are functional compared to variants with MAF>0.5%. We find that 89% of SNVs predicted to be functional are rare, and estimate an odds ratio of 3 (95% CI = 1.98–4.52).

### Phenotype associations

A total of 39 unique locus-phenotype combinations represent the previously reported associations between the 17 re-sequenced loci and one or more of the six metabolic phenotypes: 32 associations for lipid measures, six for fasting glucose, and one for insulin (Table 1). To follow up these previous findings, we conducted association tests on the combined NFBC/FUSION data (see Methods). We conducted single-variant tests (regression of phenotype residuals on an additively coded genotype, see Methods) to assess association in each of the 39 locus-phenotype sets for all validated variants with MAF>0.1%; tests under alternative genetic models did not reveal any additional association evidence. Since multiple independent association signals may be present at a locus, we evaluated the relation of each newly associated variant to the “array SNP,” the SNP genotyped in the combined NFBC/FUSION sample with smallest p-value in this sample in single-SNP association tests (Table 1). We then conducted single-variant analyses conditional on the array SNP, by including the array SNP genotype as a covariate in the linear regression.

We used gene-level tests to evaluate the collective impact of non-synonymous (NS) variants with MAF<1% for each of the 62 genes that harbored at least two such validated variants, considering only phenotypes that showed prior evidence of association to the locus (a total of 147 tests). We adopted this MAF threshold after determining that any higher MAF threshold simply recapitulated associations identified by the single-variant tests. Given different alternative models of interest, we performed two minimally correlated tests: CMC [22] which assumes the direction of effect for all rare variants is the same, and SKAT [23] which is better tuned to the setting in which the direction of effect of rare variants is mixed.

Taking the combined results from our single-variant and gene-level analyses, we evaluated to what degree re-sequencing of these 17 loci has advanced our understanding, beyond what was known from GWAS, of the phenotypic impact of genetic variation. We considered such an advancement to consist of either identification of additional, independent association signals, or the detection of association to rare variants.

For several of the lipid-associated loci, we were able to assess the evidence for multiple independent signals in relation to a similar analysis conducted on SNP data by Teslovich *et al.* 2010 [8]. This comparison has two limitations: our study and that of Teslovich *et al.* did not examine the same set of variants, and for five of the 13 lipid loci, our variant set did not contain a good proxy (r^2^>0.8) for the lead SNP of Teslovich *et al.* To counter these limitations, we used information imputed from NFBC data on pairwise LD between variants analyzed in the two studies, and assumed that any pair of variants with r^2^<0.2 in NFBC were effectively independent.

We used here a significance threshold of p<0.001 (approximately the cutoff obtained by applying the Benjamini-Hochberg [24] rule to control FDR at the 0.02 level across all the variants/genes and phenotypes tested, see Methods). For 27 of the 39 locus-phenotype combinations, the re-sequencing analysis essentially recapitulated the results from the GWAS. For the remaining 12 locus-phenotype combinations (at seven loci), we summarize below how re-sequencing has advanced our understanding of genotype-phenotype relationships; MAF, p-values, and annotations for all associated variants at these seven loci are presented in Table 2.

**Table 2:** Loci with multiple independent single-variant association signals to SNPs with MAF>0.1%.

Trait/Locus^1^	Gene	Variant	MAF	Allele	Type	Beta^2^	P-value^3^	PV array SNP only^4^	PV array SNP+sequence variants^5^
**LDL-C in ** ***ABCG8***		rs6756629	.090	A	array SNP	−0.15	1.3E-05		
	*ABCG8*	rs145756111	.011	A	synonymous	0.31	6.1E-04	1.2%	1.4%
**FG in ** ***G6PC2***		rs560887	.310	A	array SNP	−0.15	9.0E-12		
	*G6PC2*	rs138726309	.014	T	missense probably damaging	−0.41	2.6E-06	1.2%	1.7%
**HDL-C in ** ***LPL***		rs10096633	.100	A	array SNP	0.15	6.8E-06		
	*LPL*	rs268	.018	G	missense benign	−0.38	2.2E-07	0.72%	1.2%
**TG in ** ***LPL***		rs10096633	.100	A	array SNP	−0.19	1.9E-08		
	*LPL*	rs268	.018	G	missense benign	0.31	9.2E-05	0.59%	0.85%
**HDL-C in ** ***ABCA1***		rs2575875	.300	A	array SNP	−0.08	8.4E-05		
	*ABCA1*	rs2066718	.015	T	missense benign	0.32	9.3E-05		
	*ABCA1*	rs2066715	.055	T	missense benign	0.15	5.1E-04	0.62%	1.1%
**TG in ** ***APOA1***		rs12805061	.270	G	array SNP	0.004	.86		
	*APOA5*	rs2266788	.089	G	3′-UTR	0.27	7.7E-13		
	*APOA5*	rs3135506	.059	C	coding-synonymous	0.24	6.9E-08	0.4%	1.6%
**LDL-C in ** ***APOA1***		rs11216267	.460	G	array SNP	0.08	2.3E-05		
	*APOA5*	rs651821	.085	C	5′-UTR	0.13	2.2E-04	1.4%	1.6%
**TC in ** ***APOA1***		rs11216267	.460	G	array SNP	0.08	1.3E-04		
	*APOA5*	rs651821	.085	C	5′-UTR	0.14	9.3E-05	1.1%	1.4%
**HDL-C in ** ***LIPC***		rs1532085	.440	A	array SNP	0.13	9.4E-12		
	*LIPC*	rs28933094	.016	T	missense probably damaging	0.42	7.0E-08	1.2%	1.8%
**TG in ** ***LIPC***		rs261336	.230	G	array SNP	0.09	1.4E-04		
	*LIPC*	rs28933094	.016	T	missense probably damaging	0.33	2.7E-05	0.2%	0.5%
**HDL-C in ** ***CETP***		rs3764261	.270	A	array SNP	0.27	1.6E-40		
	*CETP*	rs5880	.024	C	missense probably damaging	−0.26	1.8E-05	3.3%	3.6%

1 FG and TG were LN transformed prior to analysis; the regression coefficient is on the LN scale.

2 Beta is the estimate of the regression coefficient, and provides the amount and direction the phenotype changes for each copy of the indicated allele.

3 The p-values come from separate multivariate models for each locus that include all variants listed below, and the first five PCs. P-values shown here represent the independent evidence for the specified variant, after conditioning on the array SNP.

4 The percent variance in the phenotype accounted for by just the array SNP.

5 The percent variance in the phenotype accounted for by the array SNP and the independent sequence variants.

#### *ABCG8* locus (LDL-C)

In re-sequencing seven genes at this locus, we identified 231 validated variants, and detected association independent of the array SNP with variant rs145756111 in the *ABCG8* gene (Figure S6AB, Table 2, p = 6.1×10^−4^). Comparison with the data of Teslovich *et al.* indicates three distinct LDL-C signals at this locus: (1) a single signal given by our array SNP and the Teslovich *et al.* lead SNP (r^2^ = 0.98); (2) the second common signal identified by Teslovich *et al.* (rs4953023); and (3) the rare variant signal identified here (rs145756111), which is independent of both common signals.

#### *G6PC2* locus (FG)

< Resequencing five genes at this locus revealed 97 validated variants and identified an association signal independent of the array SNP (Figure S6CD, Table 2, p = 2.6×10^−6^). The independent associated SNP rs138726309 codes for a missense variant (His177Tyr) predicted to be “probably damaging” by PolyPhen2 [17], occurring at a highly conserved site; the reference amino acid is observed at 25 of 26 aligned homologous proteins, refSeq NP_066999, SwissProt Q9NQR9. This SNP is a candidate causal variant and a priority for follow-up investigations; it is also significantly increased in frequency in Finland compared to other European populations (MAF = 0.014 in Finland vs MAF = 0.0023 in the European ancestry samples in the Exome Variant Server, http://evs.gs.washington.edu/EVS/, p<10−16).

#### *LPL* locus (HDL-C, TG)

 We re-sequenced only *LPL* at this locus, detecting 43 validated variants, including a nonsense variant, rs328, in strong LD with the array SNP. We identified a second variant, rs268, in low LD (r^2^ = 0.002) with rs328 and associated with both HDL-C and TG (Figure S6E–H, Table 2, HDL-C p = 2.2×10^−7^, TG p = 9.3×10^−5^). Comparison with the data of Teslovich *et al.* indicates three distinct signals at this locus for both HDL-C and TG: (1) a signal given by our array SNP and the Teslovich *et al.* lead SNP (r^2^ = 0.87); (2) the second signal reported by Teslovich *et al.* at rs7016529; and (3) the rare variant identified by sequencing, rs268.

#### *ABCA1* locus (HDL-C, TC)

We identified 73 validated variants in *ABCA1*, the only gene we sequenced in this region. Two sequence variants with single SNP associations to HDL-C are independent of the array SNP at this locus (Figure S6IJ, Table 2, variants rs2066718, p = 9.3×10^−5^, and rs2066715, p = 5.1×10^−4^, pair wise r^2^ = 0.000). Comparison with the data of Teslovich *et al.* suggests four HDL-C signals at this locus; our array SNP is modestly correlated with their lead SNP (r^2^ = 0.62), and poorly correlated with their independent signal at SNP rs11789603 (r^2^ = 0.17), and sequence variants rs2066718 and rs2066715 are not correlated to either of the common SNPs (r^2^<0.01 for all pair wise comparisons).

**Figure 1:**
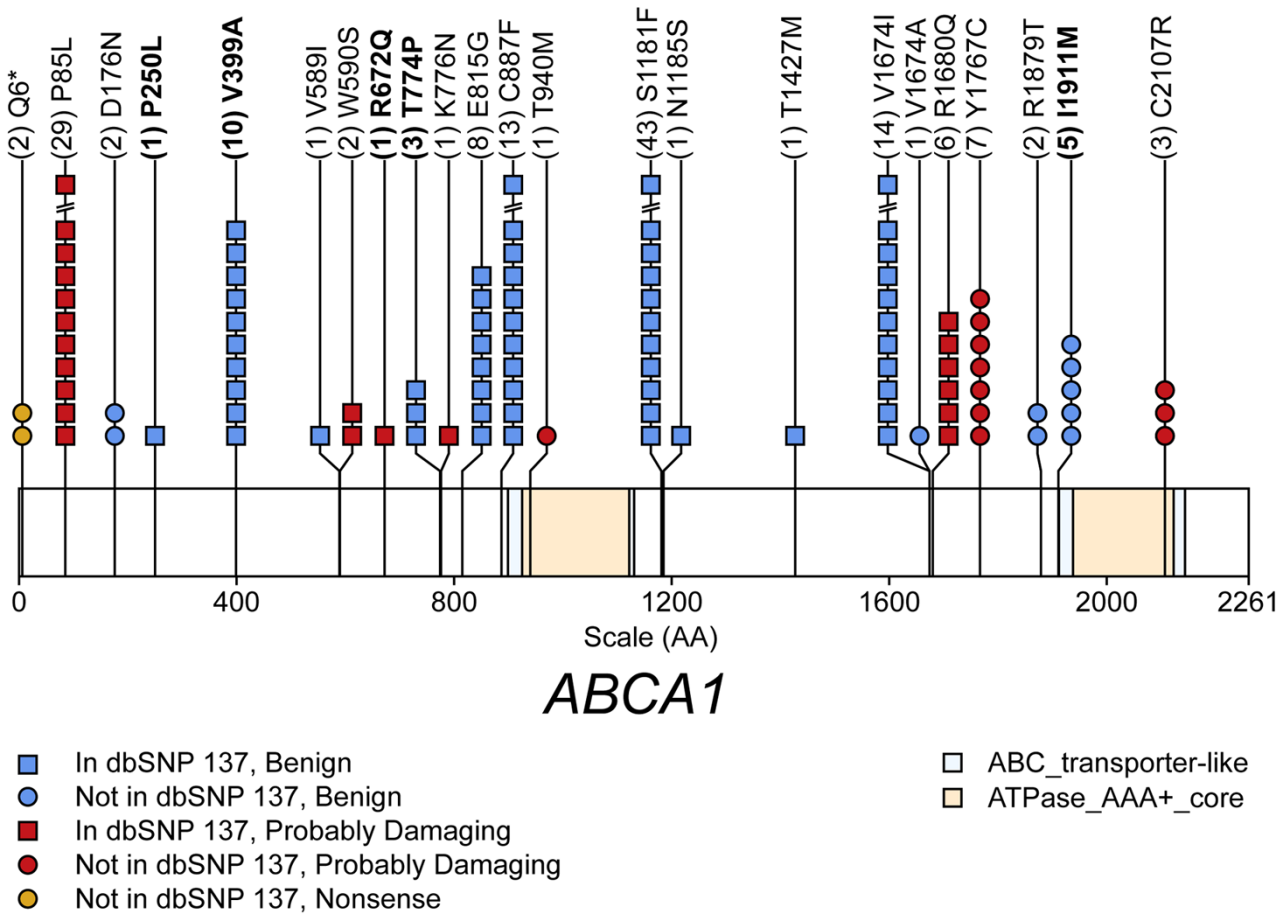
Schematic of rare (MAF<1%) non-synonymous variants used in the gene-level test of total cholesterol (TC) in gene *ABCA1*. The x-axis scale (AA) is in amino acid positions. Numbers in parenthesis are the number of copies of the rare variant in persons with phenotype data. The mean TC level in persons possessing variants with bold naming is increased relative to persons without the variant, for all other variants the mean TC level in persons possessing variant alleles is decreased relative to persons without the variant.

The gene-level tests used 23 *ABCA1* variants, eight not previously reported (as of dbSNP 137), one nonsense variant and seven missense variants, all predicted to be “probably damaging” (Table S3). These tests implicated rare *ABCA1* variants in both TC and HDL-C (TC: CMC p = 3.7×10^−5^, HDL-C: CMC p = 5.0×10^−4^). For TC, 18 of the 23 variant sites (with 138 of the 157 minor alleles) are associated with lower TC (Figure 1); 16 of these 23 variants were also associated with decreased HDL-C, based on single-marker tests. The observation that most variants have the same direction of effect is consistent with stronger association evidence for CMC than for SKAT (TC: SKAT p = 0.018, HDL-C: SKAT p = 0.0033; Table S5).

We attempted to determine whether any single rare variant could be responsible for the gene-level test signal at this locus, and, if so, whether its contribution could be separated from that of the more common variants assessed in the single-variant tests. For this purpose we used all of the non-singleton sequence variants detected in *ABCA1* to construct a multivariate linear model with HDL-C as a response variable. We employed stepwise regression analysis, using the Bayesian Information Criteria (BIC) criterion to select the best model (see Methods). That model (Table 3) includes six variants: the array SNP, the two independent common variants identified in the single-variant analyses (rs2066718 and rs2066715), and three rare variants (MAF = 0.00025, 0.00049, 0.00015), one of which (chr9:107548661) is predicted by PolyPhen2 to be a “probably deleterious” missense variant. Inclusion of all sequence variants along with the array SNP in a model to predict HDL-C increases the HDL-C variance explained to 1.8%, compared to 0.62% when HDL-C is modeled by the array SNP alone. Our results underscore the distinct contributions to HDL-C variation of both common and rare variants at this locus.

**Table 3:** Rare and common variants contribute to the association signal to HDL-C in gene *ABCA1*.

Variant	MAF	MAC	Allele	Type	Beta	P-value^1^
rs2575875	.30	4209	A	array SNP	−0.08	2.7E-04
rs2066718	.015	181	T	missense benign	0.32	1.0E-04
rs2066715	.055	674	T	missense benign	0.15	5.1E-04
Chr9:107548661	.00025	3	G	missense probably damaging	−2.08	2.9E-04
Chr9:107555091	.00049	6	C	missense benign	1.50	6.9E-04
Chr9:107555452	.00016	2	G	missense benign	−2.94	2.8E-05

1 P-values for rs2575875, rs2066718, and rs2066715 may be different from those recorded in Table 2 because of the addition of the three rare variants to the model. MAC: minor allele count.

#### *APOA1* locus (HDL-C, LDL-C, TC, TG)

We re-sequenced four genes in this region, discovering 55 variants, and gained additional understanding of the impact of this locus on LDL-C, TC, and TG. For TG, variants rs3135506 (p = 6.9×10^−8^) and rs2266788 (p = 7.7×10^−13^) demonstrate associations that are independent of each other, although neither is clearly independent of the array SNP (Figure S6KL, Table 2). For LDL-C and TC, variant rs651821 also shows association independent of the array SNP (Figure S6M–P, Table 2, p = 2.2×10^−4^ and p = 9.3×10^−5^, respectively). Comparison with Teslovich *et al.* suggests three distinct TC signals at this locus; while rs651821 shows modest correlation with their lead SNP (r^2^ = 0.56), our array SNP displays little correlation with two independent signals that they identified (r^2^≤0.1).

#### *LIPC* locus (HDL-C, TG, TC)

We identified 27 variants in re-sequencing *LIPC*, the only gene in this region, and detected association, independent of the array SNP, to HDL-C and TG (Figure S6Q–T, Table 2, p = 7.0×10^−8^ and 2.7×10^−5^, respectively). This independent signal is from rs28933094, a missense variant predicted to be “probably damaging,” that in recessive form causes hepatic lipase deficiency [25]. This variant is found at a higher frequency in Finland (MAF = 0.015) than in other European populations (MAF = 0.0019 among the European Ancestry samples in the Exome Variant Server, p<10^−16^). Comparison with Teslovich *et al.* suggests three independent associations for both HDL-C and TG at this locus. For HDL-C, our array SNP, rs1532085, is the lead SNP reported by Teslovich *et al.* and shows low LD (r^2^ = 0.004) with variant rs28933094 or with an independent signal reported by Teslovich *et al.* (rs2070895). For TG, our array SNP, rs261336, is modestly correlated with the independent signal (rs261334) reported by Teslovich *et al.* (r^2^ = 0.57); however, our variant rs28933094 is not correlated (r^2^ = 0.004) with Teslovich *et al.* lead SNP, rs1532085.

#### *CETP* locus (HDL-C, LDL-C, TG, TC)

We re-sequenced five genes in this region, identifying 148 variants, and detected HDL-C association that is independent of the array SNP to variant rs5880 (Figure S6UV, Table 2, p = 3.9×10^−5^). Comparison with the data of Teslovich *et al.* suggests three distinct HDL-C associations at this locus; our array SNP, rs3764261, is identical to their lead SNP; however, they identified an independent signal at SNP rs9939224 that is not well correlated with rs5880 (r^2^ = 0.07).

**Figure 2:**
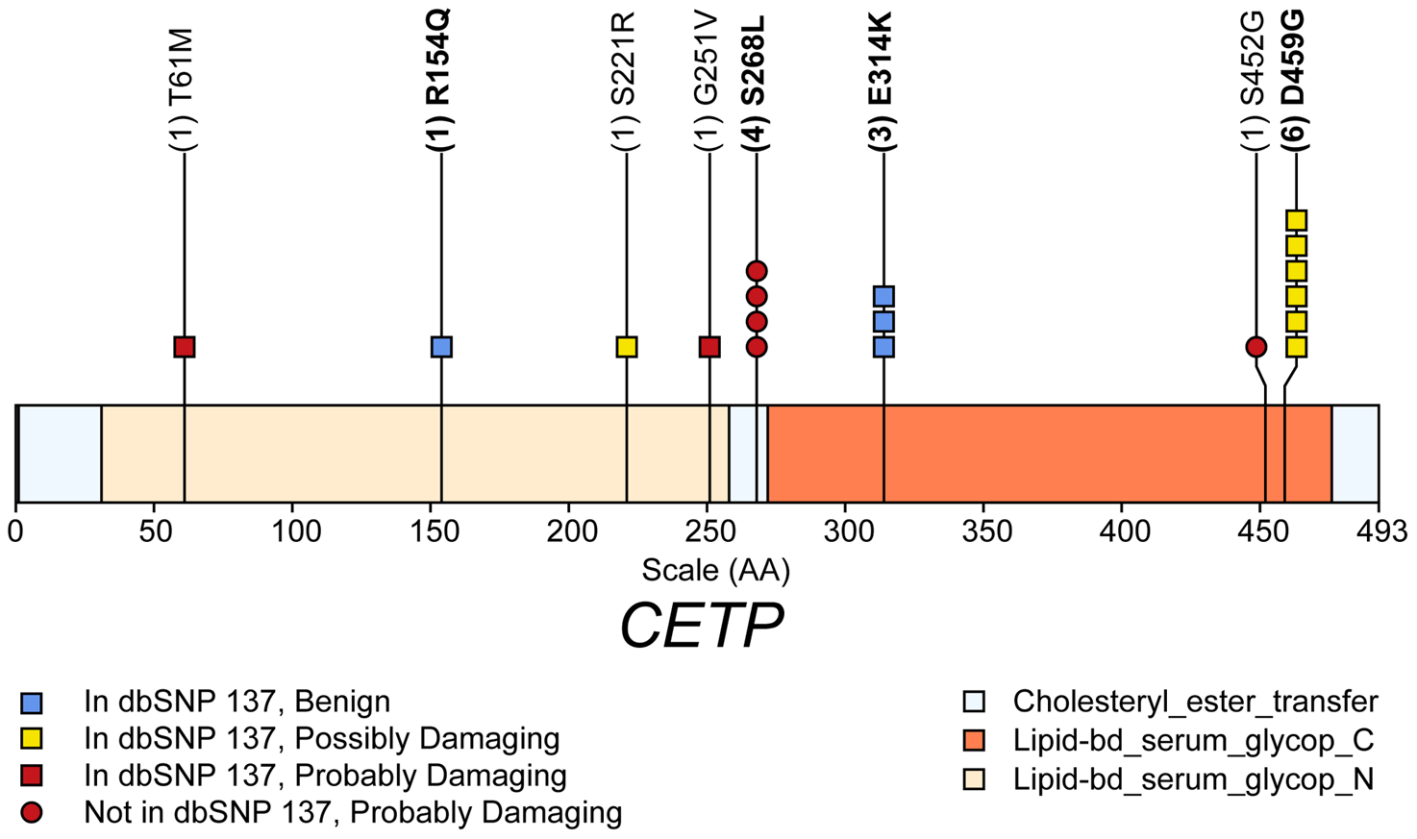
Schematic of rare (MAF<1%) non-synonymous variants used in the gene-level test HDL-C in gene *CETP*. The x-axis scale (AA) is in amino acid positions. Numbers in parenthesis are the number of copies of the rare variant in persons with phenotype data. The mean HDL-C level in persons possessing variants with bold naming is increased relative to persons without the variant, for all other variants the mean HDL-C level in persons possessing variant alleles is decreased relative to persons without the variant.

Gene-level analyses at this locus used 65 variants in 5 genes and highlighted the contribution to HDL-C of rare variants in *CETP*, including four missense variants predicted to be “probably damaging”, two of which were not in dbSNP 137 (Table S3) (SKAT p = 6.4×10^−4^, Table S5, Figure 2). The fact that four of the eight NS variants in *CETP* were associated with increased HDL-C and four with decreased HDL-C explains the stronger association evidence with SKAT than with CMC. For other phenotypes, it is not clear that re-sequencing substantially advanced our understanding of the role of this locus. A multivariate linear model (Table 4) for *CETP* selects four variants for HDL-C response: the array SNP, two common variants (rs5880 and rs5883), and a rare variant (rs2303790, a missense variant predicted by PolyPhen2 to be “probably deleterious”). The array SNP alone accounts for 3.3% of variance in HDL-C; by adding the sequence variants to the model the proportion of HDL-C variability explained increases to 4.1%, underscoring the distinct contributions to HDL-C variation made by both common and rare variants in this gene.

**Table 4:** Rare and common variants contribute to the association signal to HDL-C in gene *CETP*.

Variant	MAF	MAC	Allele	Type	Beta	P-value^1^
rs3764261	.27	3913	A	array SNP	0.28	1.6E-40
rs5883	.040	490	T	coding-synonymous	0.22	5.2E-06
rs5880	.024	296	C	missense probably damaging	−0.29	4.2E-05
rs2303790	.00057	7	G	missense possibly damaging	1.31	1.0E-03

1 P-values for rs3764261 and rs5880 may be different from those recorded in Table 2 because of the addition of rs2303790 and rs5883 to the model. MAC: minor allele count.

We also carried out a T2D case-control analysis by comparing FUSION T2D cases to the combination of NFBC participants and FUSION controls. The first five PCs were included as covariates to control for stratification. Single-variant analyses conducted for the 442 SNPs with MAF>1% revealed no significant associations to T2D, using either the standard definition of genome-wide significance (p<5×10^−8^) or the less stringent Bonferroni threshold of .05/442 = 1.1×10^−4^.

## Discussion

Large-scale re-sequencing has the potential to identify a comprehensive set of variants that are missed by imputation and chip based fine-mapping approaches. In more than 6,000 members of Finnish cohorts assessed for metabolic traits, we re-sequenced 78 genes implicated in prior GWAS of these traits, identifying 1,852 total variants, including >200 predicted-deleterious missense variants and 23 nonsense variants, 125 of which are not currently in the public database (dbSNP 137). Using single-variant analyses, we found associations at seven loci (six involving one or more variants with MAF<5%, Table 2) and demonstrated using conditional analyses that these signals are independent of previously reported GWAS SNPs. Using gene-level tests we found compelling association evidence for rare variants in two genes, *ABCA1* and *CETP*. By comparison, Hunt *et al.* [26] in a large (>40,00 individuals) autoimmune disease case-control sample, found that targeted coding region re-sequencing of 25 GWAS risk genes provided minimal new information. Several differences between our studies could account for the apparent discrepancies in findings: First, the genetic architecture of quantitative metabolic traits may be simpler than that of the diseases investigated by Hunt *et al.* Second, we benefitted from the effect of Finnish population history, which has led to a larger proportion of variants in the Goldilocks allele range and a smaller proportion of rare variants (about 70% of the variants observed by Hunt *et al.* are present in one or two copies, compared to <40% in our study). Third, the genes for which we identify rare variant associations may be unusual in their tolerance for functional variation.

Our gene-level test results for *ABCA1* agree with two previous lines of evidence that rare variants in this gene could have an impact on lipid phenotypes. First, recessive mutations in *ABCA1* cause extreme reduction in HDL-C, termed Tangier Disease or hypoalphalipoproteinemia; several of these variants were discovered in Finnish families [27]. Second, previous studies in diverse populations found enrichment of NS *ABCA1* variants in individuals with low HDL-C levels [21], [28]. Among the fifteen previously described rare NS variants observed in our data, ten have previously been implicated in metabolic phenotypes: Tangier Disease (n = 3), increased risk for heart disease (n = 2), or either reduced (n = 3) or elevated (n = 2) serum HDL-C levels (Human Gene Mutation Database).

Our results have enabled us to clarify genotype-phenotype relationships for eight of the 17 loci examined. By delineating multiple distinct association signals, and in some instances highlighting specific candidate alleles, they also suggest potential targets for functional investigations that could specify causal variants. For example, at *G6PC2* we identified a Goldilocks allele at rs13872630 which has a predicted deleterious effect. This variant has a distinct signal from the array SNP, and appears to have a much stronger effect in lowering FG. As this effect may provide protection against cardiovascular disease [29], there may be great value in generating mice mutated for this His177Tyr missense variant, which occurs at a highly conserved site. Additionally, the relatively high frequency of this variant within Finland offers an unusual opportunity to evaluate its impact on a much wider range of phenotypes than we investigated here.

At the same time these findings also point to the difficulty in predicting the phenotypic impact of individual variants. Recessive mutations in several of the genes that we re-sequenced are causative for rare metabolic disorders (e.g. [27]). However the relatively modest effect on quantitative metabolic phenotypes that we observed for variants in these and other genes predicted to be deleterious (nonsense and missense) suggest two possibilities: 1) the genetic and/or environmental backgrounds in families demonstrating Mendelian metabolic disorders may differ from the backgrounds in individuals drawn for population samples, and 2) we must be cautious in assigning likely causality to variants on the basis of annotation alone.

The incomplete coverage obtained for several loci provides an additional reason for caution in our conclusions. Methods for capturing a targeted region have become more efficient since we completed our study, and therefore it is possible that implementation of such methods would provide more complete coverage at these loci and could identify additional novel variants with a large contributions to metabolic phenotypes.

Our prior hypothesis was that the process of genetic drift within a recently expanded founder population such as Finland should elevate the frequency of some deleterious alleles so that, even if they are subject to strong selective pressure, they may be observed at relatively high frequency [11]. In such populations, these variants may be sufficiently common for phenotype-associations to be detected using single-variant tests. As predicted by this hypothesis, our re-sequencing identified, in *G6PC2* and *LIPC*, two missense variants predicted to be deleterious that are very rare outside Finland (MAF<0.002), but that were sufficiently increased in frequency (MAF>0.013) in our study sample for us to detect significant association in single-variant tests. A recent genome-wide survey of copy number variations has similarly demonstrated that a rare deletion, highly over-represented within Finland, is associated with neurodevelopmental disorders [30]. Taken together, these results suggest that exome-wide and genome-wide investigations of Finnish population cohorts will likely identify additional associations to complex phenotypes that may not be apparent in other populations.

## Methods

### Study samples

We obtained genomic DNA samples processed at the Finnish Institute of Molecular Medicine (NFBC) and US National Human Genome Research Institute (FUSION). All NFBC and FUSION participants included in this study provided informed consent. The studies were carried out in accordance with the approvals of the Ethical Committee of the Northern Ostrobothnia Hospital District (for NFBC), and the University of Michigan Health Sciences and Behavioral Sciences Institutional Review Board (IRB-HSBS) and the Institutional Review Board of the National Public Health Institute (KTL; now part of the National Institute for Health and Welfare, THL) (for FUSION).

### Capture, sequencing, and quality control

We constructed Illumina multiplexed libraries with 5 µg of whole genome amplified material (see Text S1 for description of amplification procedures) or 1 µg native genomic DNA according to the manufacturer's protocol (Illumina Inc, San Diego, CA) with the following modifications: 1) DNA was fragmented using a Covaris E220 DNA Sonicator (Covaris, Inc. Woburn, MA) to between 100 and 400 bp. 2) Illumina adapter-ligated library fragments were amplified in four 50 µL PCR reactions for eighteen cycles. 3) Solid Phase Reversible Immobilization bead cleanup was used for enzymatic purification throughout the library construction process and for final library size selection targeting 300–500 bp fragments. Samples were multiplexed using Illumina barcoded libraries pooled together in pools of 12 or 18 depending on the sequencing platform. We designed a custom targeted set of 150 bp probes (Agilent Technologies, Santa Clara, CA) and captured ∼270 kb of primarily coding sequence from 78 genes. The concentration of each captured library pool was determined through qPCR according to the manufacturer's protocol (Kapa Biosystems, Inc, Woburn, MA) to produce cluster counts appropriate for the Illumina GAIIx and HiSeq 2000 platforms.

Sample pools of 12 and 18 were loaded on GAIIx and HiSeq machines, respectively, using paired end 101 bp read lengths. We aimed to achieve a coverage metric of 80% of the targeted space covered at ≥20× depth. We aligned reads from each sample to the NCBI37/hg19 reference sequence using BWA [31]. Sample identity was confirmed by comparing sequence data (SAMtools consensus calls) with pre-existing genotype array data. Individuals with ≥70% coverage at 20× and ≥90% genotype concordance with 51 array SNPs were included in the analysis (6,123 of 6,958 individuals).

### Generation of consensus variant data set

Details on sequencing and generation of center-specific genotype call sets can be found in Text S1. To generate a consensus call set, we pooled together all quality controlled sites discovered by any of the three centers (UCLA, University of Michigan, or Washington University) in the defined target loci (number of markers m = 2,306). We excluded multi-allelic sites or sites with different alternative alleles (m = 72). Each center then re-called SNP genotypes at the remaining sites (m = 2,234). Majority vote was used to generate variant calls. Genotypes concordant between at least two centers were included in the consensus data set; others were set to missing. The overall concordance rate between centers was 99.96% (99.99%, 99.94%, and 99.95% for homozygous reference, heterozygous, and homozygous alternative genotypes, respectively).

### Principal components analysis (PCA)

NFBC individuals were previously genotyped on the Illumina 370duo Chip, and all FUSION cases and 774 of 919 FUSION controls on the Illumina HumanHap300 BeadChip (version 1.0). After standard quality control procedures [6], [7], high-quality GWAS genotypes were available for 296,978 SNPs for all genotyped individuals. We used PLINK [32] to identify 122,644 SNPs with no more than moderate pair wise linkage disequilibrium (r^2^<0.5) which we used to calculate genetic principal components (PCs) with EIGENSTRAT [33].

### Association analysis

#### Phenotype transformation

We applied logarithm transformations to BMI, WHR, TG, glucose, insulin, and SBP to reduce skewness. For each phenotype (or its logarithm), data from the two studies combined were regressed on age, age^2^, and indicator variables for sex, oral contraceptive use, pregnancy status, and cohort, and residuals from this regression used in association analyses. T2D cases were excluded from analysis of FG and FI. Analyses were repeated using inverse normal transformed variables, and our conclusions were robust to choice of transformation.

#### Single-variant analysis

We tested variants with minor allele frequency (MAF)>0.1% for association with phenotype residuals in the combined NFBC/FUSION data set assuming an additive genetic model and including the first five PCs as covariates using PLINK [32]. We used conditional analyses to determine if single-SNP associations were independent of genotypes at the array SNP. Teslovich *et al.*, in a previous GWAS meta-analysis of lipid traits, identified, at several loci, associations independent of their GWAS signals [8]. We sought to evaluate, at all such GWAS loci highlighted by Teslovich *et al.*, the correlation between the signals identified through our conditional analyses and the “independent” signals detected by Teslovich *et al.* We could not make a direct comparison because at most loci the two studies did not have data on the same sets of variants. We instead used imputed data from Finnish reference populations to evaluate pairwise LD between the variants from our study that we compared with the variants from Teslovich *et al.* In this comparison we considered any pair of variants with r^2^<0.20 to be independent.

#### Gene-level tests

We conducted gene-level tests for phenotype residuals for each gene using non-synonymous variants with MAF<1%. Only individuals with complete genotype data for all variant sites were used in a given gene-level test of each gene; sample size for gene-level tests ranged from 4,651 to 5,376 individuals. There were 62 genes with >1 non-synonymous variant site with MAF<1%; the number of such sites ranged from 2 to 33 per gene.

We used the Combined Multivariate and Collapsing (CMC, [22]) test that uses a weighted-sum-score-based linear model to test the collective effect of multiple rare variants within a gene; see Text S1 for more information on the form of the weighting method used. We also employed the Sequence Kernel Association Test (SKAT, [23]), which assumes that the effect sizes for individual variants follow an arbitrary distribution with zero mean and an unknown variance. SKAT uses a score-based variance component approach to test the null hypothesis that the effect size distribution has zero variance. For both CMC and SKAT, we used the first five PCs as covariates.

For SKAT, we used asymptotic theory p-values, which conformed well to p-values estimated by permutation (data not shown). For the CMC, we estimated p-values based on 10,000 permutations of the phenotype data. To estimate the p-value for the *ABCA1* association, we performed 1,000,000 permutations.

#### Significance thresholds

We employed FDR controlling procedures [24] over the entire set of single-variant and gene-level tests we conducted. Testing of each phenotype against variants/genes in loci to which the phenotype had prior association resulted in 2,096 tests (1,802 single-variant tests and 294 gene-level tests). FDR control at the 0.05 level resulted in a p-value cut-off of 0.004, and we opted to use a somewhat more stringent p-value threshold of 0.001 corresponding to a FDR of 0.02.

#### Multivariate linear model selection

We used a standard model selection approach to analyze the 39 locus-phenotype combinations and derive the “best” multivariate linear model for each phenotype based on all variants at the locus. We excluded singletons from this analysis because the absence of any replication in the study sample renders inference on their effect impossible without making strong parametric assumptions. As a default model selection approach, we used the Bayesian Information Criterion (BIC) using a greedy search built into R (StepAIC). We again included only complete observations in each locus-phenotype combination. For each locus, the model with the smallest number of predictor variables included the first five PCs, and the model with the largest number of predictor variables included all non-singleton variants genotyped.

## Supporting Information

Figure S1 PC 1 and PC 2 from an analysis of GWAS data in FUSION (red) and NFBC (black) samples. FUSION: circles are individuals born in Lapland, crosses are individuals born in Oulu, triangles are individuals born elsewhere in Finland. NFBC: the birthplace of both parents of NFBC subjects are indicated by different symbols, in the legend the slash separates the location of birth of each parent. Lap = Lapland.
(PDF)

Figure S2 Summary of coverage by person and gene. A: Person-specific average depth of coverage over all the targeted genes. 107 persons with mean coverage >500× were omitted to improve plot clarity. B: Gene-specific average coverage depth across all subjects and all targeted basepairs within each gene. C: Relationship between the percent of target basepairs in a gene and GC content. HighGC = mean GC in a gene >60%; LowGC = mean GC in a gene < = 60%.
(PDF)

Figure S3 Distribution of variant types and targeted sequence regions. Top: proportion of variant sites (left) and sequenced basepairs (right) that are intronic, utr, coding (synonymous, missense and nonsense). The purple and yellow hatched region indicates coding basepairs. Bottom: proportion of variant site types by minor allele frequency category.
(PDF)

Figure S4 Boxplots of raw phenotypic values vs. the number of alternative alleles at deleterious variant sites. Deleterious sites are nonsense and missense variants predicted to be probably deleterious by PolyPhen-2. In the title, the number in parentheses is the number of persons homozygous for the alternative allele at the variant site.
(PDF)

Figure S5 Summary of variant allele frequency. A: Site frequency spectrum. On the y axis is the proportion of variant sites with a specified minor allele count. On the x axis are minor allele counts running from 1 (singleton sites) to 20. Data were down-sampled to a common sample size of 6,000 persons using the hypergeometic distribution. B: Relationship between minor allele frequency (MAF) and presence of variant in dbSNP 137.
(PDF)

Figure S6 Regions and phenotypes where significant association with single SNP analysis of SNPs with MAF>0.11% was independent of the array SNP association. For each phenotype-region combination two plots are presented, single-SNP association, and single-SNP association conditional on the array SNP (in purple, and labeled with text). Color scale is LD relative to the array SNP. On the y-axis is the −log10(P-value) for association to the indicated phenotype, on the x-axis is position in Mb from hg19. Up triangles: nonsense variants; down triangles: missense variants; squares: synonymous and utr variants; circles: no annotation available. The loci are named according to the first gene in the region of interest, starting at the 5′ end of the region. *A*. LDL-C in *ABCG8* locus *B*. LDL-C in *ABCG8* locus, conditional analysis *C*. FG in *G6PC2* locus *D*. FG in *G6PC2* locus, conditional analysis *E*. HDL-C in *LPL* locus *F*. HDL-C in *LPL* locus, conditional analysis *G*. TG in *LPL* locus *H*. TG in *LPL* locus, conditional analysis *I*. HDL-C in *ABCA1* locus *J*. HDL-C in *ABCA1* locus, conditional analysis *K*. TG in *APOA1* locus *L*. TG in *APOA1* locus, conditional analysis *M*. LDL-C in *APOA1* locus *N*. LDL-C in *APOA1* locus, conditional analysis *O*. TC in *APOA1* locus *P*. TC in *APOA1* locus, conditional analysis *Q*. TG in *LIPC* locus *R*. TG in *LIPC* locus, conditional analysis *S*. HDL-C in *LIPC* locus *T*. HDL-C in *LIPC* locus, conditional analysis *U*. HDL-C in *CETP* locus *V*. HDL-C in *CETP* locus, conditional analysis.
(PDF)

Table S1 Comparison of phenotypic values for NFBC participants and FUSION cases and controls.
(XLS)

Table S2 Description of the 78 genes sequenced.
(XLS)

Table S3 All 1,852 variants analyzed in the study. SNPs not found in dbSNP137 are named by the chromosome and position. Phenotype abbreviations are as in Table S1.
(XLS)

Table S4 Percent of variants in coding regions with MAF<1% in various studies. All comparisons involve persons of European ancestry.
(DOCX)

Table S5 Results of all gene-level tests of rare variants in genes that are found in regions of a priori association to the indicated phenotype.
(XLS)

Text S1 Supplementary methods.
(DOC)
