# Supplementary figures and images for: Re-sequencing Expands Our Understanding of the Phenotypic Impact of Variants at GWAS Loci
Source: PLoS Genet. 2014 Jan 30;10(1):e1004147. doi: 10.1371/journal.pgen.1004147 (PMC3907339; doi:10.1371/journal.pgen.1004147)

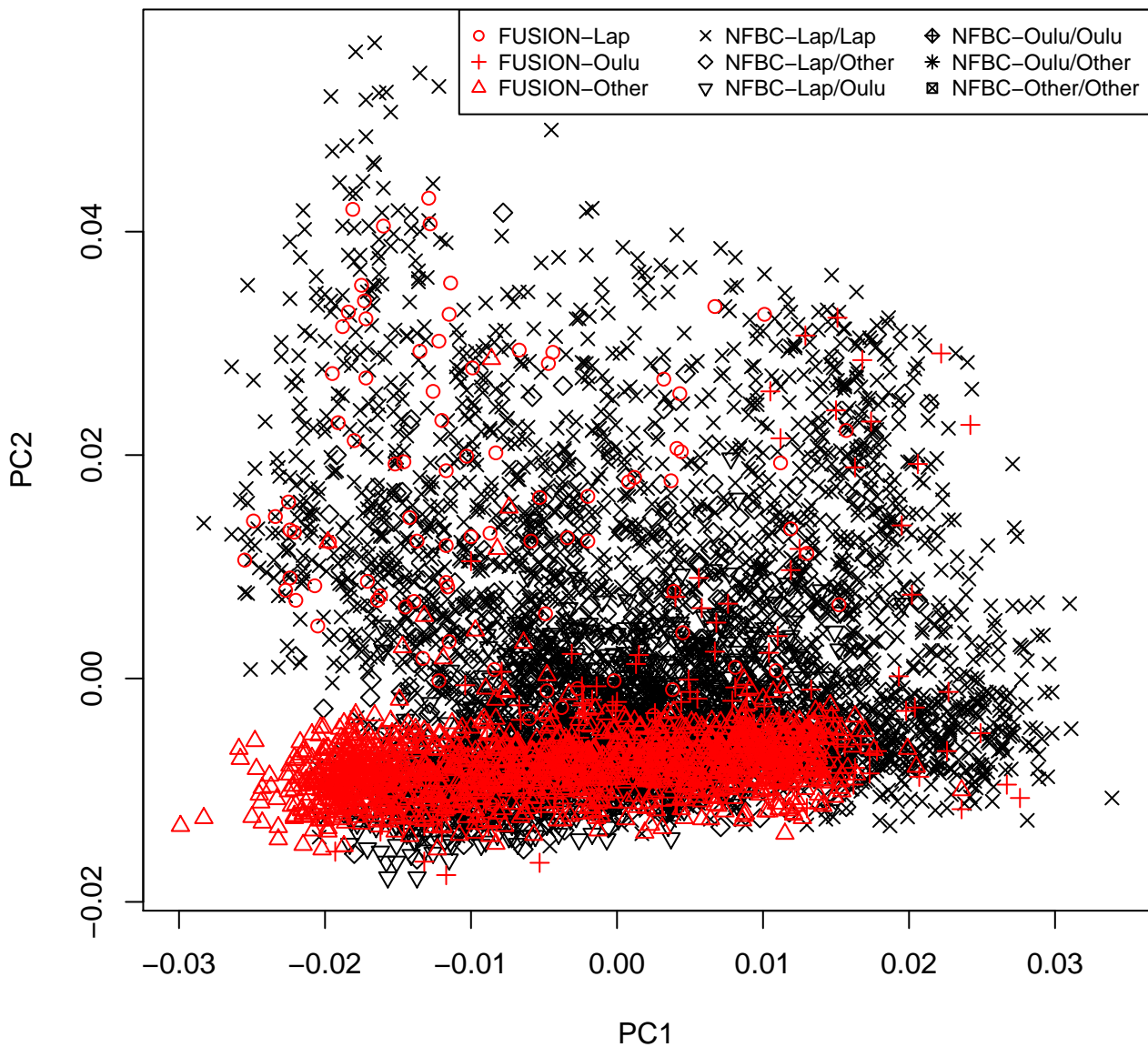

Supplement: Figure S1 — PC 1 and PC 2 from an analysis of GWAS data in FUSION (red) and NFBC (black) samples. FUSION: circles are individuals born in Lapland, crosses are individuals born in Oulu, triangles are individuals born elsewhere in Finland. NFBC: the birthplace of both parents of NFBC subjects are indicated by different symbols, in the legend the slash separates the location of birth of each parent. Lap = Lapland. (PDF) [file pgen.1004147.s001.pdf]

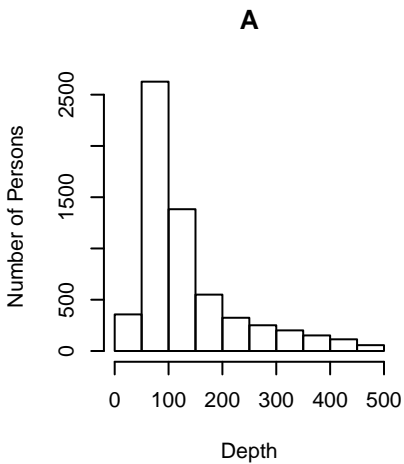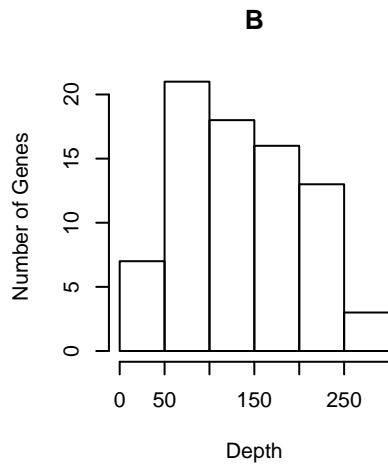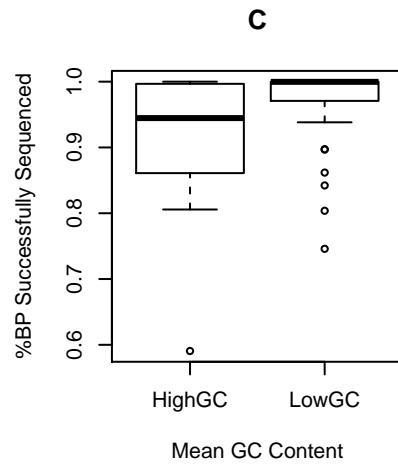

Supplement: Figure S2 — Summary of coverage by person and gene. A: Person-specific average depth of coverage over all the targeted genes. 107 persons with mean coverage >500× were omitted to improve plot clarity. B: Gene-specific average coverage depth across all subjects and all targeted basepairs within each gene. C: Relationship between the percent of target basepairs in a gene and GC content. HighGC = mean GC in a gene >60%; LowGC = mean GC in a gene < = 60%. (PDF) [file pgen.1004147.s002.pdf]

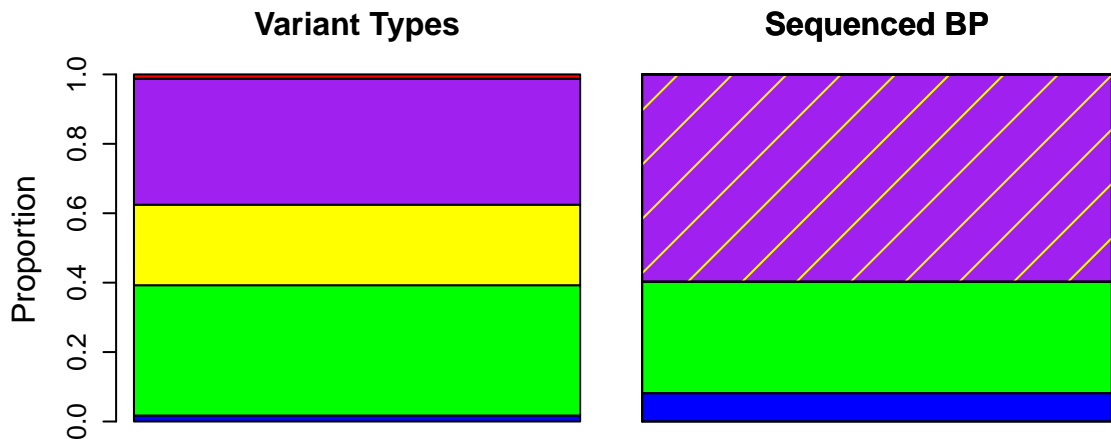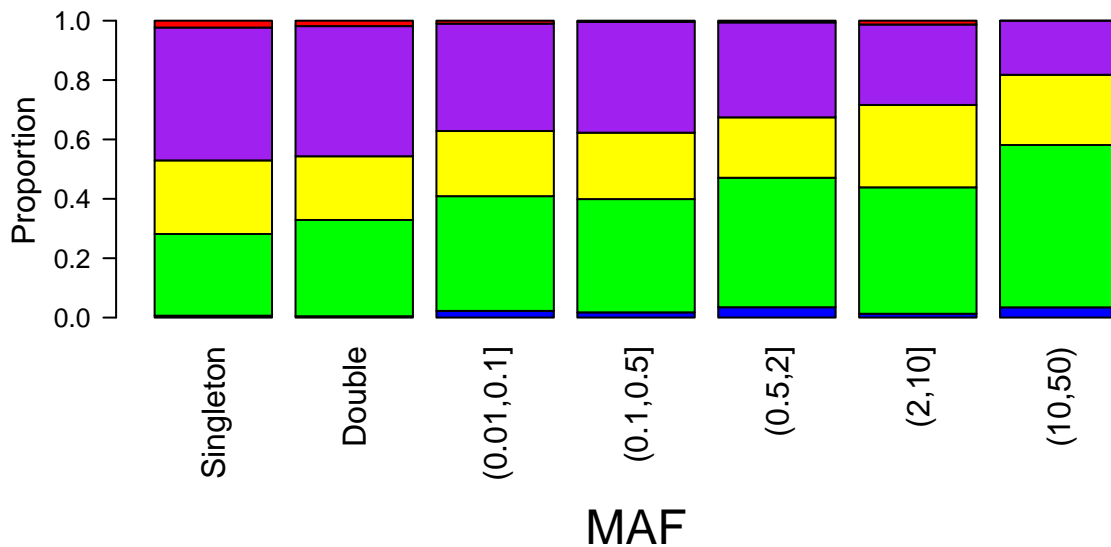

intron utr syn missense nonsense

Supplement: Figure S3 — Distribution of variant types and targeted sequence regions. Top: proportion of variant sites (left) and sequenced basepairs (right) that are intronic, utr, coding (synonymous, missense and nonsense). The purple and yellow hatched region indicates coding basepairs. Bottom: proportion of variant site types by minor allele frequency category. (PDF) [file pgen.1004147.s003.pdf]

**A**

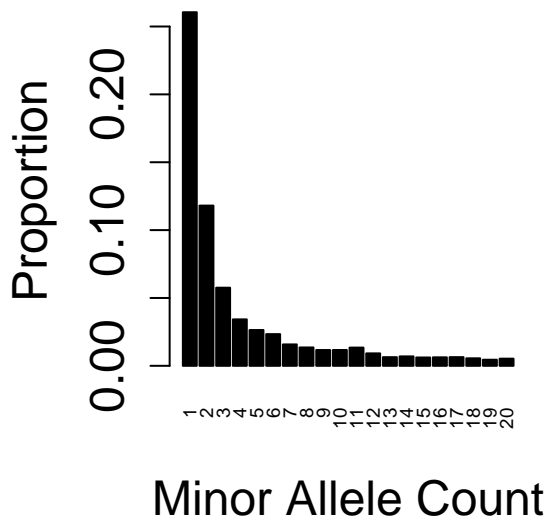

**B**

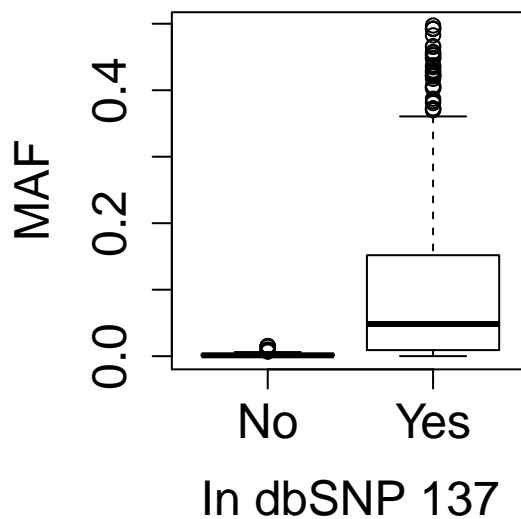

Supplement: Figure S5 — Summary of variant allele frequency. A: Site frequency spectrum. On the y axis is the proportion of variant sites with a specified minor allele count. On the x axis are minor allele counts running from 1 (singleton sites) to 20. Data were down-sampled to a common sample size of 6,000 persons using the hypergeometic distribution. B: Relationship between minor allele frequency (MAF) and presence of variant in dbSNP 137. (PDF) [file pgen.1004147.s005.pdf]
