# Supplementary material for: Re-sequencing Expands Our Understanding of the Phenotypic Impact of Variants at GWAS Loci
Source: PLoS Genet. 2014 Jan 30;10(1):e1004147. doi: 10.1371/journal.pgen.1004147 (PMC3907339; doi:10.1371/journal.pgen.1004147)
Supplement: Figure S4 — Boxplots of raw phenotypic values vs. the number of alternative alleles at deleterious variant sites. Deleterious sites are nonsense and missense variants predicted to be probably deleterious by PolyPhen-2. In the title, the number in parentheses is the number of persons homozygous for the alternative allele at the variant site. (PDF) [file pgen.1004147.s004.pdf]

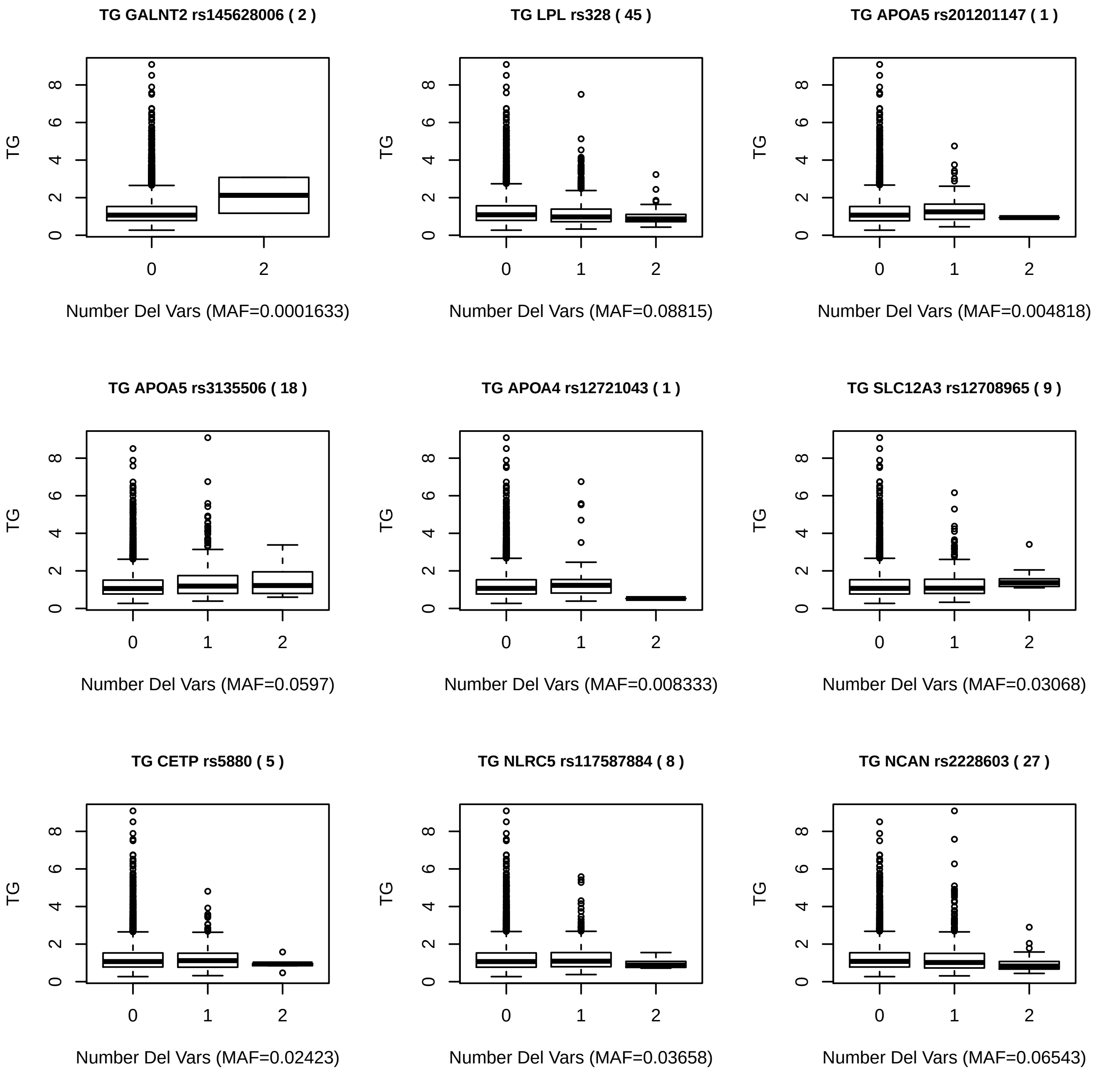
TG GALNT2 rs145628006 (2)
Number Del Vars (MAF=0.0001633)
TG LPL rs328 (45)
Number Del Vars (MAF=0.08815)
TG APOA5 rs201201147 (1)
Number Del Vars (MAF=0.004818)
TG APOA5 rs3135506 (18)
Number Del Vars (MAF=0.0597)
TG APOA4 rs12721043 (1)
Number Del Vars (MAF=0.008333)
TG SLC12A3 rs12708965 (9)
Number Del Vars (MAF=0.03068)
TG CETP rs5880 (5)
Number Del Vars (MAF=0.02423)
TG NLRC5 rs117587884 (8)
Number Del Vars (MAF=0.03658)
TG NCAN rs2228603 (27)
Number Del Vars (MAF=0.06543)
TG
0
1
2
4
6
8

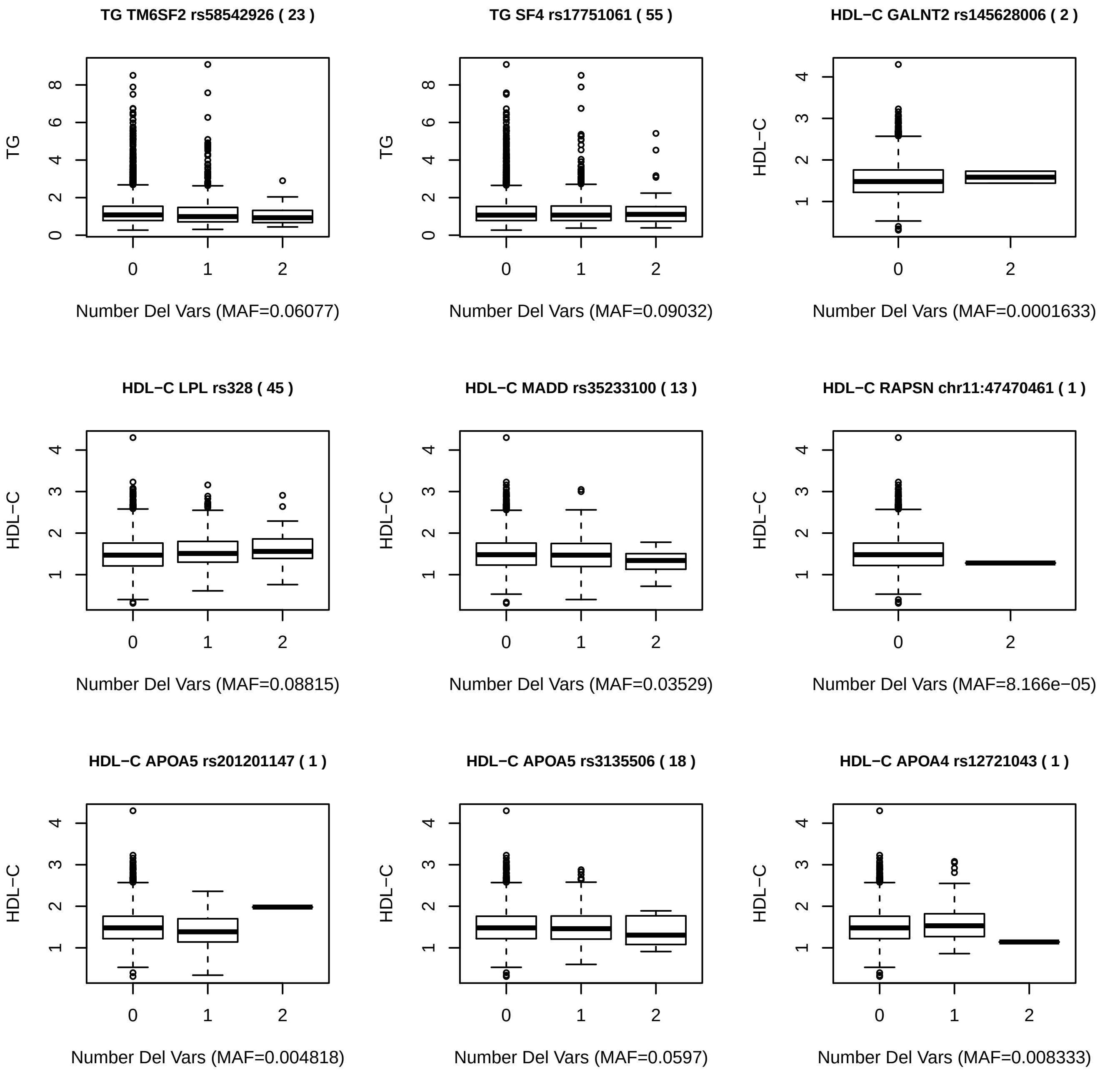
TG TM6SF2 rs58542926 (23)
TG
Number Del Vars (MAF=0.06077)
TG SF4 rs17751061 (55)
TG
Number Del Vars (MAF=0.09032)
HDL−C GALNT2 rs145628006 (2)
HDL−C
Number Del Vars (MAF=0.0001633)
HDL−C LPL rs328 (45)
HDL−C
Number Del Vars (MAF=0.08815)
HDL−C MADD rs35233100 (13)
HDL−C
Number Del Vars (MAF=0.03529)
HDL−C RAPSN chr11:47470461 (1)
HDL−C
Number Del Vars (MAF=8.166e−05)
HDL−C APOA5 rs201201147 (1)
HDL−C
Number Del Vars (MAF=0.004818)
HDL−C APOA5 rs3135506 (18)
HDL−C
Number Del Vars (MAF=0.0597)
HDL−C APOA4 rs12721043 (1)
HDL−C
Number Del Vars (MAF=0.008333)

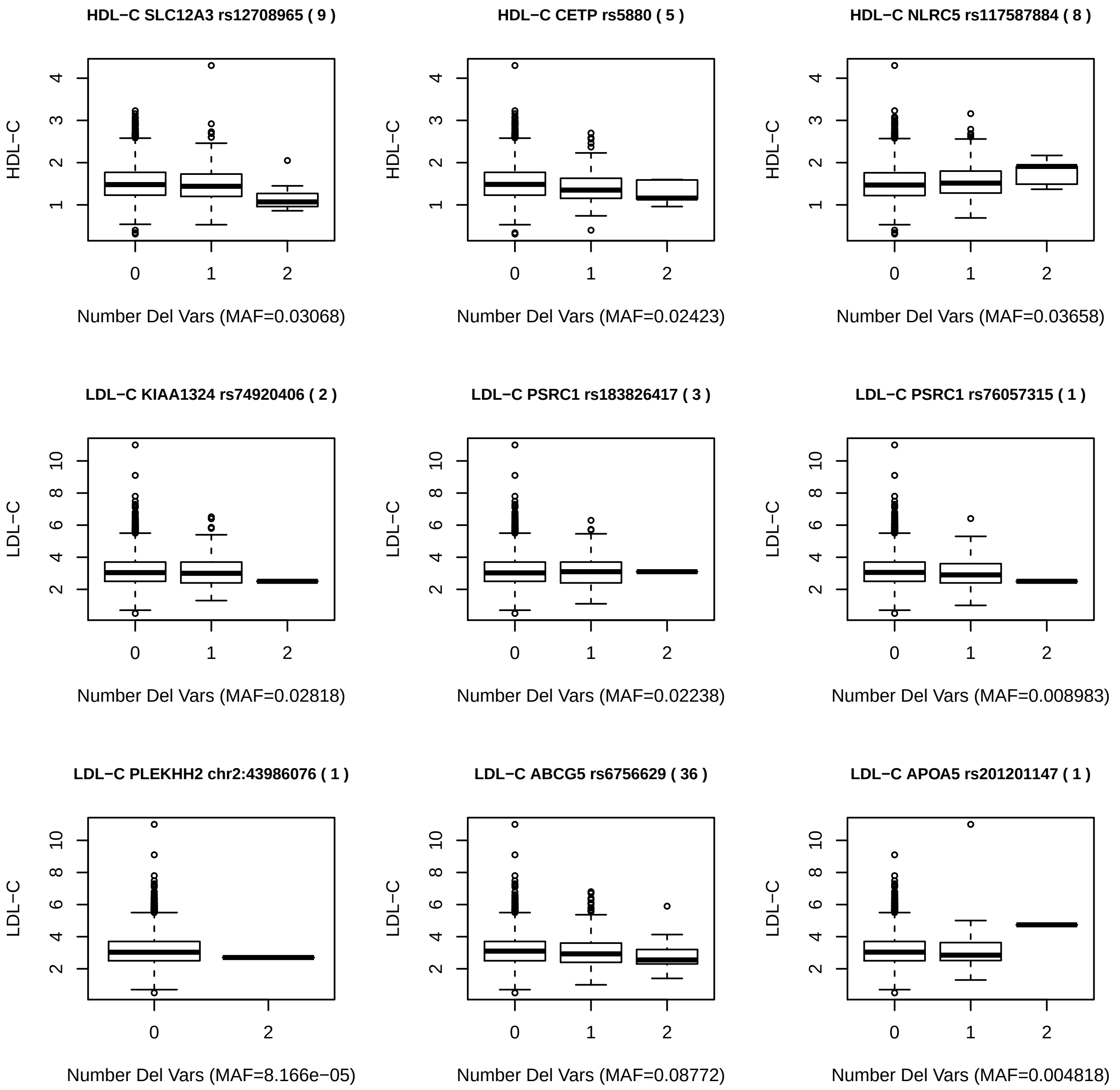
HDL−C SLC12A3 rs12708965 (9)
HDL−C CETP rs5880 (5)
HDL−C NLRC5 rs117587884 (8)
HDL−C
Number Del Vars (MAF=0.03068)
Number Del Vars (MAF=0.02423)
Number Del Vars (MAF=0.03658)
LDL−C KIAA1324 rs74920406 (2)
LDL−C PSRC1 rs183826417 (3)
LDL−C PSRC1 rs76057315 (1)
LDL−C
Number Del Vars (MAF=0.02818)
Number Del Vars (MAF=0.02238)
Number Del Vars (MAF=0.008983)
LDL−C PLEKHH2 chr2:43986076 (1)
LDL−C ABCG5 rs6756629 (36)
LDL−C APOA5 rs201201147 (1)
Number Del Vars (MAF=8.166e−05)
Number Del Vars (MAF=0.08772)
Number Del Vars (MAF=0.004818)

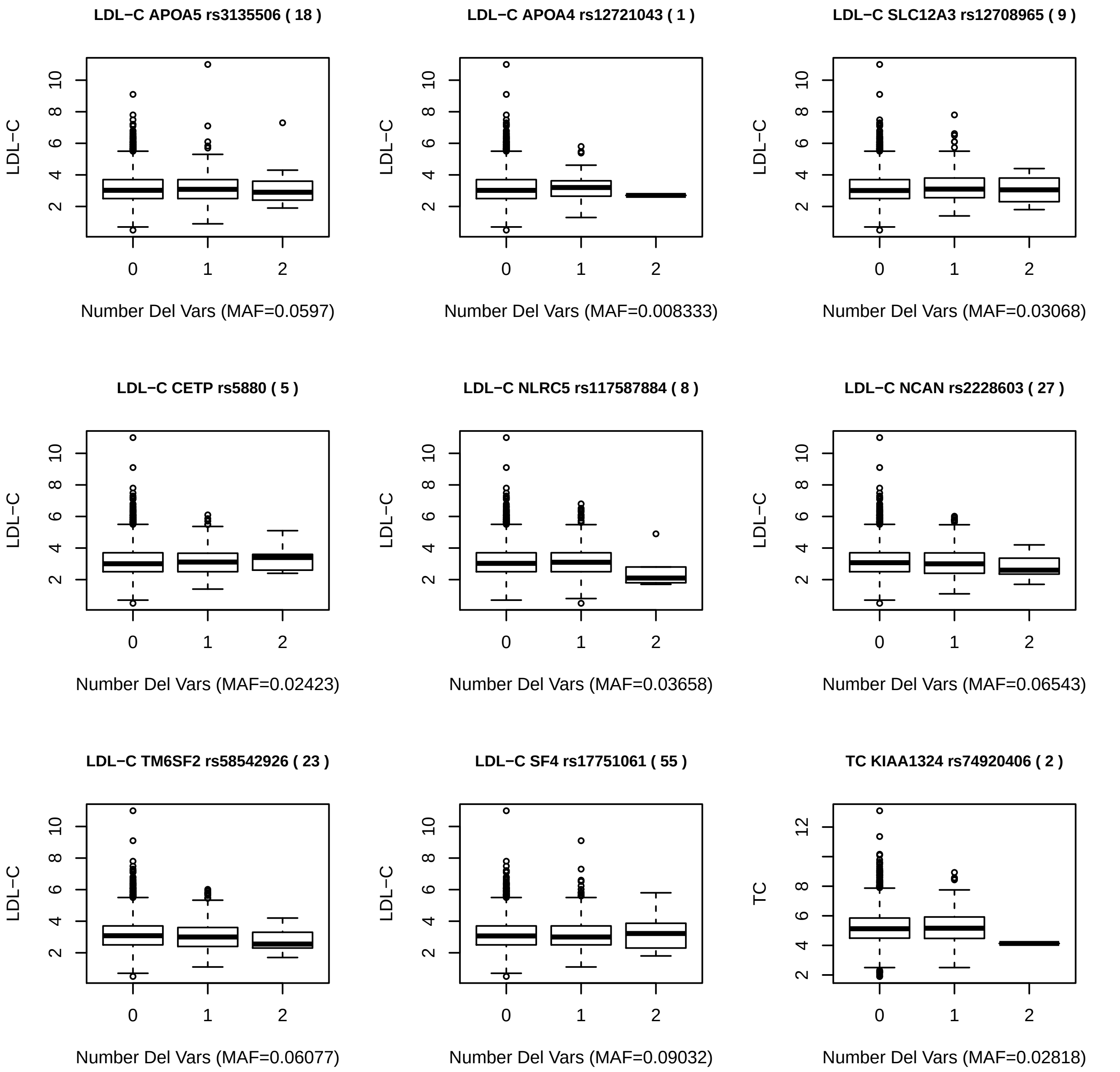
LDL−C APOA5 rs3135506 (18)
LDL−C
Number Del Vars (MAF=0.0597)
LDL−C APOA4 rs12721043 (1)
LDL−C
Number Del Vars (MAF=0.008333)
LDL−C SLC12A3 rs12708965 (9)
LDL−C
Number Del Vars (MAF=0.03068)
LDL−C CETP rs5880 (5)
LDL−C
Number Del Vars (MAF=0.02423)
LDL−C NLRC5 rs117587884 (8)
LDL−C
Number Del Vars (MAF=0.03658)
LDL−C NCAN rs2228603 (27)
LDL−C
Number Del Vars (MAF=0.06543)
LDL−C TM6SF2 rs58542926 (23)
LDL−C
Number Del Vars (MAF=0.06077)
LDL−C SF4 rs17751061 (55)
LDL−C
Number Del Vars (MAF=0.09032)
TC KIAA1324 rs74920406 (2)
TC
Number Del Vars (MAF=0.02818)

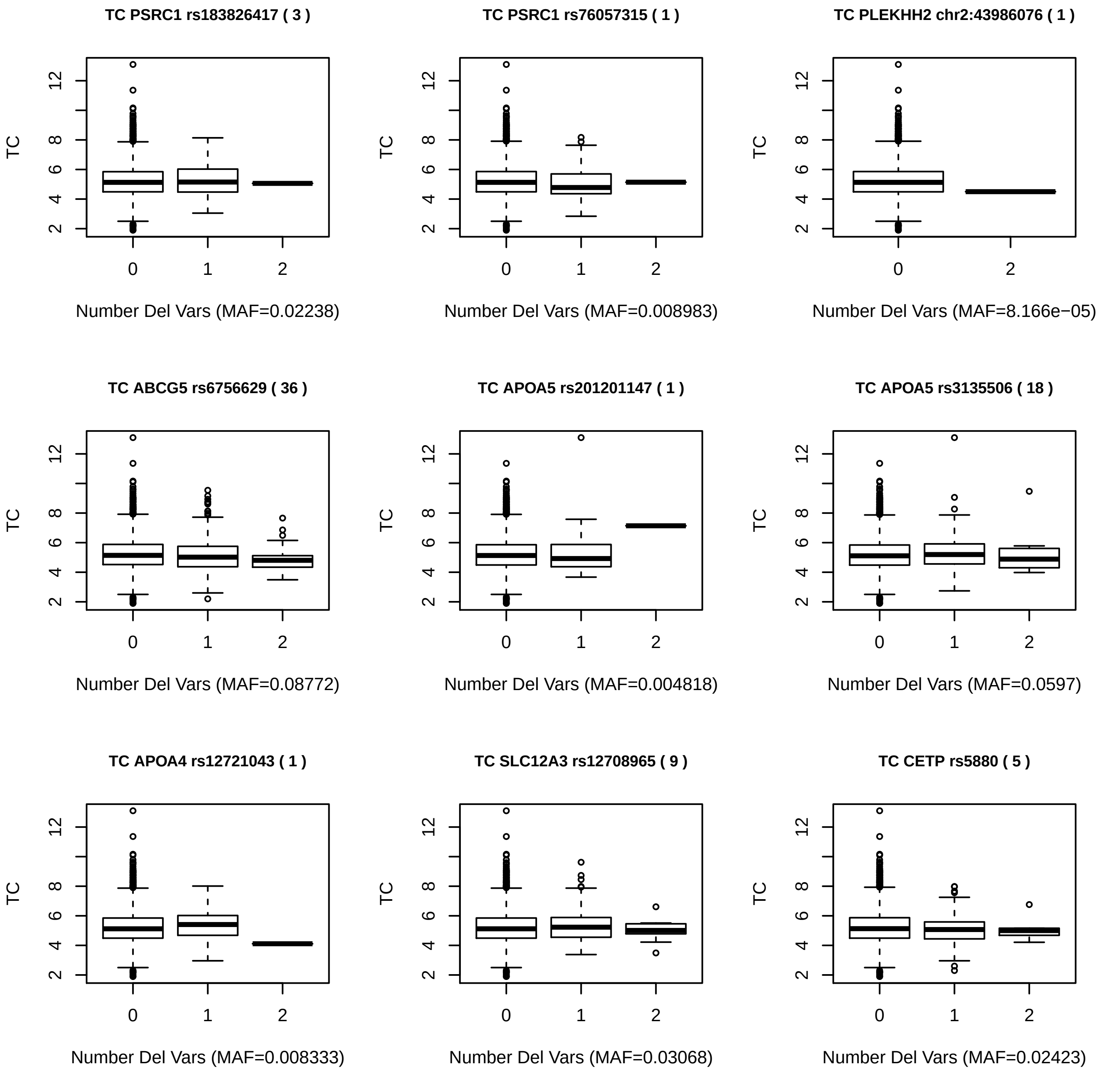
TC PSRC1 rs183826417 (3)
Number Del Vars (MAF=0.02238)
TC PSRC1 rs76057315 (1)
Number Del Vars (MAF=0.008983)
TC PLEKHH2 chr2:43986076 (1)
Number Del Vars (MAF=8.166e−05)
TC ABCG5 rs6756629 (36)
Number Del Vars (MAF=0.08772)
TC APOA5 rs201201147 (1)
Number Del Vars (MAF=0.004818)
TC APOA5 rs3135506 (18)
Number Del Vars (MAF=0.0597)
TC APOA4 rs12721043 (1)
Number Del Vars (MAF=0.008333)
TC SLC12A3 rs12708965 (9)
Number Del Vars (MAF=0.03068)
TC CETP rs5880 (5)
Number Del Vars (MAF=0.02423)
TC

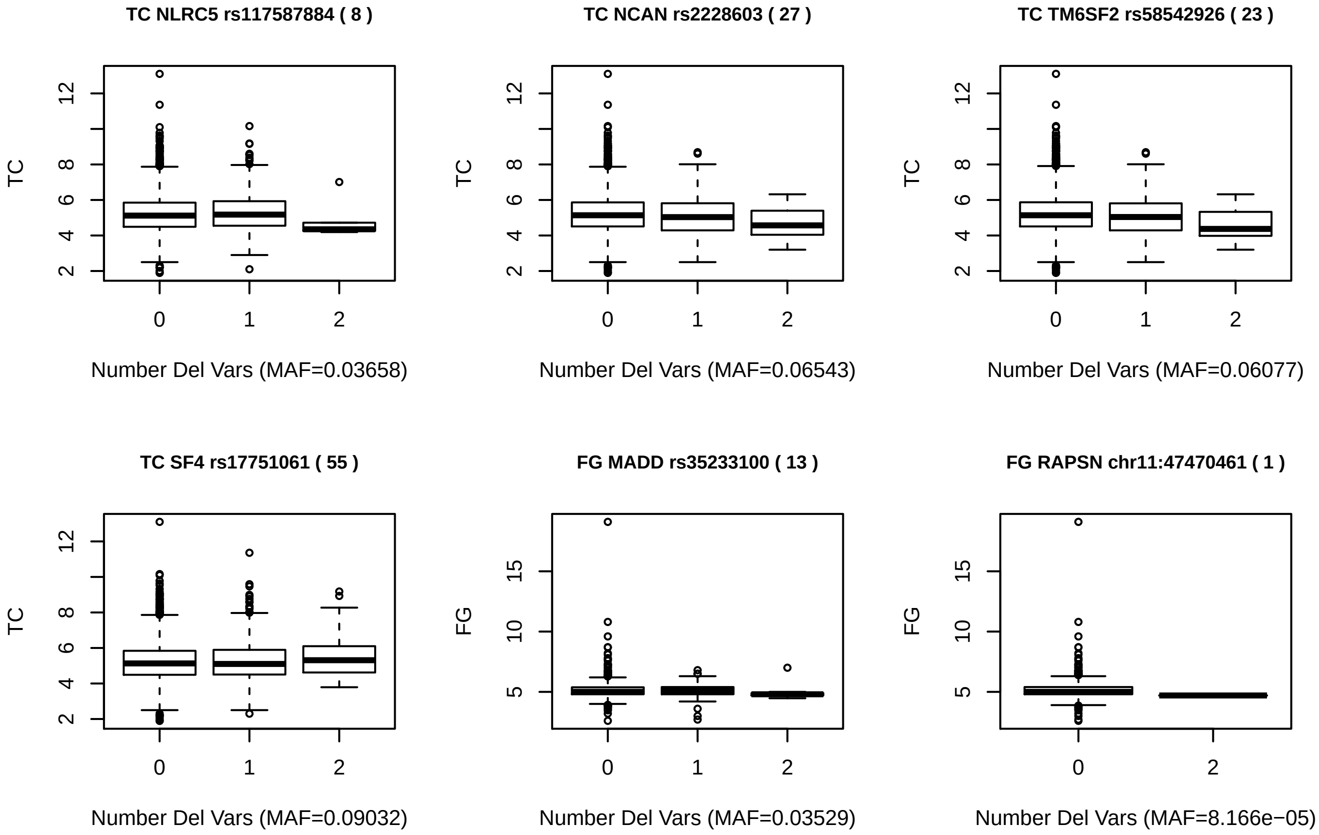
TC NLRC5 rs117587884 (8)
Number Del Vars (MAF=0.03658)
TC NCAN rs2228603 (27)
Number Del Vars (MAF=0.06543)
TC TM6SF2 rs58542926 (23)
Number Del Vars (MAF=0.06077)
TC SF4 rs17751061 (55)
Number Del Vars (MAF=0.09032)
FG MADD rs35233100 (13)
Number Del Vars (MAF=0.03529)
FG RAPSN chr11:47470461 (1)
Number Del Vars (MAF=8.166e−05)
TC
FG
