# Supplementary material for: Re-sequencing Expands Our Understanding of the Phenotypic Impact of Variants at GWAS Loci
Source: PLoS Genet. 2014 Jan 30;10(1):e1004147. doi: 10.1371/journal.pgen.1004147 (PMC3907339; doi:10.1371/journal.pgen.1004147)
Supplement: Figure S6 — Regions and phenotypes where significant association with single SNP analysis of SNPs with MAF>0.11% was independent of the array SNP association. For each phenotype-region combination two plots are presented, single-SNP association, and single-SNP association conditional on the array SNP (in purple, and labeled with text). Color scale is LD relative to the array SNP. On the y-axis is the −log10(P-value) for association to the indicated phenotype, on the x-axis is position in Mb from hg19. Up triangles: nonsense variants; down triangles: missense variants; squares: synonymous and utr variants; circles: no annotation available. The loci are named according to the first gene in the region of interest, starting at the 5′ end of the region. A. LDL-C in ABCG8 locus B. LDL-C in ABCG8 locus, conditional analysis C. FG in G6PC2 locus D. FG in G6PC2 locus, conditional analysis E. HDL-C in LPL locus F. HDL-C in LPL locus, conditional analysis G. TG in LPL locus H. TG in LPL locus, conditional analysis I. HDL-C in ABCA1 locus J. HDL-C in ABCA1 locus, conditional analysis K. TG in APOA1 locus L. TG in APOA1 locus, conditional analysis M. LDL-C in APOA1 locus N. LDL-C in APOA1 locus, conditional analysis O. TC in APOA1 locus P. TC in APOA1 locus, conditional analysis Q. TG in LIPC locus R. TG in LIPC locus, conditional analysis S. HDL-C in LIPC locus T. HDL-C in LIPC locus, conditional analysis U. HDL-C in CETP locus V. HDL-C in CETP locus, conditional analysis. (PDF) [file pgen.1004147.s006.pdf]

Figure S6A

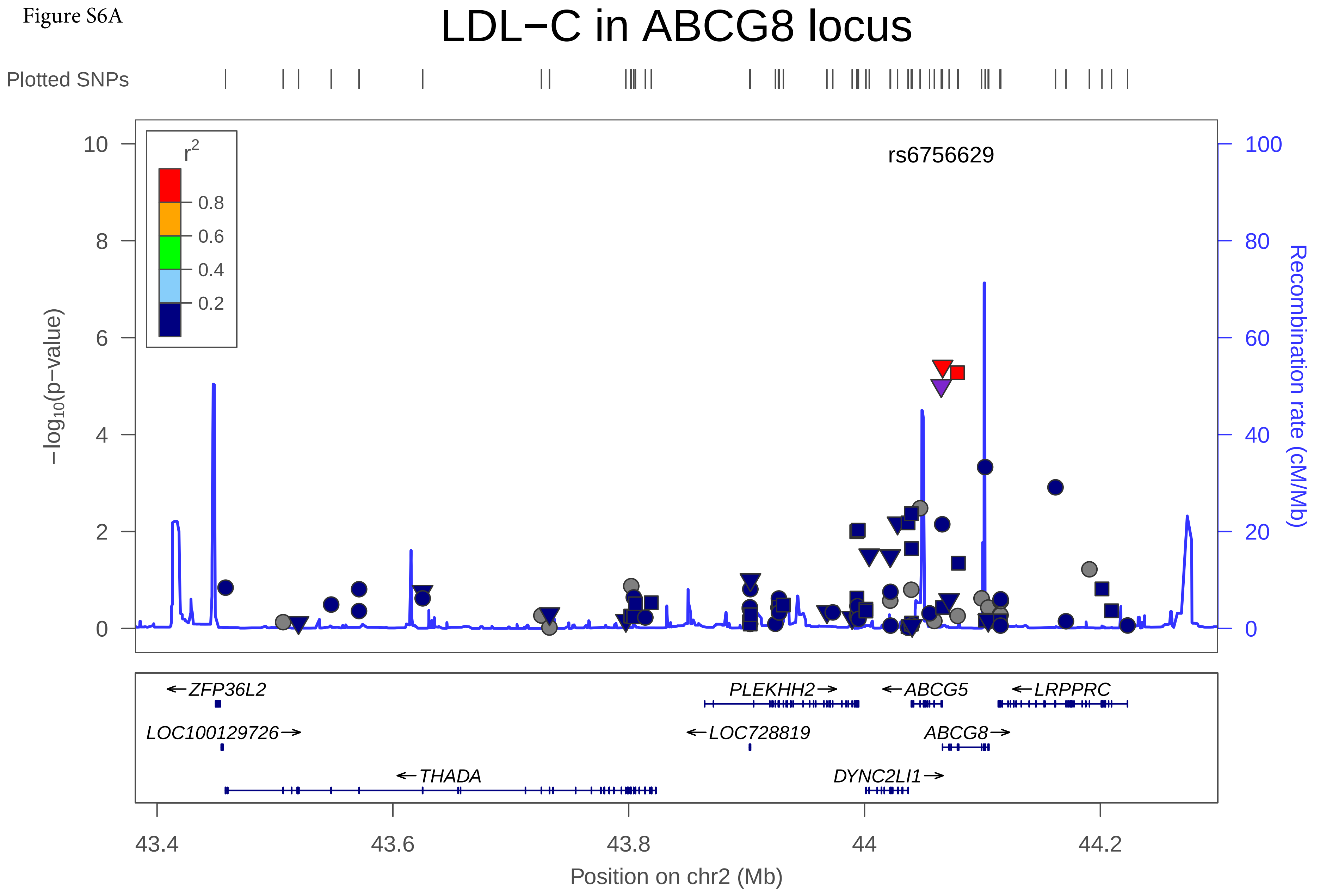

LDL−C in ABCG8 locus
Plotted SNPs
rs6756629
r2
0.8
0.6
0.4
0.2
10
8
6
4
2
0
100
80
60
40
20
0
−log10(p−value)
Recombination rate (cM/Mb)
ZFP36L2
PLEKHH2
ABCG5
LRPPRC
LOC100129726
LOC728819
ABCG8
THADA
DYNC2LI1
43.4
43.6
43.8
44
44.2
Position on chr2 (Mb)

Figure S6B

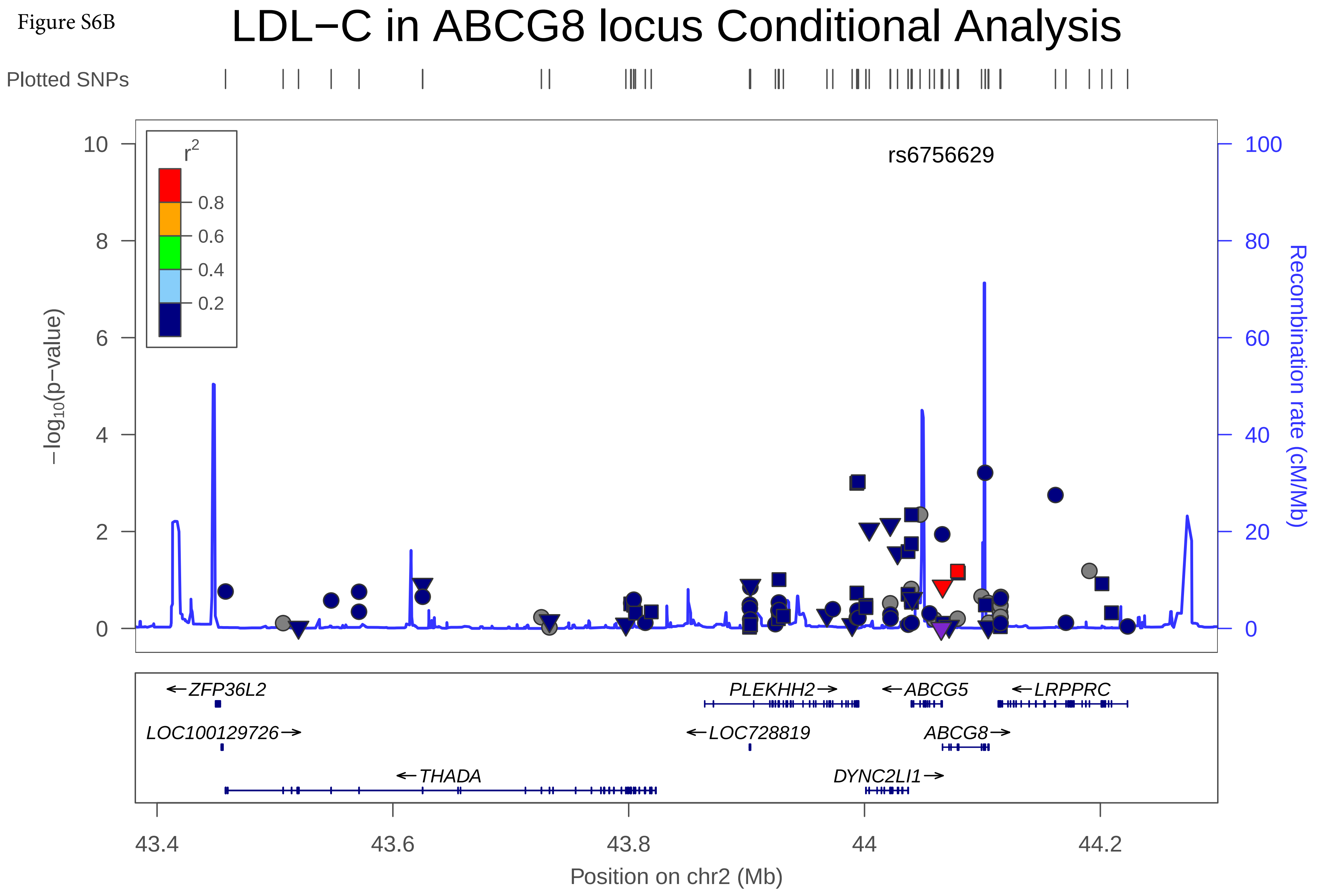

LDL−C in ABCG8 locus Conditional Analysis
Plotted SNPs
rs6756629
r2
0.8
0.6
0.4
0.2
−log10(p−value)
Recombination rate (cM/Mb)
ZFP36L2
LOC100129726
THADA
PLEKHH2
LOC728819
DYNC2LI1
ABCG5
ABCG8
LRPPRC
43.4
43.6
43.8
44
44.2
Position on chr2 (Mb)

Figure S6C

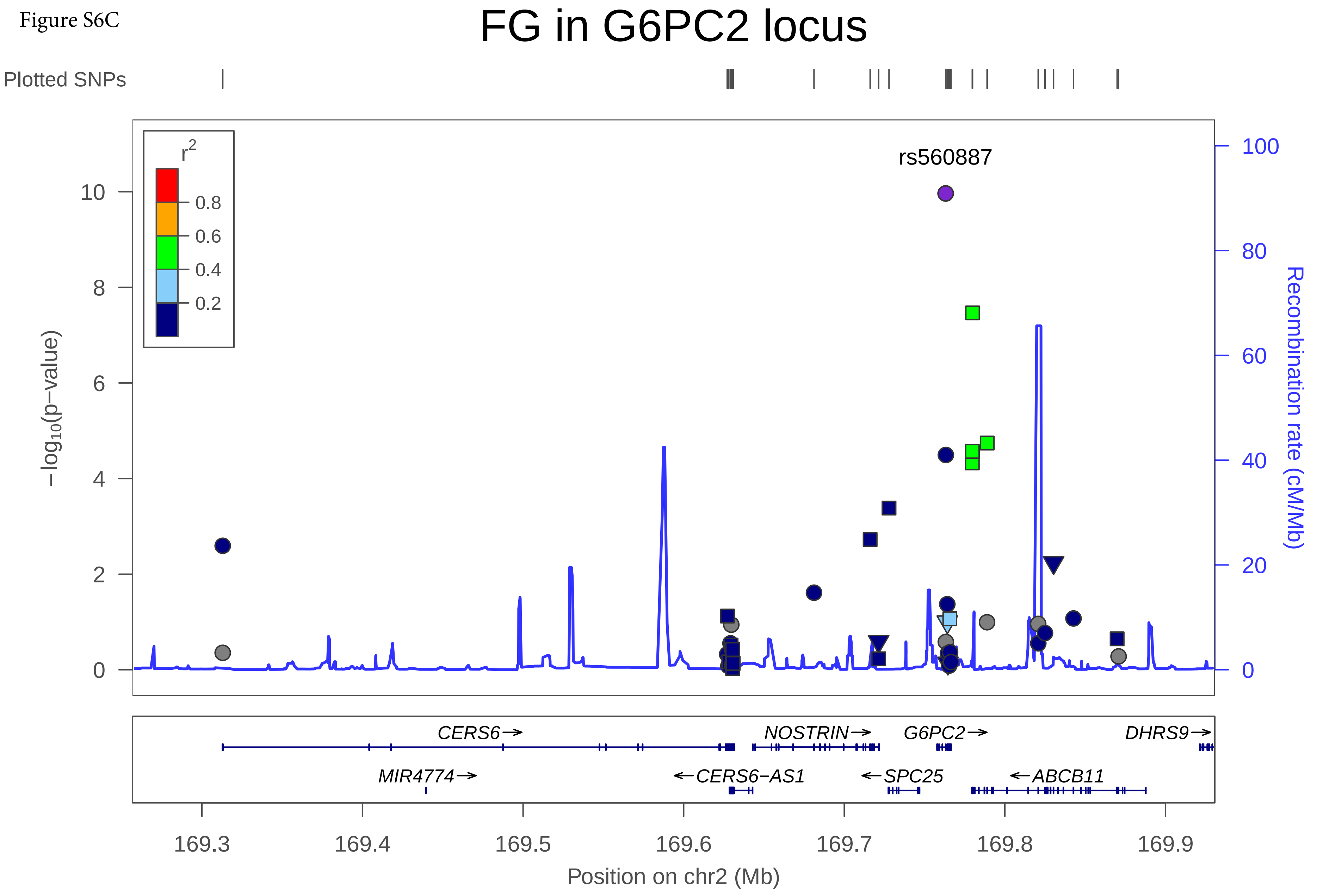

FG in G6PC2 locus
Plotted SNPs
r2
0.8
0.6
0.4
0.2
rs560887
−log10(p−value)
Recombination rate (cM/Mb)
CERS6→
NOSTRIN→
G6PC2→
DHRS9→
MIR4774→
←CERS6−AS1
←SPC25
←ABCB11
169.3
169.4
169.5
169.6
169.7
169.8
169.9
Position on chr2 (Mb)

Figure S6D

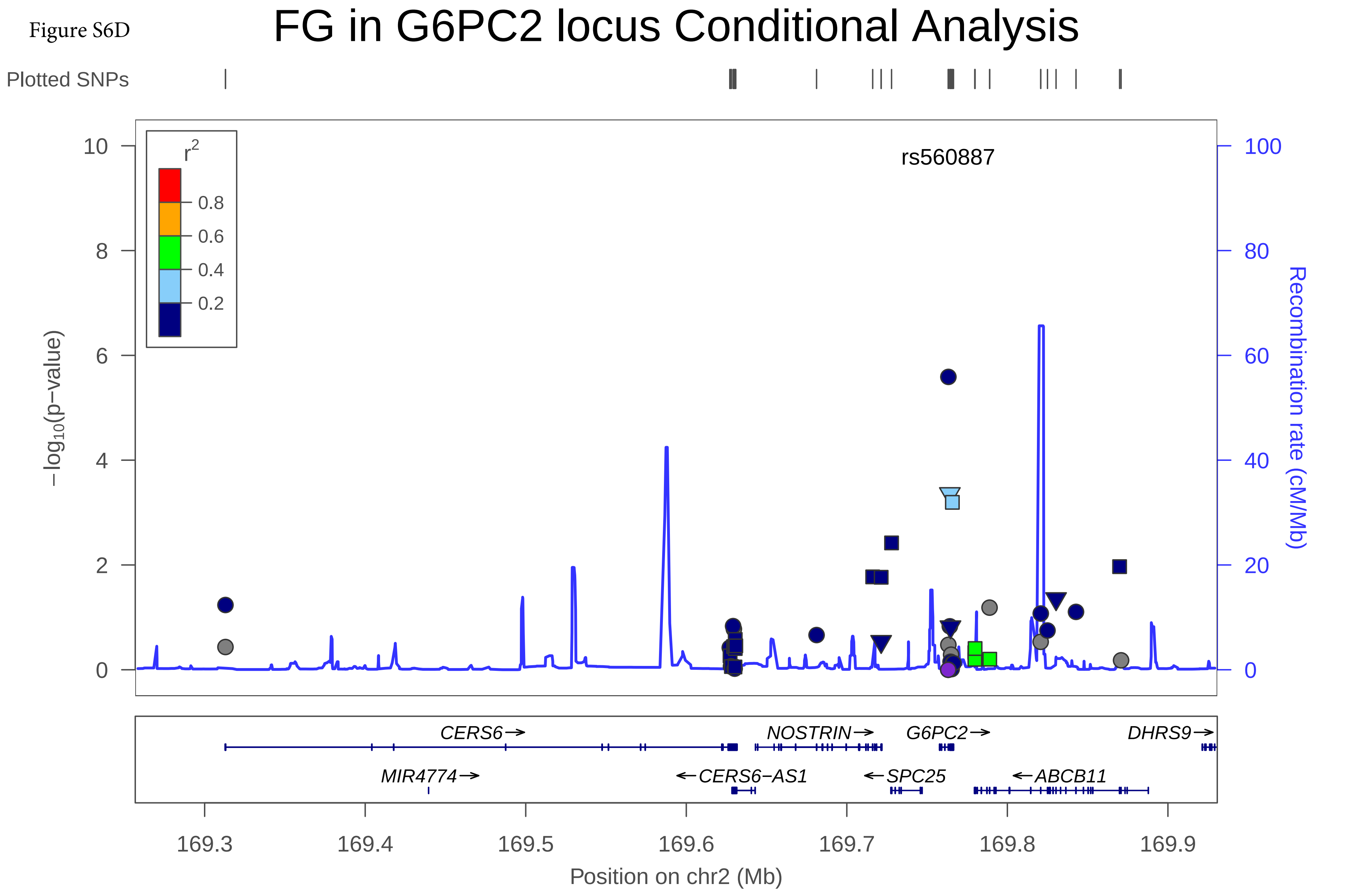

FG in G6PC2 locus Conditional Analysis
Plotted SNPs
r2
0.8
0.6
0.4
0.2
rs560887
−log10(p−value)
Recombination rate (cM/Mb)
0
2
4
6
8
10
0
20
40
60
80
100
CERS6→
NOSTRIN→
G6PC2→
DHRS9→
MIR4774→
←CERS6−AS1
←SPC25
←ABCB11
169.3
169.4
169.5
169.6
169.7
169.8
169.9
Position on chr2 (Mb)

Figure S6E

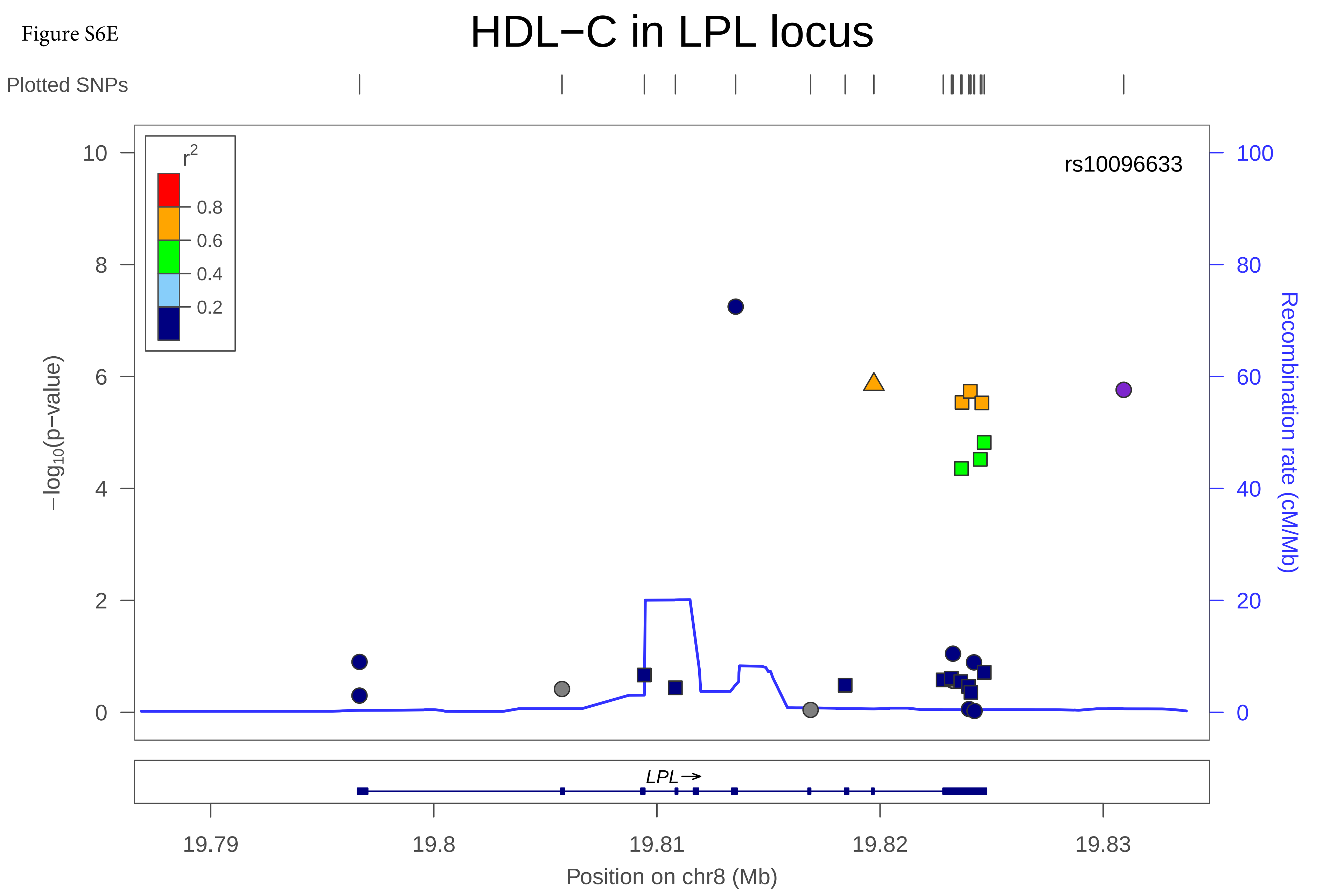
HDL−C in LPL locus
Plotted SNPs
r2
0.8
0.6
0.4
0.2
rs10096633
−log10(p−value)
Recombination rate (cM/Mb)
10
8
6
4
2
0
100
80
60
40
20
0
LPL→
19.79
19.8
19.81
19.82
19.83
Position on chr8 (Mb)

Figure S6F

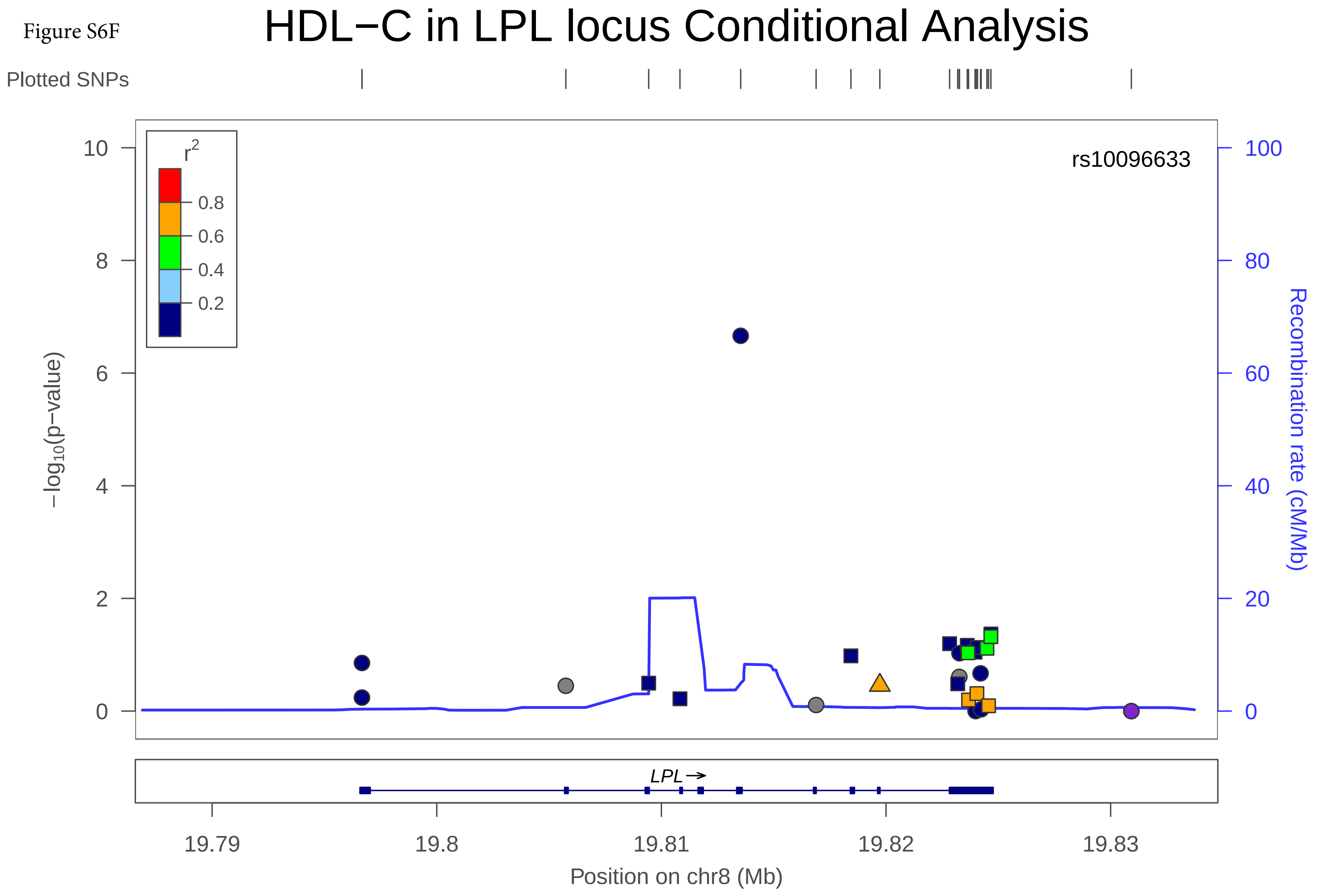

HDL−C in LPL locus Conditional Analysis
Plotted SNPs
r2
0.8
0.6
0.4
0.2
rs10096633
10
8
6
4
2
0
−log10(p−value)
100
80
60
40
20
0
Recombination rate (cM/Mb)
LPL→
19.79
19.8
19.81
19.82
19.83
Position on chr8 (Mb)

Figure S6G

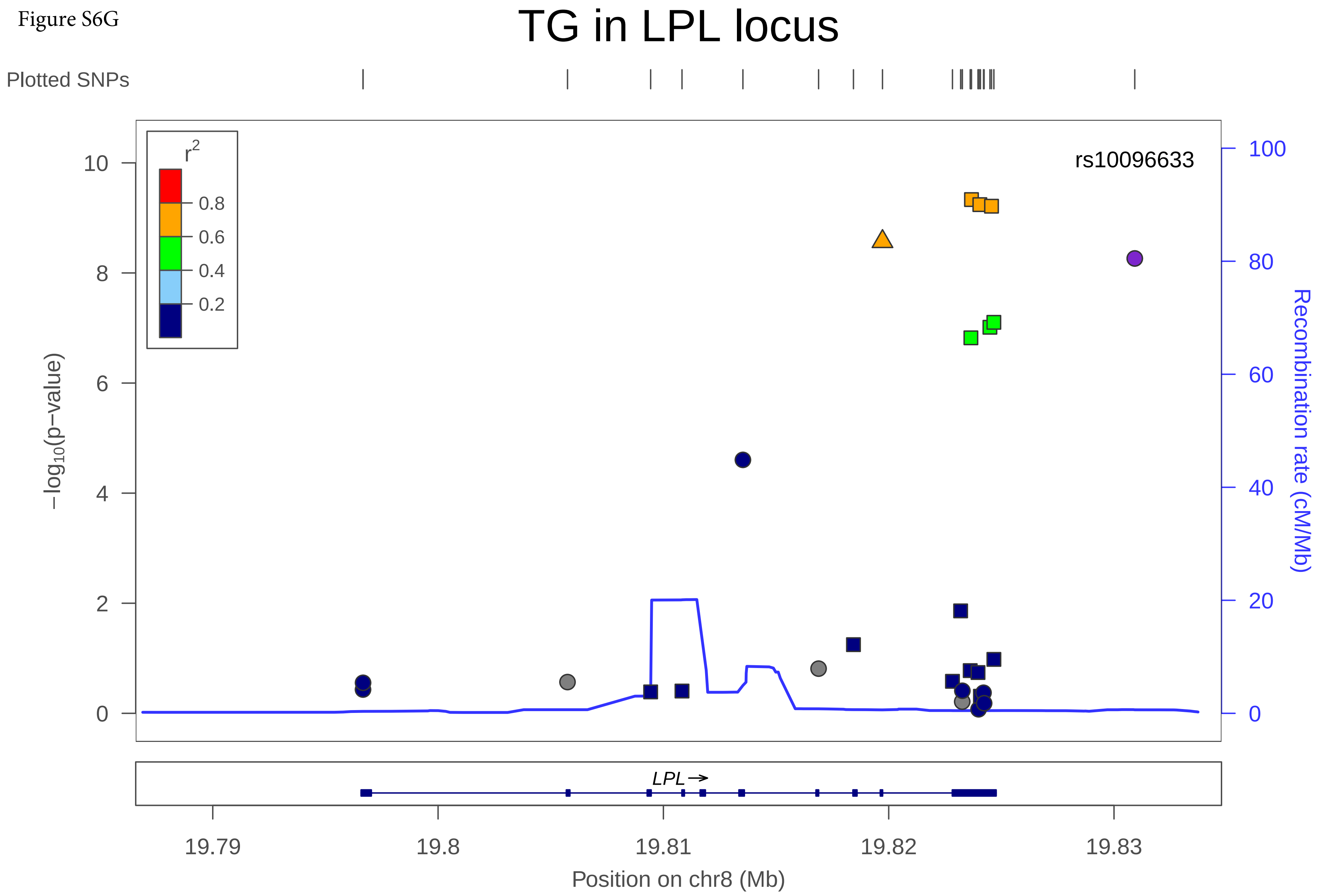

TG in LPL locus
Plotted SNPs
r²
0.8
0.6
0.4
0.2
rs10096633
10
8
6
4
2
0
−log10(p−value)
100
80
60
40
20
0
Recombination rate (cM/Mb)
LPL→
19.79
19.8
19.81
19.82
19.83
Position on chr8 (Mb)

Figure S6H

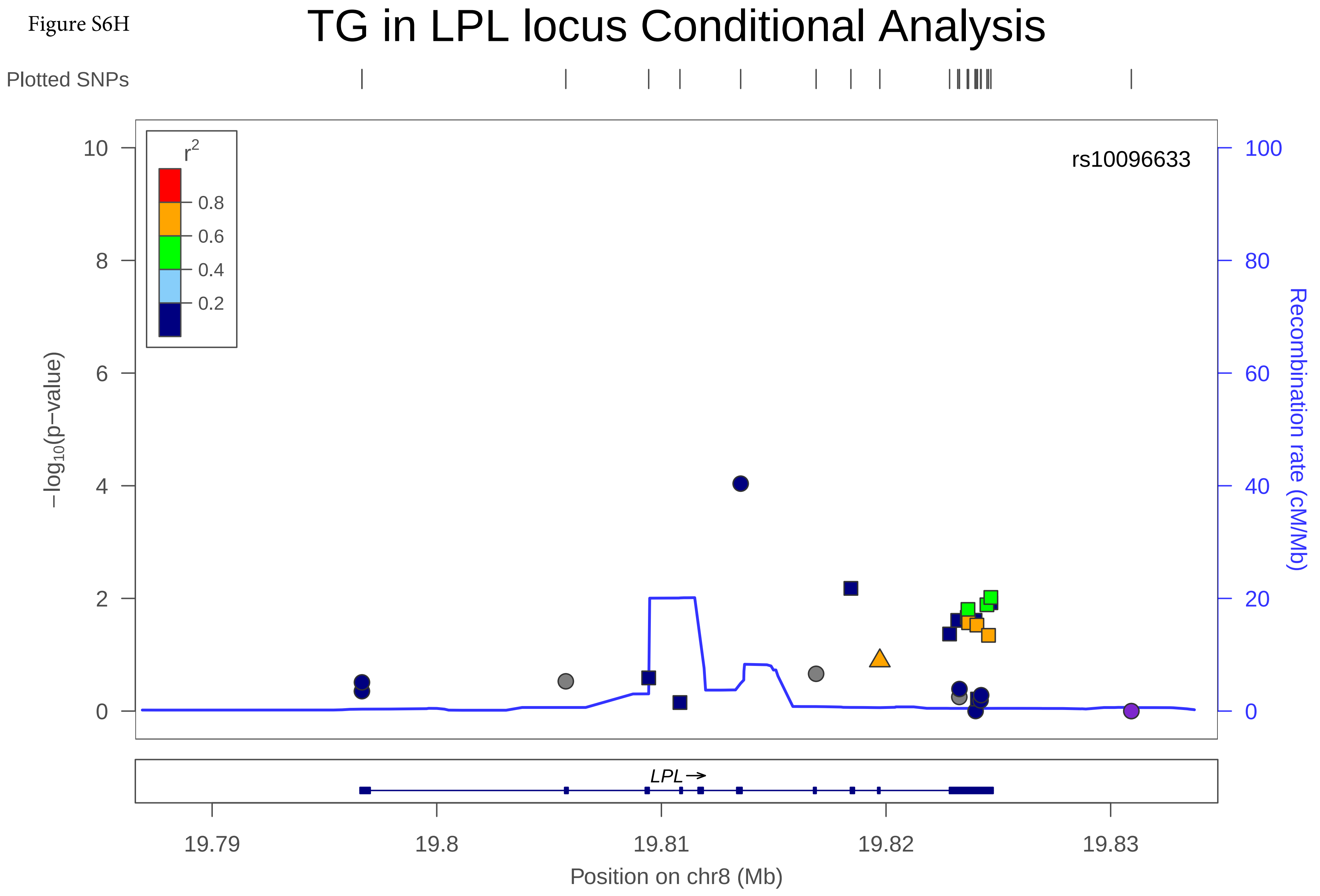

TG in LPL locus Conditional Analysis
Plotted SNPs
r2
0.8
0.6
0.4
0.2
rs10096633
10
8
6
4
2
0
−log10(p−value)
100
80
60
40
20
0
Recombination rate (cM/Mb)
LPL→
19.79
19.8
19.81
19.82
19.83
Position on chr8 (Mb)

Figure S6I

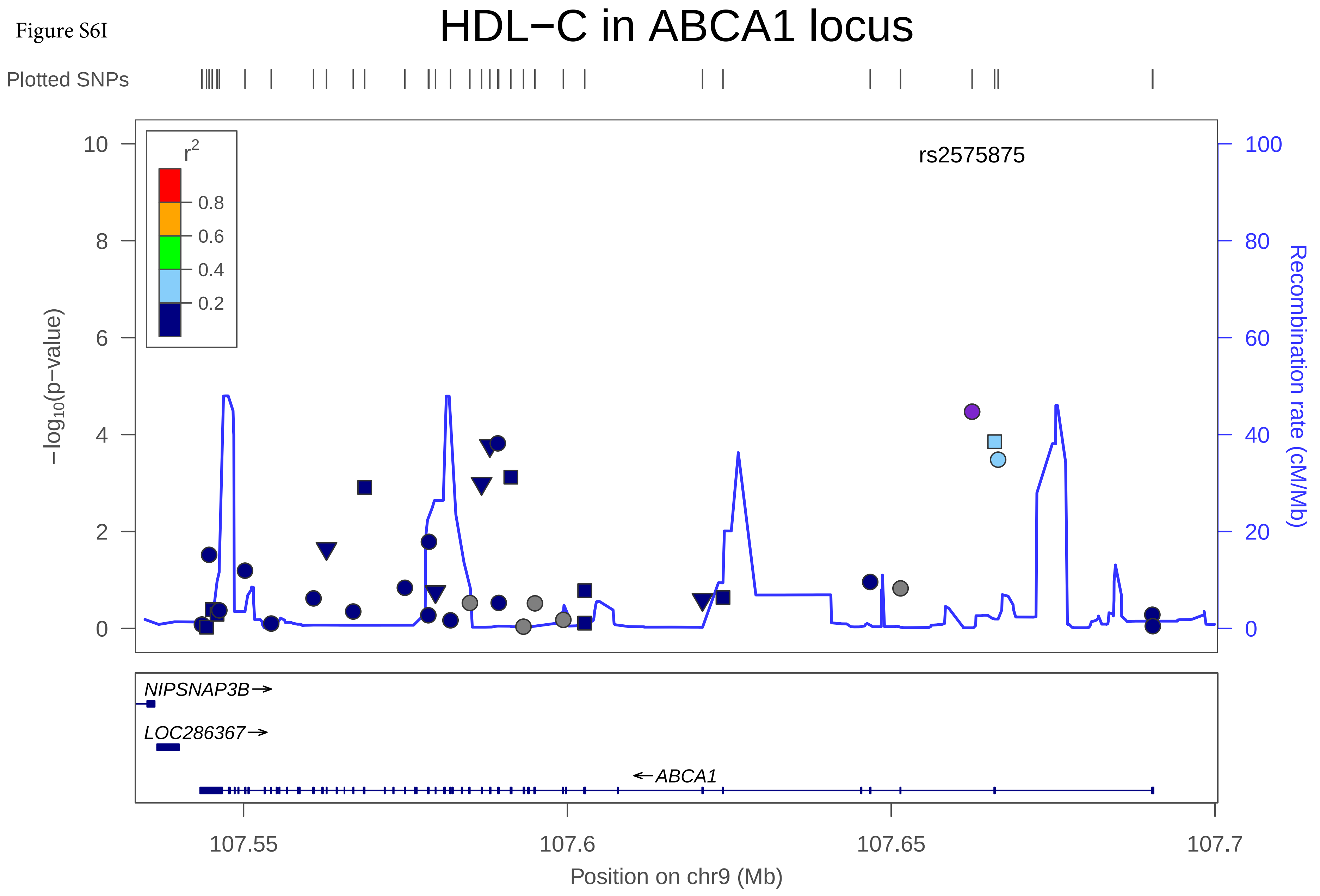

HDL−C in ABCA1 locus
Plotted SNPs
r2
0.8
0.6
0.4
0.2
rs2575875
10
8
6
4
2
0
100
80
60
40
20
0
−log10(p−value)
Recombination rate (cM/Mb)
NIPSNAP3B→
LOC286367→
←ABCA1
107.55
107.6
107.65
107.7
Position on chr9 (Mb)

Figure S6J

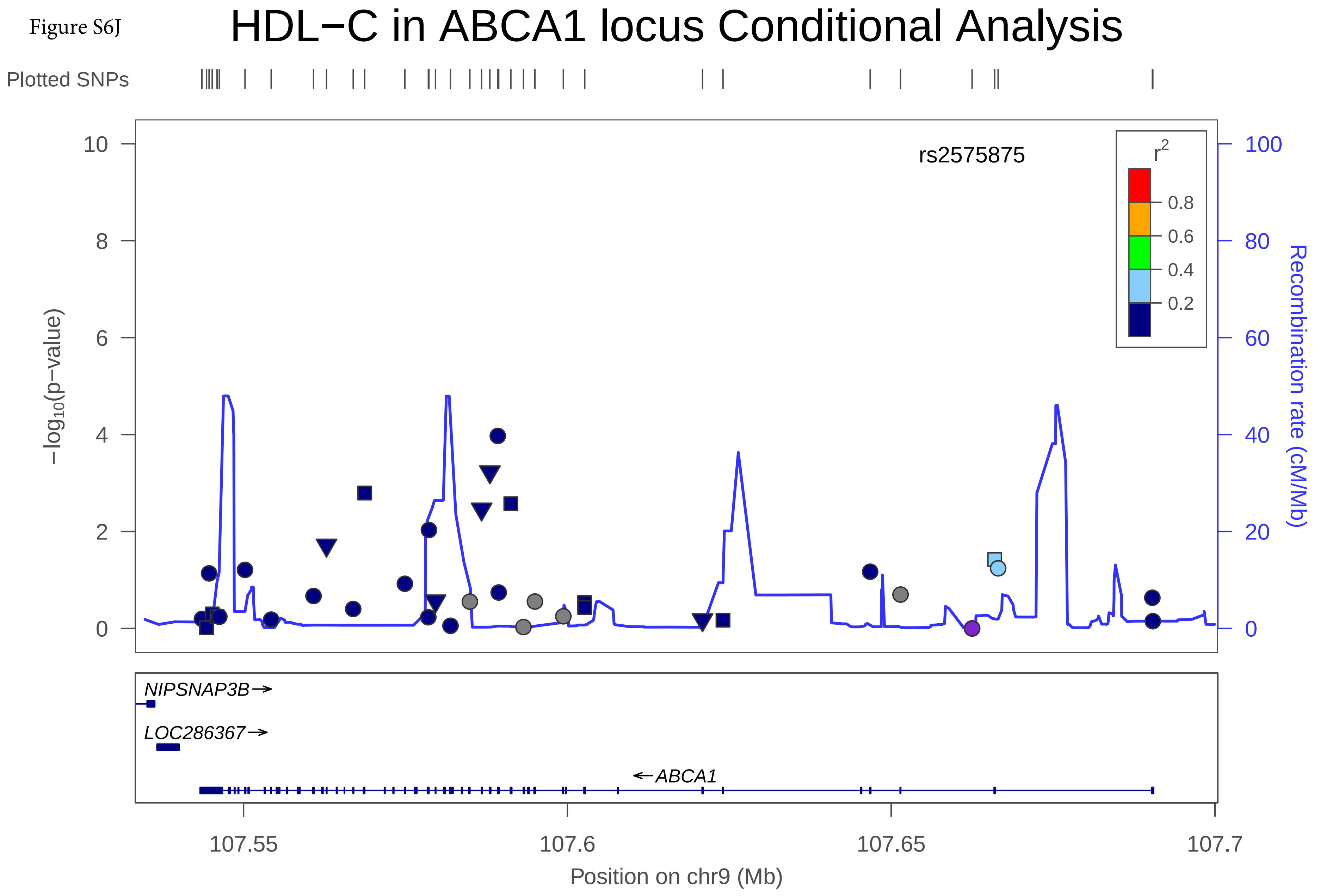
HDL−C in ABCA1 locus Conditional Analysis
Plotted SNPs
rs2575875
r2
0.8
0.6
0.4
0.2
−log10(p−value)
Recombination rate (cM/Mb)
NIPSNAP3B
LOC286367
ABCA1
107.55
107.6
107.65
107.7
Position on chr9 (Mb)

Figure S6K

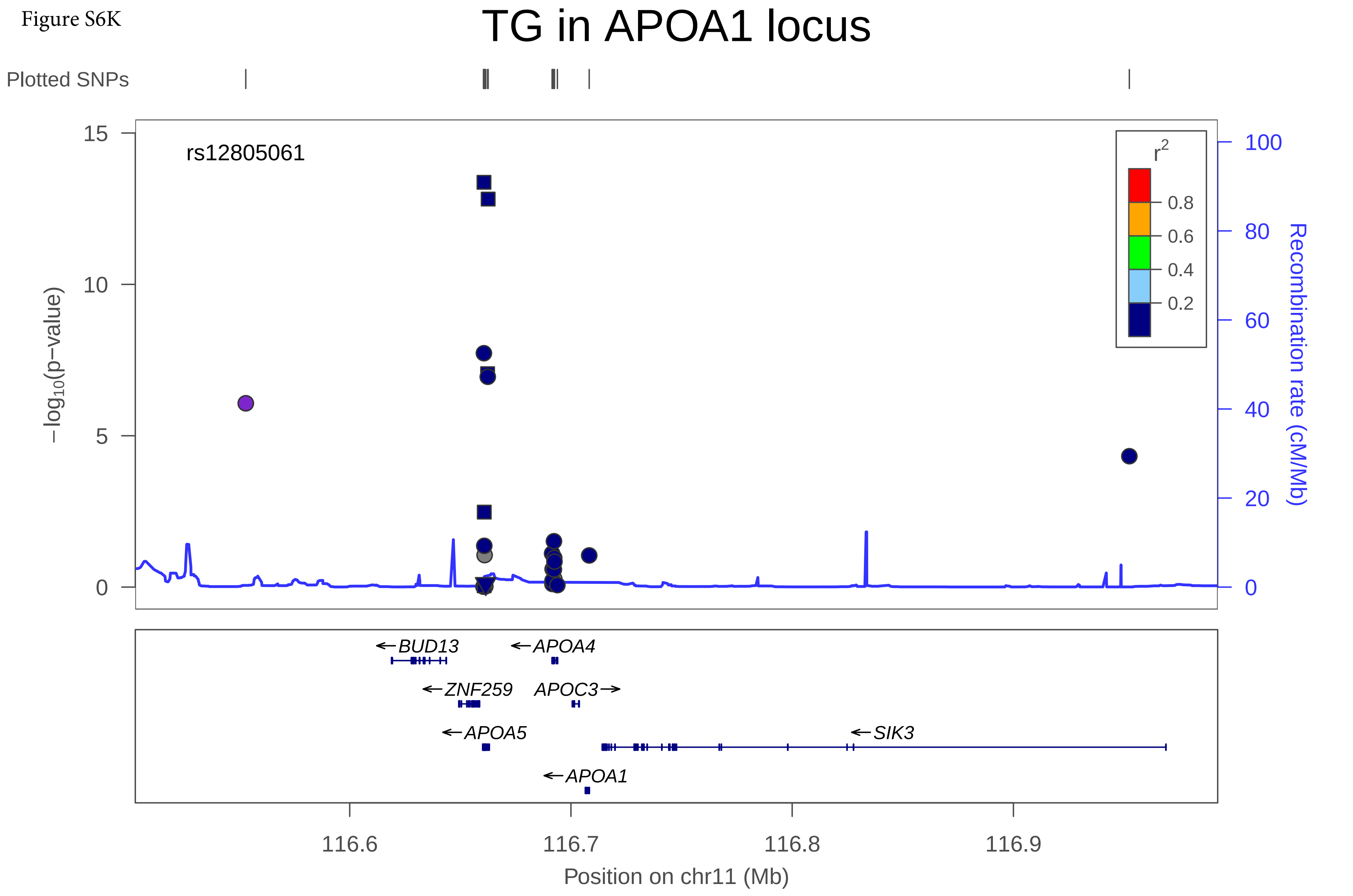

TG in APOA1 locus
Plotted SNPs
rs12805061
r2
0.8
0.6
0.4
0.2
15
10
5
0
−log10(p−value)
100
80
60
40
20
0
Recombination rate (cM/Mb)
BUD13
APOA4
ZNF259
APOC3
APOA5
SIK3
APOA1
116.6
116.7
116.8
116.9
Position on chr11 (Mb)

Figure S6L

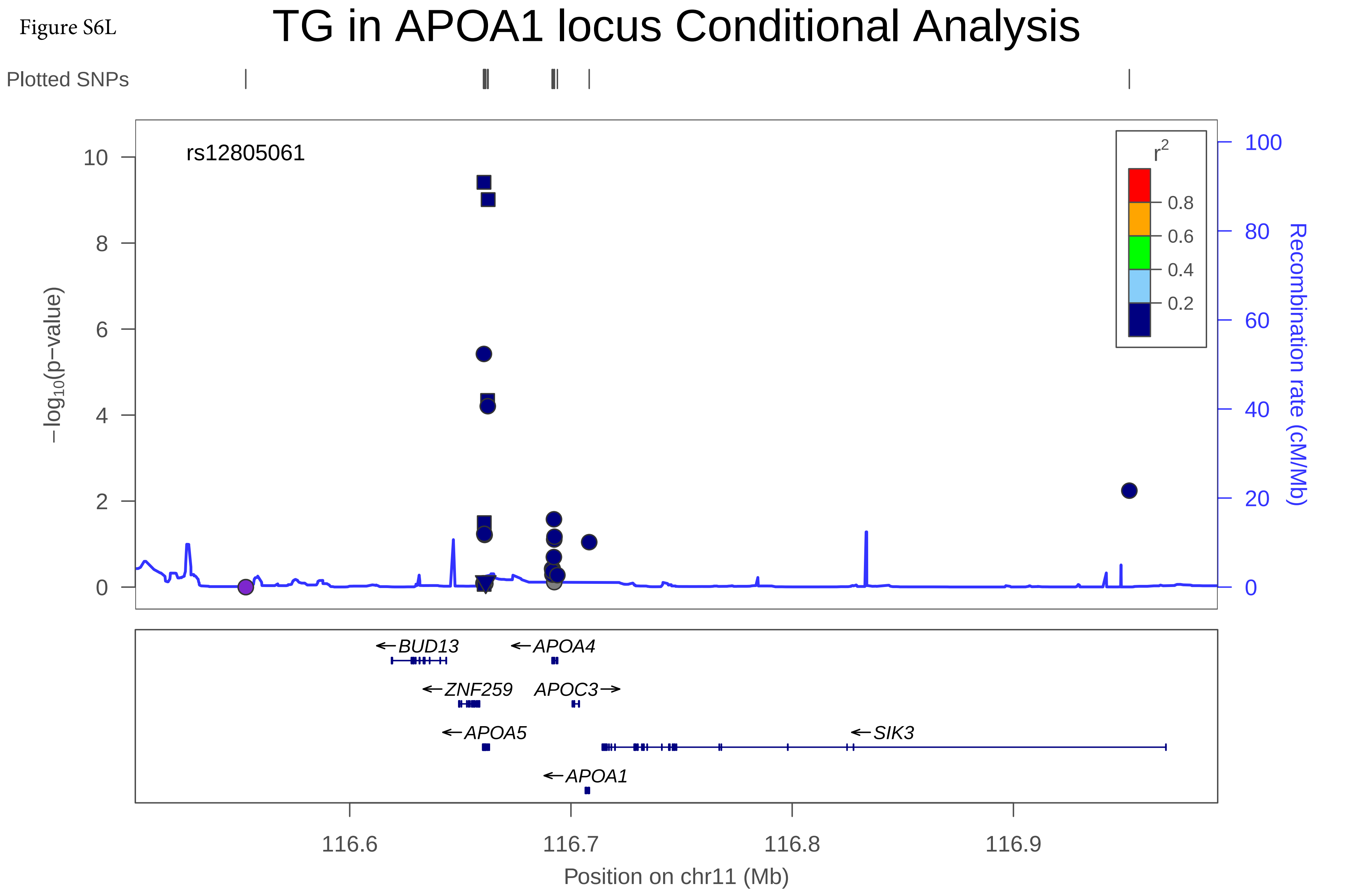

TG in APOA1 locus Conditional Analysis
Plotted SNPs
rs12805061
r2
0.8
0.6
0.4
0.2
10
8
6
4
2
0
−log10(p−value)
100
80
60
40
20
0
Recombination rate (cM/Mb)
BUD13
APOA4
ZNF259
APOC3
APOA5
SIK3
APOA1
116.6
116.7
116.8
116.9
Position on chr11 (Mb)

Figure S6M

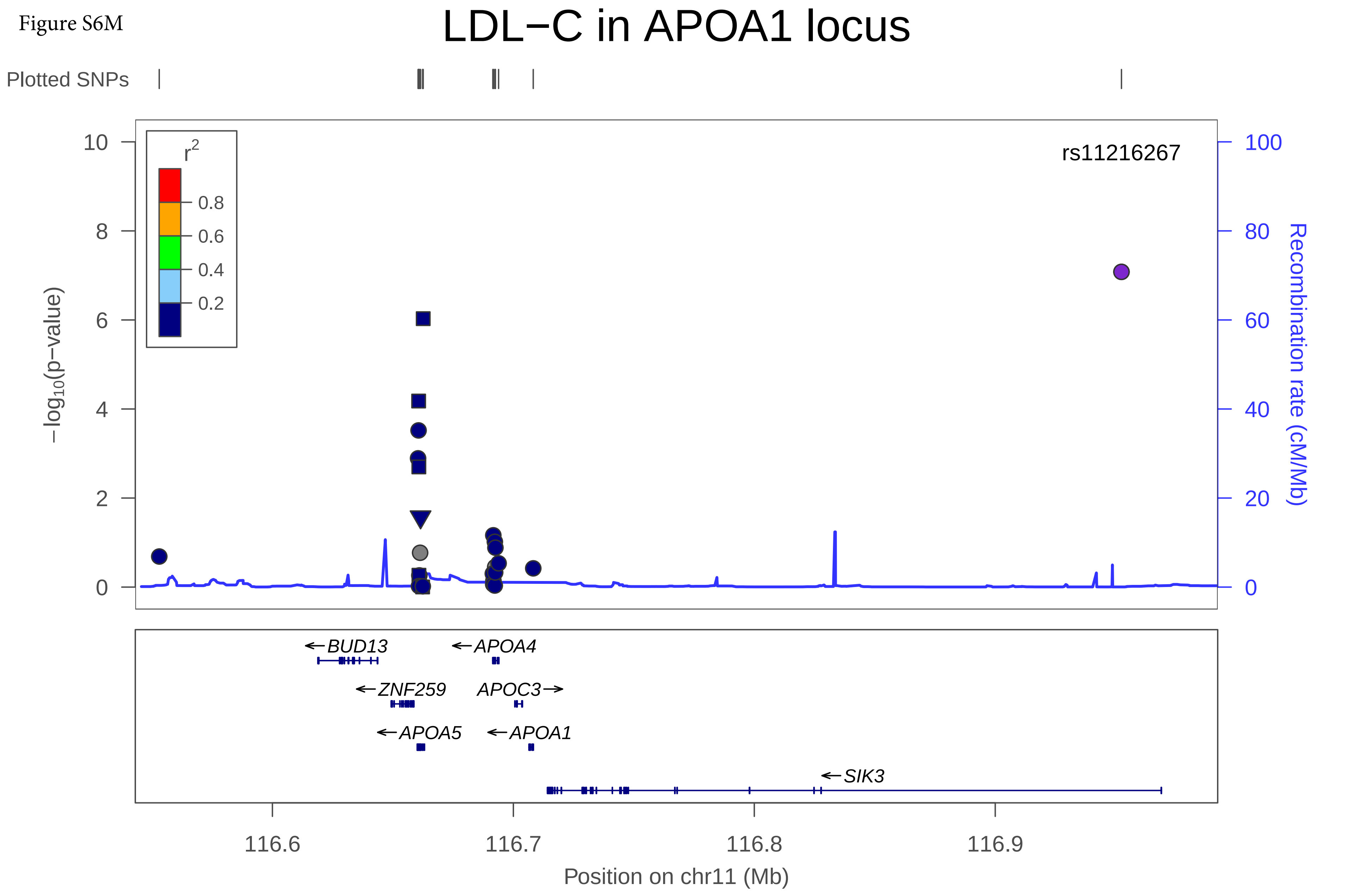
LDL−C in APOA1 locus
Plotted SNPs
rs11216267
r2
0.8
0.6
0.4
0.2
−log10(p−value)
Recombination rate (cM/Mb)
10
8
6
4
2
0
100
80
60
40
20
0
BUD13
APOA4
ZNF259
APOC3
APOA5
APOA1
SIK3
116.6
116.7
116.8
116.9
Position on chr11 (Mb)

Figure S6N

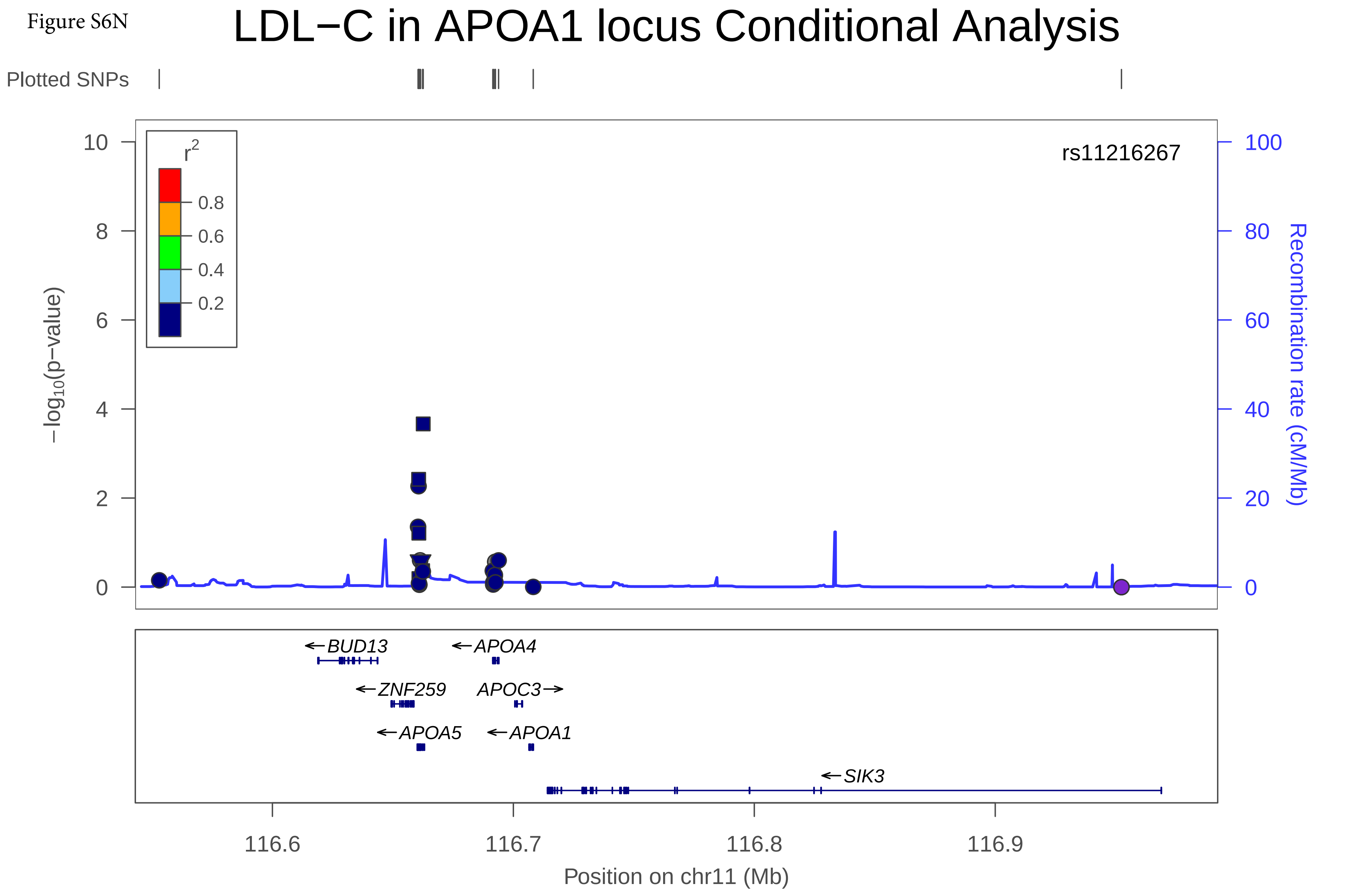

LDL−C in APOA1 locus Conditional Analysis
Plotted SNPs
r2
0.8
0.6
0.4
0.2
rs11216267
−log10(p−value)
Recombination rate (cM/Mb)
10
8
6
4
2
0
100
80
60
40
20
0
BUD13
APOA4
ZNF259
APOC3
APOA5
APOA1
SIK3
116.6
116.7
116.8
116.9
Position on chr11 (Mb)

Figure S6O

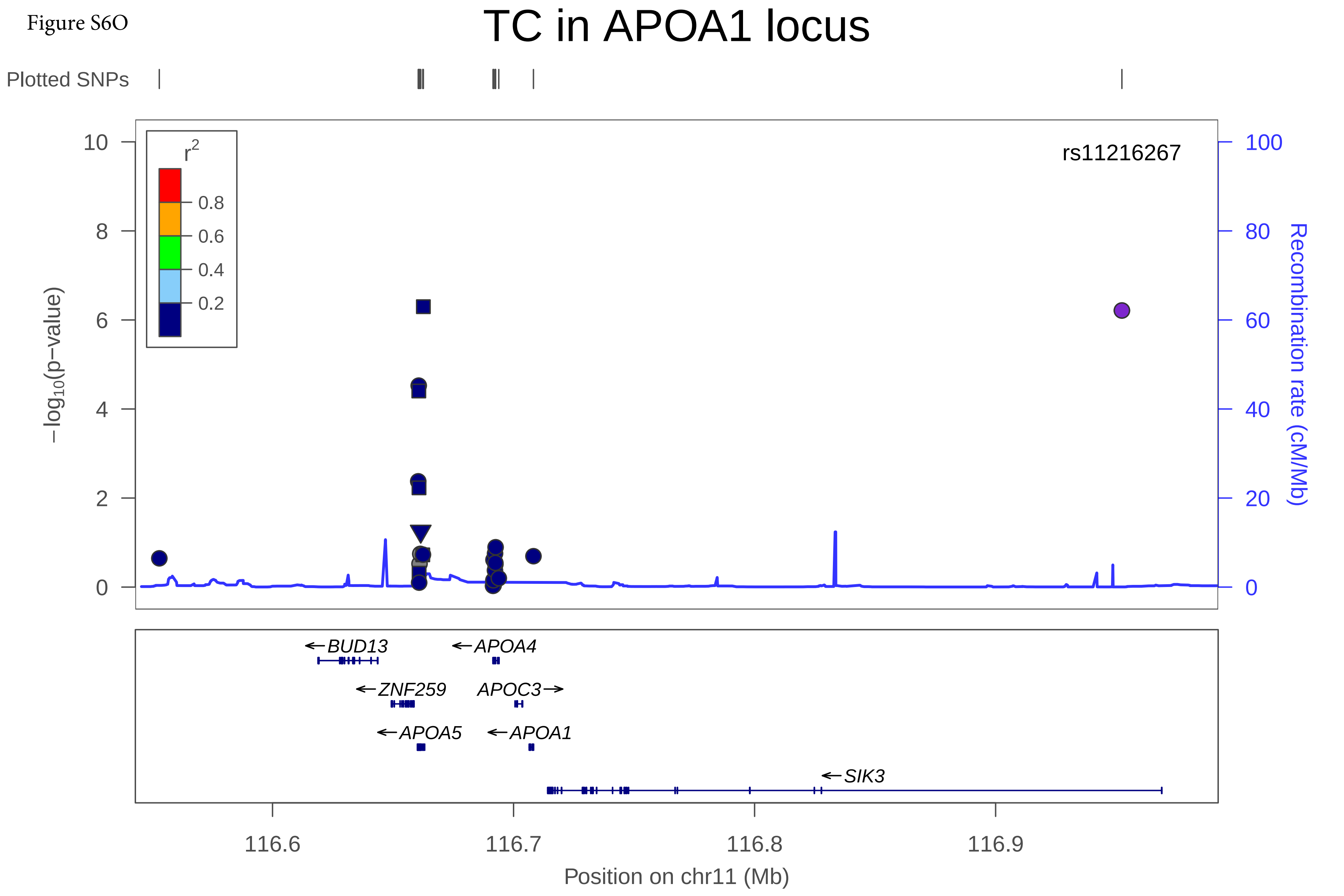
TC in APOA1 locus
Plotted SNPs
rs11216267
r2
0.8
0.6
0.4
0.2
10
8
6
4
2
0
−log10(p−value)
100
80
60
40
20
0
Recombination rate (cM/Mb)
BUD13
APOA4
ZNF259
APOC3
APOA5
APOA1
SIK3
116.6
116.7
116.8
116.9
Position on chr11 (Mb)

Figure S6P

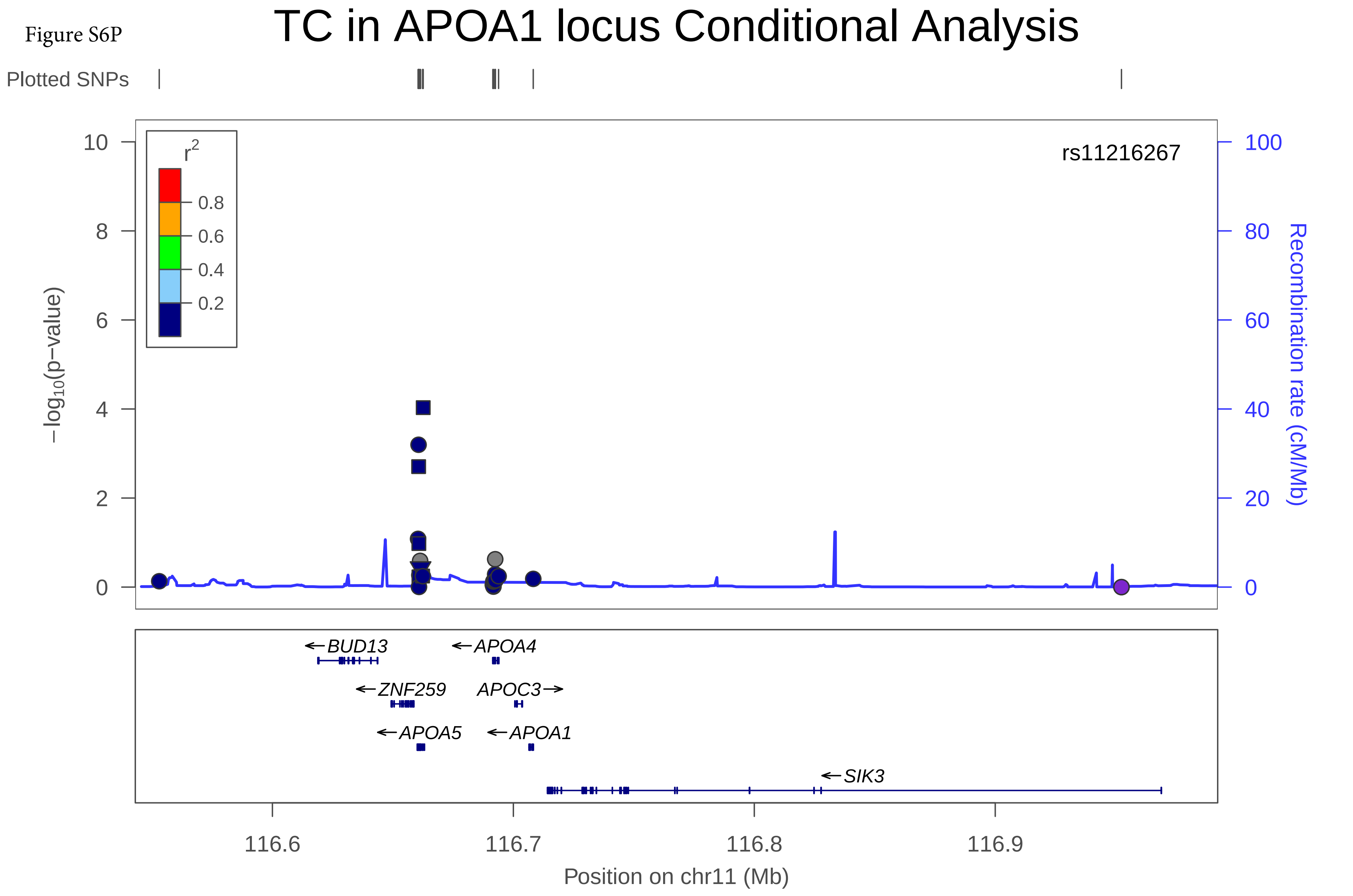
TC in APOA1 locus Conditional Analysis
Plotted SNPs
r2
0.8
0.6
0.4
0.2
rs11216267
−log10(p−value)
Recombination rate (cM/Mb)
10
8
6
4
2
0
100
80
60
40
20
0
BUD13
APOA4
ZNF259
APOC3
APOA5
APOA1
SIK3
116.6
116.7
116.8
116.9
Position on chr11 (Mb)

Figure S6Q

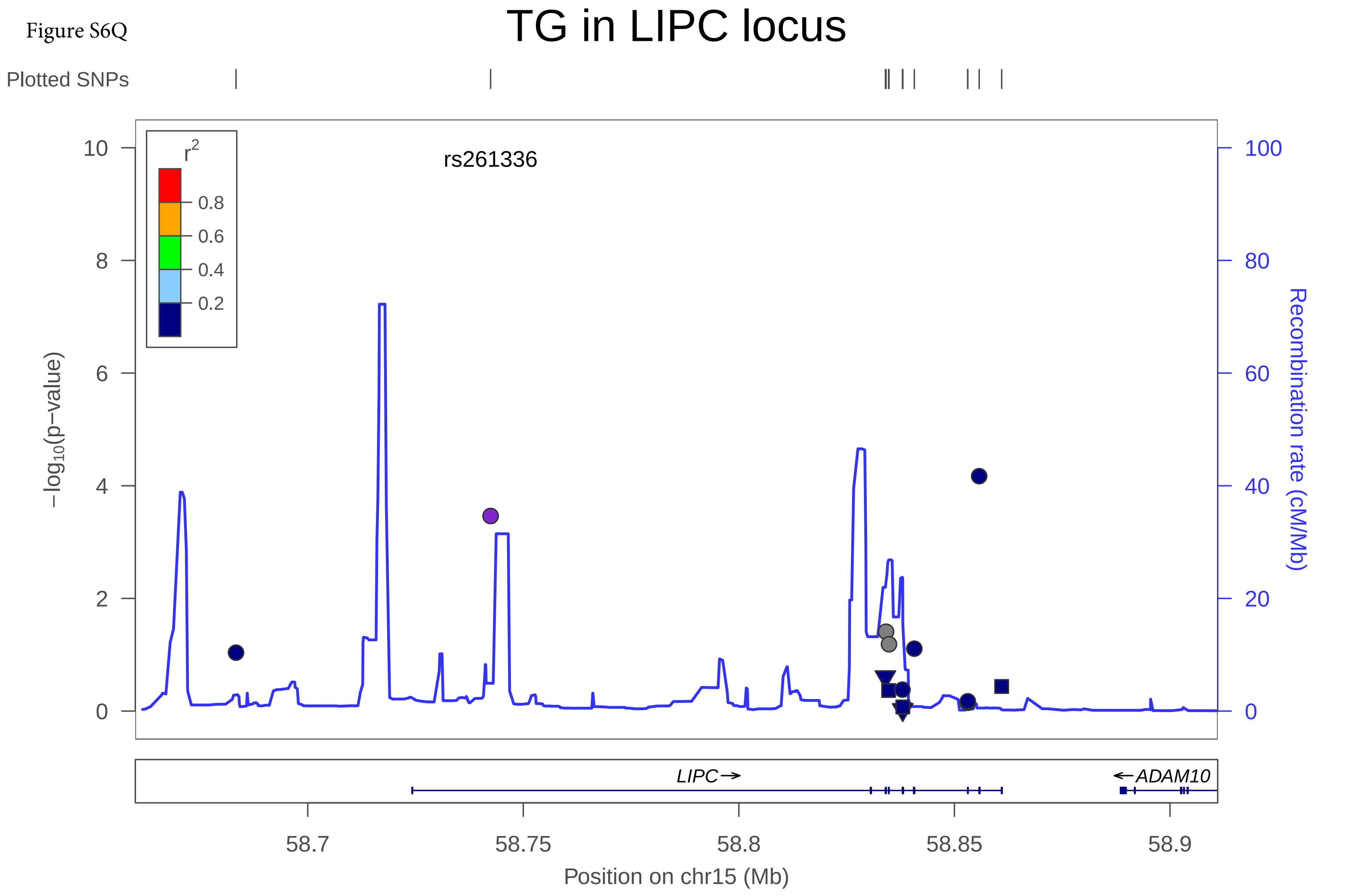
TG in LIPC locus
Plotted SNPs
r2
0.8
0.6
0.4
0.2
rs261336
−log10(p−value)
Recombination rate (cM/Mb)
10
8
6
4
2
0
100
80
60
40
20
0
LIPC→
←ADAM10
58.7
58.75
58.8
58.85
58.9
Position on chr15 (Mb)

Figure S6R

TG in LIPC locus Conditional Analysis

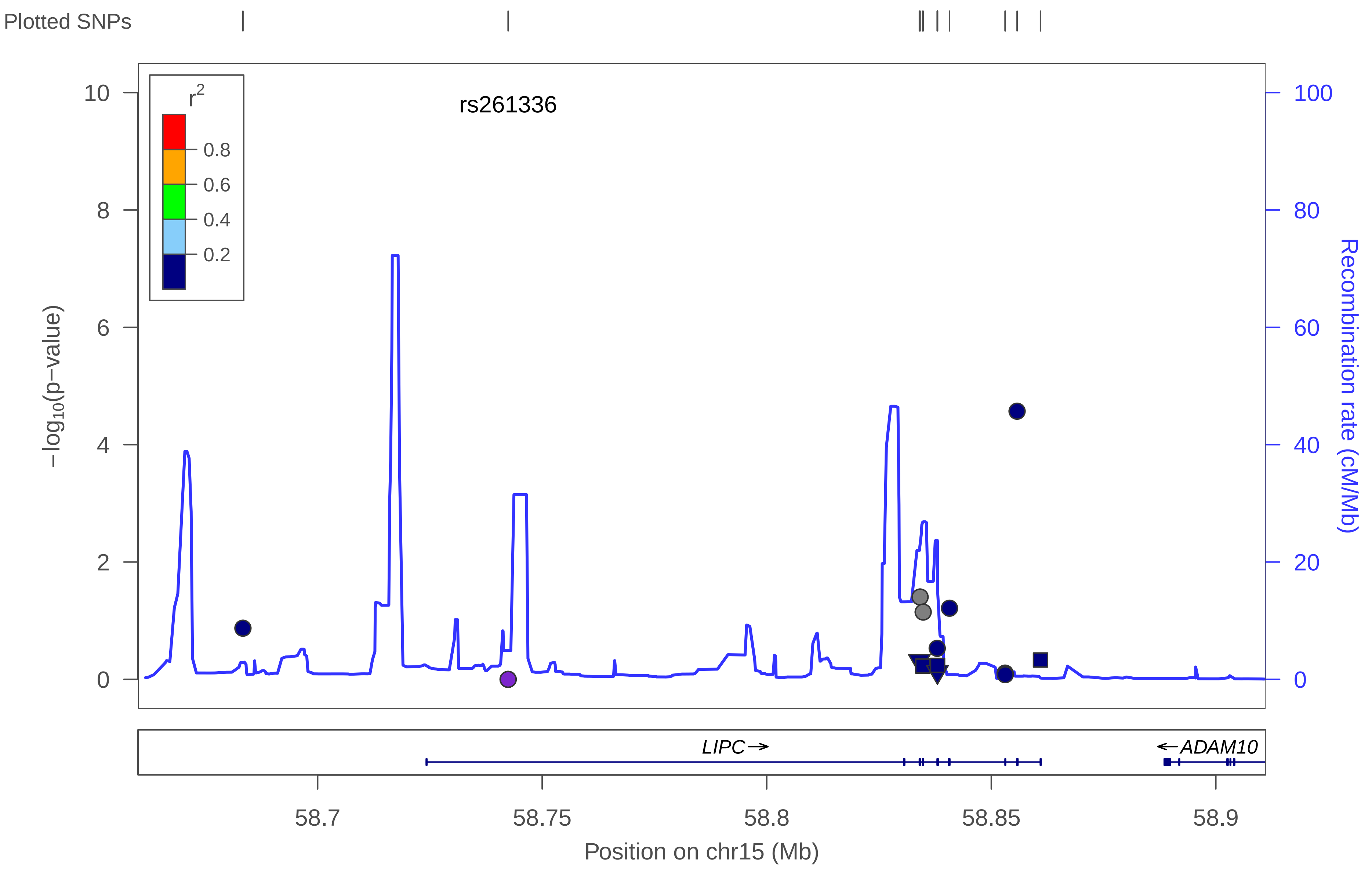

Figure S6S

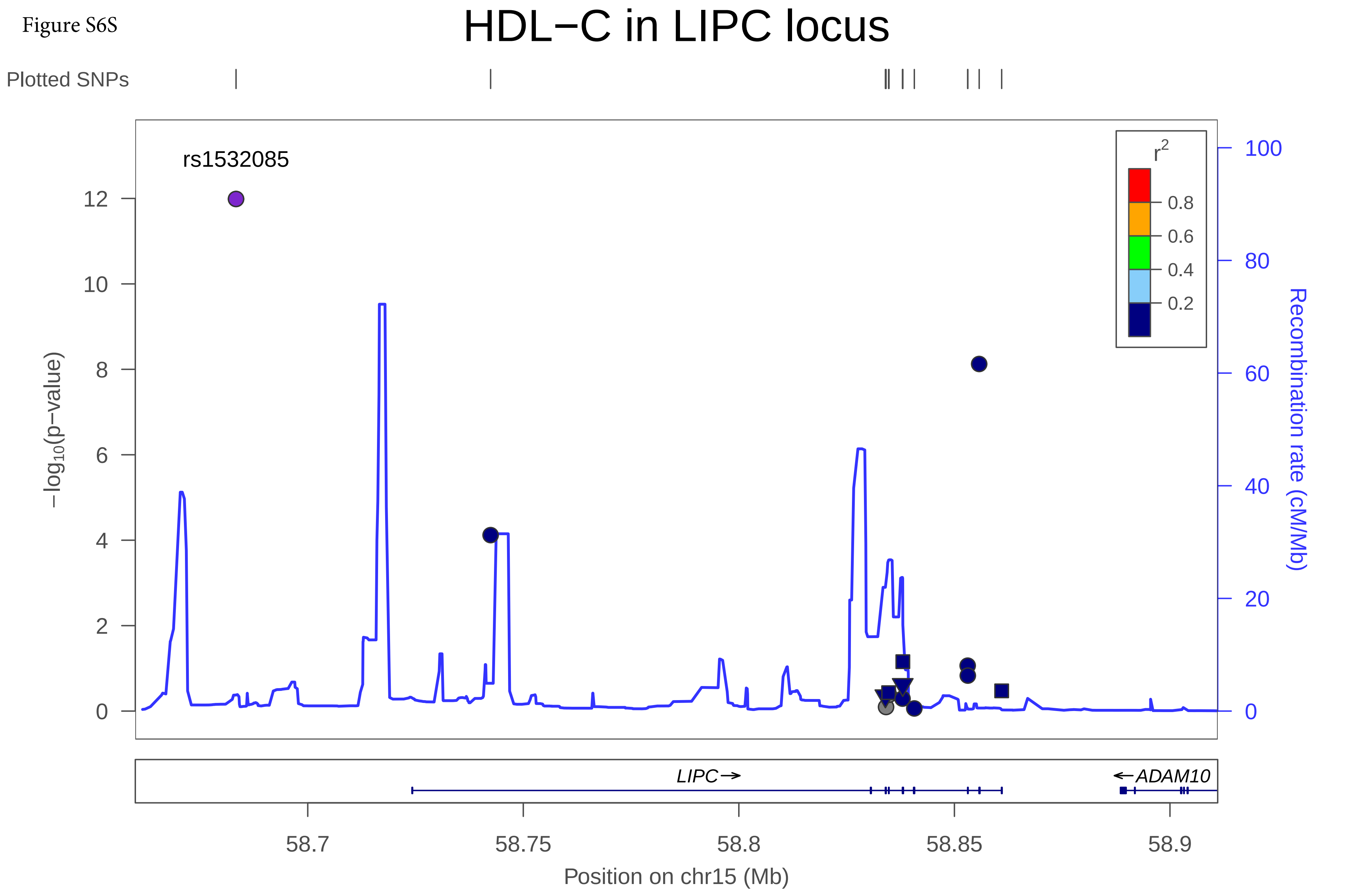
HDL−C in LIPC locus
Plotted SNPs
rs1532085
r2
0.8
0.6
0.4
0.2
−log10(p−value)
Recombination rate (cM/Mb)
12
10
8
6
4
2
0
100
80
60
40
20
0
LIPC→
←ADAM10
58.7
58.75
58.8
58.85
58.9
Position on chr15 (Mb)

Figure S6T

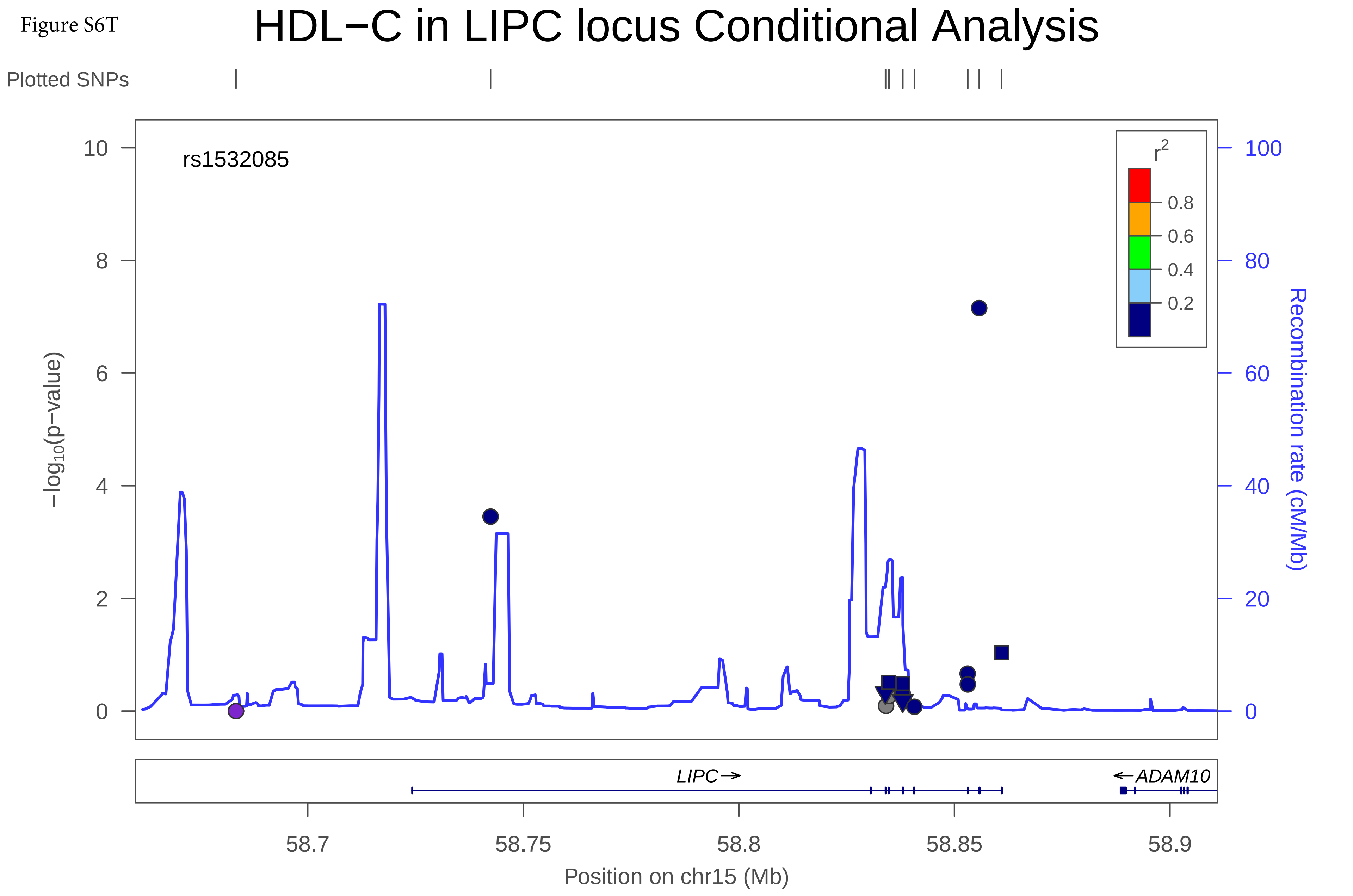

HDL−C in LIPC locus Conditional Analysis
Plotted SNPs
rs1532085
r²
0.8
0.6
0.4
0.2
−log10(p−value)
Recombination rate (cM/Mb)
0
2
4
6
8
10
0
20
40
60
80
100
LIPC→
←ADAM10
58.7
58.75
58.8
58.85
58.9
Position on chr15 (Mb)

Figure S6U

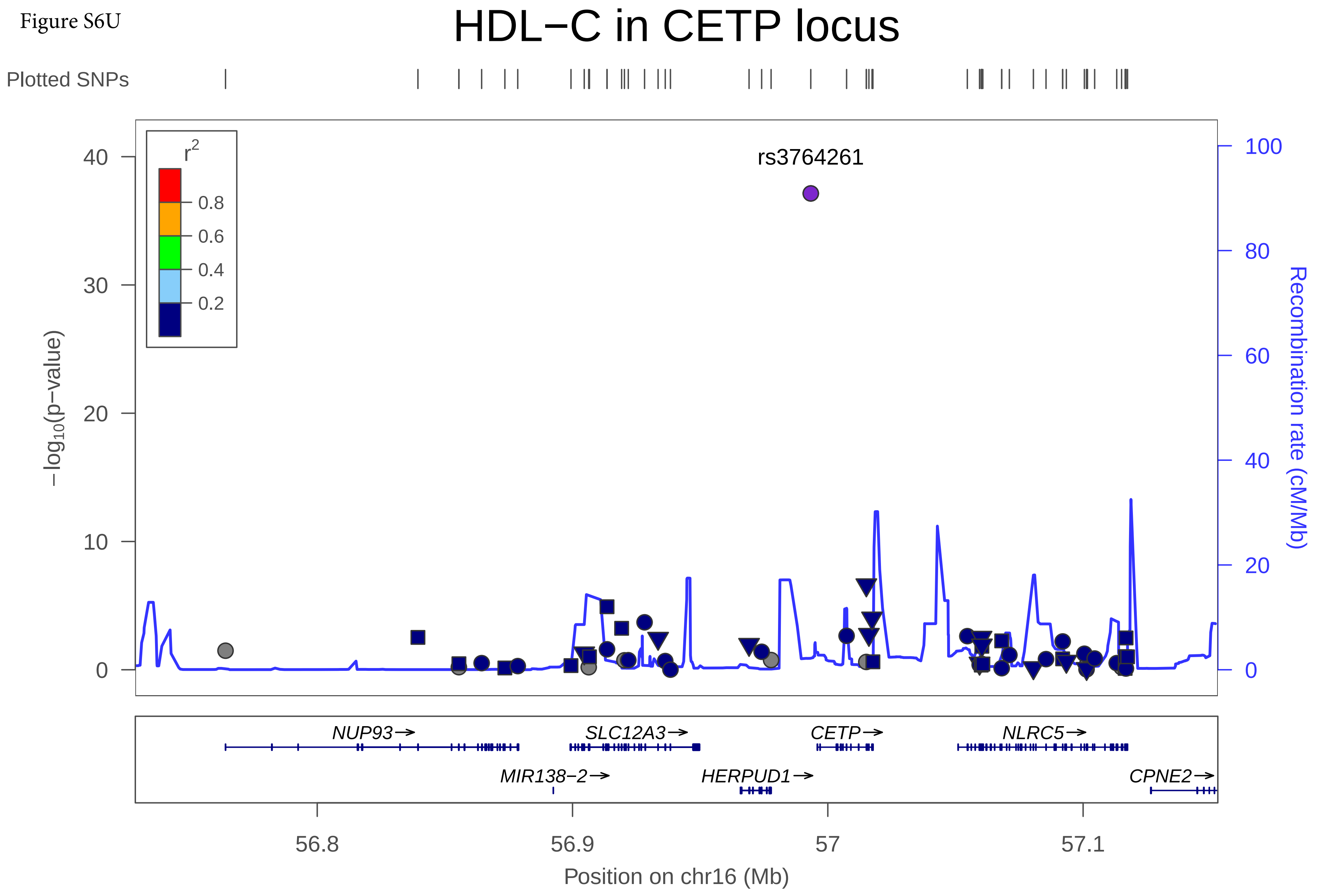
HDL−C in CETP locus
Plotted SNPs
rs3764261
r2
0.8
0.6
0.4
0.2
40
30
20
10
0
−log10(p−value)
100
80
60
40
20
0
Recombination rate (cM/Mb)
NUP93→
SLC12A3→
CETP→
NLRC5→
MIR138−2→
HERPUD1→
CPNE2→
56.8
56.9
57
57.1
Position on chr16 (Mb)

Figure S6V

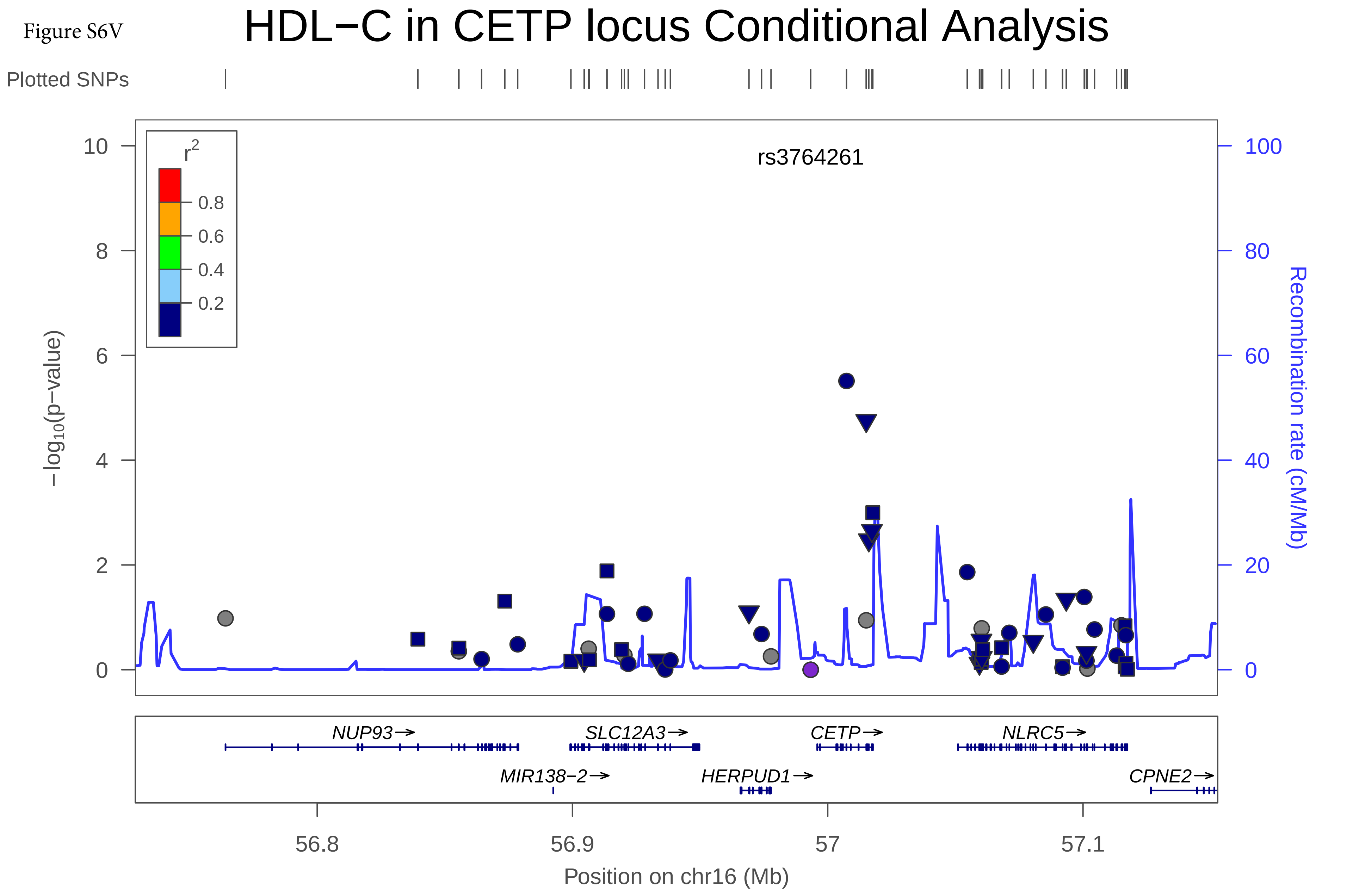
HDL−C in CETP locus Conditional Analysis
Plotted SNPs
rs3764261
r2
0.8
0.6
0.4
0.2
10
8
6
4
2
0
−log10(p−value)
100
80
60
40
20
0
Recombination rate (cM/Mb)
NUP93→
SLC12A3→
CETP→
NLRC5→
MIR138−2→
HERPUD1→
CPNE2→
56.8
56.9
57
57.1
Position on chr16 (Mb)
