# Supplementary material for: Re-sequencing Expands Our Understanding of the Phenotypic Impact of Variants at GWAS Loci
Source: PLoS Genet. 2014 Jan 30;10(1):e1004147. doi: 10.1371/journal.pgen.1004147 (PMC3907339; doi:10.1371/journal.pgen.1004147)
Supplement: Table S4 — Percent of variants in coding regions with MAF<1% in various studies. All comparisons involve persons of European ancestry. (DOCX) [file pgen.1004147.s010.docx]

Table S4. Percent of variants in coding regions with MAF<1% in various studies. All comparisons involve persons of European ancestry

| **Study^1^** | **%** |
| --- | --- |
| Present study - observed | 81% |
| Present study - downsampled to match Fu *et al*. | 79% |
| Present study - downsampled to match Tennessen *et al*. | 70% |
| Fu *et al*. | 93% |
| Tennessen *et al*. | 87% |
| Nelson *et al*. downsampled to present study | 95% |

^1^References: Nelson *et al*. (Science 2012; 337:100-104), Tennessen *et al*. (Science 2012; 337:64-69), Fu *et al*. (Nature 2013; 493:216-220)
