# Supplementary material for: Re-sequencing Expands Our Understanding of the Phenotypic Impact of Variants at GWAS Loci
Source: PLoS Genet. 2014 Jan 30;10(1):e1004147. doi: 10.1371/journal.pgen.1004147 (PMC3907339; doi:10.1371/journal.pgen.1004147)
Supplement: Text S1 — Supplementary methods. (DOC) [file pgen.1004147.s012.doc]

**Text S1**

***Samples and phenotypes***

The Northern Finland Birth Cohort of 1966 (NFBC) is a population birth cohort comprised of 12,058 individuals born in 1966 in the two northernmost provinces of Finland, Oulu and Lapland . Individuals have been followed longitudinally since birth; data for this study come from an assessment done at age 31 years where participants provided fasting blood samples for evaluation of the metabolic measures that are the focus of the current study , . At the same visit, height and weight were assessed for BMI, blood pressure was measured, and DNA was extracted for genotyping . Only individuals with data on one or more phenotypes were selected for sequencing.

The Finland-United States Investigation of NIDDM Genetics (FUSION) study , seeks to identify and characterize genes associated with type 2 diabetes (T2D) and related quantitative phenotypes, including body mass index, blood pressure, and lipid, glucose and insulin levels. To this end, we have ascertained >14,000 Finnish individuals, including >4,000 diabetics, with detailed phenotypic information; 1,838 consented samples (919 T2D cases, 919 controls) drawn from twelve birth provinces were selected for sequencing. Cases and controls were frequency-matched for birth province within Finland. Approximately 15% of cases and controls were born in Lapland or Oulu, the two provinces represented in the NFBC. Older controls were selected when available. No FUSION cases or controls were part of the NFBC sample.

All NFBC and FUSION participants included in this study provided informed consent. The studies are in accord with the approvals of the Ethical Committee of the Northern Ostrobothnia Hospital District (for NFBC), and the University of Michigan Health Sciences and Behavioral Sciences Institutional Review Board (IRB-HSBS) and the Institutional Review Board of the National Public Health Institute (KTL; now part of the National Institute for Health and Welfare, THL) (for FUSION).

The distribution of phenotypic variables overlaps considerably between NFBC and FUSION (Table S1), although means for all phenotypes were significantly different. These differences likely reflect the different age and sex distributions between NFBC and FUSION subjects: all NFBC study subjects were age 31 years at time of study, while FUSION subjects averaged 64 years (range = 41-91), and FUSION had a significantly higher proportion of males (55% vs 47%). Comparison of mean residual values after regressing on age, age^2^ and sex in the combined sample showed no significant differences between the cohorts for any phenotype. T2D cases were excluded from all analyses of fasting glucose and insulin.

***Selection of loci and genes for sequencing***

We focused on 17 loci (Table 1) shown to be significantly associated (p < 5x10^-8^) to lipid (HDL-C, LDL-C, and TG) and glycemic (insulin and glucose) phenotypes in previous studies . At each associated locus, we defined the interval of association as follows: we identified all SNPs with r^2^ ≥ 0.5 (in HapMap Phase II CEU samples) to the published phenotype-associated SNP and chose the most distant pair of SNPs from this set to identify an initial region of interest. We then extended the region of interest to the nearest recombination hotspots (≥10cM/Mb) on either side. Any gene partially or fully contained within the interval was considered a positional candidate. Each locus was visually inspected using LocusZoom .

We used several criteria to select a reduced set of loci for targeted resequencing. First, we used pre-existing genotype data to assess evidence of association within each association interval in the FUSION and NFBC cohorts. To maximize our likelihood of identifying disease-associated variants, we prioritized regions showing at least suggestive evidence of association in the combined cohorts (Table 1, sample size range over all phenotypes: 4,700 – 5,736); all but two loci had p<0.0001 to at least one previously associated phenotype in the NFBC cohort alone .

Next, we searched the literature to identify loci harboring genes believed to be causal on the basis of functional studies. We annotated genes known to play a role in monogenic diseases characterized by extreme lipid, insulin, or glucose levels (e.g. *LPL*; mutations cause familial combined hyperlipidemia and lipoprotein lipase deficiency , both associated with severe hypertriglyceridemia). We also targeted genes that affect phenotype levels when under or overexpressed in model organisms (e.g. *GALNT2*; knockdown in mouse results in elevated circulating HDL-C ). At some loci, multiple genes were identified as likely candidates (e.g. the *APO* cluster of genes on chromosome 11 associated with lipid levels). At loci with strong candidate genes, we restricted attention to those genes rather than all genes in the association interval defined above.

Finally, for each locus, we determined the number of probes required to sequence all genes of interest (either all genes in the interval, or all functional candidate genes; see details on probe design below). We selected a set of loci totaling ~2,000 probes, the limit of our capture reagent. Targeted genes are described in Table S2.

***Probe selection***

190-mer probes were designed on NCBI Build 36 to target all coding and untranslated exons of 78 target genes using all transcript isoforms available from Ensembl and Refseq databases. The probe sequences consist of 150bp of probe sequences overlapping target exon regions of the human reference, with custom sequences 20bp in length appended to the 3' and 5' ends (5' ACGGAAGGACCGGATCAAC, 3' CATTGCGTGAACCGAAGCT) to facilitate probe amplification and biotinylation. A total of 3,893 unique probes were designed, consisting of 2,206 primary probes and 1,687 secondary probes, which provide additional data for poorly performing primary probes.

***Whole genome amplification of genomic DNA***

The Qiagen REPLI-g service prepared 50ug of the whole genome amplified material. The starting material consisted of 250 ng of native DNA shipped to their services center for processing. Quantitation of the post amplification products was performed using a PicoGreen flourescence-based nucleic acid quantitation method and the products were checked also for quality using a stringent array based assay that checked the representation of various regions of the genomes.

***Sequencing methods***

Sequencing was performed at The Genome Institute at Washington University, St Louis (WUSTL). Illumina multiplexed libraries were constructed with 5µg of whole genome amplified material or 1µg native genomic DNA according to the manufacturer’s protocol (Illumina Inc, San Diego, CA) with the following modifications: 1) DNA was fragmented using a Covaris E220 DNA Sonicator (Covaris, Inc. Woburn, MA) to range in size between 100 and 400bp. 2) Illumina adapter-ligated library fragments were amplified in four 50µL PCR reactions for eighteen cycles. 3) Solid Phase Reversible Immobilization (SPRI) bead cleanup was used for enzymatic purification throughout the library process, as well as final library size selection targeting 300-500bp fragments. Samples for this project were multiplexed using Illumina barcoded libraries pooled together in pools of 12 or pools of 18 depending on the sequencing platform. A custom targeted probe set of 150bp probes was designed (Agilent Technologies, Santa Clara, CA) and captured ~270kb of primarily coding sequence from 78 genes. The concentration of each captured library pool was accurately determined through qPCR according to the manufacturer's protocol (Kapa Biosystems, Inc, Woburn, MA) to produce cluster counts appropriate for the Illumina GAIIx and HiSeq 2000 platform. The sample pools of twelve were loaded on the GAIIx and pools of eighteen were loaded on the HiSeq using paired end 101bp read lengths, with a coverage metric aim of 80% of the targeted space to be covered at a depth of 20x or higher. Individual identity was confirmed by comparing sequence data with pre-existing genotype array data.

The mean depth of coverage (per bp per person) for the 78 targeted genes ranged from 31-285x (Table S2, Figure S2). For sixty-eight genes, >80% of targeted basepairs had ≥10x coverage in >80% of subjects. Over 96% of 272,043 targeted bases had genotype quality score >50 in >75% of individuals (range of 60-100%, Figure S2, Table S2). Poor coverage in a gene mainly reflected GC content; genes with mean GC >60% averaged 91% of bases with genotype quality score >50 in >75% of individuals, while genes with mean GC <60% averaged 97% with such a score (Figure S2).

***Production of variant calls***

Each of the three centers (UCLA, WUSTL, and UM) independently generated its own call sets and compared results to produce a consensus variant set.

*Generation of the UCLA call set*

Variant calling and filtration were performed in several steps:

1. Variant discovery and filtration in individual samples Single nucleotide variants and short indels were called in each sample independently using SAMtools 0.1.18 with the following parameters: min. mapping quality 30, min. base quality 15, BAQ enabled; minimum read depth 3. Minimum allowed variant quality (QUAL) was 50; sites with more than one alternative allele in the same sample or within 10 bp of a gap were skipped.

2. Variant filtration based on all samples Variants were accepted if they were observed in more than one sample. Those observed in one sample only (singletons) were accepted if their quality was above 100 (=2*50) and they were heterozygous.

3. Genotype calling and the last round of filtration At this step, SAMtools was used to call genotypes in each sample at each segregating site detected above. Single-sample VCF files were merged together. In the resulting VCF file, individual genotypes were set to missing if their variant quality or genotype quality (GQ) were below 50. A segregating site was filtered out completely if it contained missing genotypes in more than 25% of samples or if all high-quality genotypes were homozygous reference. This conservative procedure finally generated 2,543 variants in 6,123 samples.

*Generation of the WUSTL call set*

Data were aligned with BWA v0.5.9 with quality trimming (-q 5) to remove low quality bases at the ends of reads to the GRCh37-lite reference sequence. Data from individual runs were merged, if necessary, with Picard (http://picard.sourceforge.net) v1.29. All reads were deduplicated using Picard MarkDuplicates.

Single nucleotide variants were called using SAMtools v1.16 (samtools pileup –cv) with default settings and VarScan 2.2.9 . SAMtools calls were retained if they met all of the following rules inspired by MAQ :

1. Site is greater than 10bp from a predicted indel of quality 50 or greater
2. The maximum mapping quality at the site is ≥ 40
3. Fewer than 3 SNV calls in a 10 bp window around the site
4. Site is covered by at least 3 reads and less than 1,000,000,000 reads
5. Consensus quality ≥ 20
6. SNP quality ≥ 20.

VarScan was run with the following parameters:

1. Minimum coverage of 3 reads
2. Minimum variant base frequency of 20%
3. Maximum p-value of 0.1
4. Strand-filter on
5. Minimum mapping quality of 10.

Variant calls from VarScan and SAMtools (after filtering) were unioned using joinx v1.3 (https://github.com/genome/joinx). Conflicts where both callers predicted a variant at a position, but with differing genotypes were resolved by taking the SAMtools prediction. The resulting merged set of variants were additionally filtered to remove likely false positives (, ). We used bam-readcount v0.4 (https://github.com/genome/bam-readcount) with a minimum base quality of 15 (-b 15) to generate metrics and marked sites as filtered based on the following requirements:

1. Minimum variant base frequency at the site of 5%
2. Percent of reads supporting the variant on the plus strand ≥ 1% and ≤ 99% (variants failing these criteria are filtered only if the reads supporting the reference do not show a similar bias)
3. Minimum variant base count of 4
4. Variant falls within the middle 90% of the aligned portion of the read
5. Maximum difference between the quality sum of mismatching bases in reads supporting the variant and reads supporting the reference of 100
6. Maximum mapping quality difference between reads supporting the variant and reads supporting the reference of 30
7. Maximum difference in aligned read length between reads supporting the variant base and reads supporting the reference base of 25
8. Minimum average distance to the effective 3’ end of the read for variant supporting reads of 20% of the sequenced read length
9. Maximum length of a flanking homopolymer run of the variant base of 5.

Once all variant sites in all individuals were predicted, sites were limited to the precise target space of the capture product and aggregated into a list of segregating sites for the cohort. Then, for each segregating site, we genotyped non-variant individuals using SAMtools (samtools pileup –c), pre-limited to the regions surrounding each segregating site (using SAMtools view –u –L). The resulting genotypes were added to sites missing from the original VCF for each sample to distinguish between missing data and homozygous reference calls. The resulting single-sample VCF files, containing genotypes for all segregating sites, were subsequently merged using joinx. Segregating sites were filtered out if more than 50% of the samples with variant calls had failed the single sample filtering (as determined using joinx vcf-site-filter –min-fail-filter 0.5). This process generated a list of 3,307 segregating sites across 6,123 samples.

*Generation of the UM call set*

We took a two-step approach for discovering variants and genotyping candidate sites. First, genotype likelihood files (GLFs) were generated using SAMtools pileup on individual BAM files. Next, we used glfMultiples - a multi-sample variant caller - to generate initial SNV calls. This process allowed us to perform multisample calling of variants simultaneously across thousands of samples to avoid batch effects. Details of the likelihood model implemented in glfMultiples are given in Li 2008 . Briefly, the distribution of observed bases and quality scores at each location (conditional on the true genotype) is modeled using the MAQ model . Using maximum likelihood, we then estimated an allele frequency for each site under the assumption that genotypes were segregating in Hardy-Weinberg proportions. Genotypes for each site and corresponding posterior probabilities were then calculated via Bayes theorem, using the Hardy-Weinberg proportions as priors and the MAQ error model to describe the conditional probability of bases and quality scores given the true genotype.

After these initial SNV calls were generated, we re-examined the BAM files to collect additional information about each variant site. Filters considered the total read depth, the number of individuals with coverage at the site, the fraction of variant reads in each heterozygote, the ratio of forward and reverse strand reads for reads carrying reference and variant alleles and the average position of variant alleles along a read.

The final SNV call set included variants that were called with posterior probability >99% (glfMultiples SNP quality >20), were >5bp away from an indel detected in the 1000 Genomes Pilot Project (37), and were covered by at least one read in 85% of samples. Sites having >65% of reads as heterozygotes carrying the variant allele or where the absolute squared correlation between allele (variant or reference) and strand (forward or reverse) was >0.15 were excluded. All processing steps were automated using GNU make, which facilitates parallelization of data processing steps, dependency checking, and repeats of failed runs (Jun et al. in prep, [http://genome.sph.umich.edu/wiki/UMAKE](https://mail.mednet.ucla.edu/owa/redir.aspx?C=iAVwo2F0UEqkJaMEt0B0DN6YNT-iVtAIjtK_jEvKQAWslcbFdZgHxlB2s1qUy0yKVVQ3OHb4wl0.&URL=http%3A%2F%2Fgenome.sph.umich.edu%2Fwiki%2FUMAKE)).

*Generation of consensus set*

To generate a consensus data set we first pooled together all quality controlled sites discovered by any center in the defined target regions (n=2,306). We excluded from this list non bi-allelic sites or sites with different alternative allele (n=72). Next, each center re-called SNP genotypes at all sites (n=2,234). Finally, a majority vote approach (2 out of 3) was used to generate the consensus variant data set. In brief, genotypes concordant between at least two centers were propagated into the consensus data set; otherwise, they were set to missing. The overall concordance rate between centers was 99.96% (99.99%, 99.94% and 99.95% for homozygous reference, heterozygous and homozygous alternative genotypes, respectively).

Among the 2,234 sites that passed our consensus procedure for variant calling 13 were monomorphic for the non-reference alternate allele. Among all 2,221 polymorphic variants, 1,697 (76%) were rare (MAF<0.5%) and 1,072 (48%) were observed in only one or two copies. Variant annotation and effect prediction were performed with MapSNPs/PolyPhen2 ver. 2.2.2 using dbSNP v.135 and knownCanonical UCSC gene set .

***Validation***

All genotypes at sites seen in three or fewer copies that were not in dbSNP 135 were candidates for validation (1,104 sites, 1,484 genotypes). To validate these genotypes, we performed two rounds of validation using Roche/454 sequencing. For both rounds, analysis of the Roche/454 data was performed in three sequential steps: (1) alignment of reads to the reference sequence, (2) assignment of mapped reads to the appropriate amplicon, and (3) consensus calling and validation of predicted variants on a per-amplicon basis. All reads from each 454 pool were aligned to the GRCh37-lite (hg19) reference sequence using the *bwasw* algorithm of the *bwa* aligner v.6.1-r104 and converted to BAM format.

*Analysis of Round 1*

Of the 1,104 amplicons designed for the first validation experiment, 765 partially overlapped another amplicon by at least one base. To ensure accuracy in the validation results, 454 reads were assigned to individual amplicons prior to consensus variant calling. First, all overlapping amplicon relationships were defined as one of four possibilities:

1. Two amplicons overlap but have different start and end positions (602 pairs)
2. One amplicon is completely contained within the other (155 pairs)
3. Two amplicons share an end position but not start position (49 pairs), or
4. Two amplicons share a start position but not end position (46 pairs)

To be considered for assignment to an amplicon, we required that a read’s alignment overlap at least 50% of amplicon bases, and start or stop within 10 base pairs of the amplicon’s outer coordinates. In the case that a read met these criteria for two or more overlapping amplicons, the amplicon whose start position was closest to the read alignment start position was selected. If the overlapping amplicons had the same start position, the one with end position was closest to the read end position was selected. The number of bases overlapping between read sequence and amplicon sequence served as a tie-breaker. Reads that could not be unambiguously assigned to an amplicon were discarded.

Following read-to-amplicon assignment, the validation proceeded on a per-amplicon basis as follows. First, the alignments of reads assigned to the amplicon were converted to BAM format. Next, the position of the targeted variant was retrieved via SAMtools pileup and called using VarScan 2 v2.2.11. Candidate SNVs were classified as "no coverage" (n) if there were < 30 total reads covering that site. In each of the four pools, sufficiently covered variants were considered validated if the validation call matched the prediction, or refuted if the validation call matched the reference. Variants that were observed in WGA DNA but refuted in genomic DNA were classified as WGA-only artifacts. Variants whose validation call matched neither reference nor variant were classified as “other”. In all cases, these indicated that the true genotype was homozygous.

*Analysis of Round 2*

For the second validation experiment, we separated out amplicons across 8 separate pools to prevent overlap and ensure that samples could be unambiguously assigned. Despite this we had 2 amplicons that overlapped another within 2 different pools. Both overlapping pairs had different start and end positions. Reads were assigned to all amplicons identically to as described for the first round of validation.

We included an amplicon in this experiment based on its results from the first validation experiment. Amplicons included were:

1. 523 amplicons where no coverage was obtained
2. 20 positive controls where the first experiment validated the site
3. 94 amplicons covering sites refuted from the first experiment
4. 74 amplicons covering sites refuted in the first experiment amplified from WGA source material

In all cases, we used genomic DNA as the source material where it was available unless otherwise specified. The assignment of validation status for the second round of validation was identical to that of the first.

*Assignment of final validation status*

To assign a single validation status to each variant we preferentially chose the validation status from a single round, based on the following hierarchy:

1. genomic DNA from round 2
2. genomic DNA from round 1
3. WGA DNA from round 2
4. WGA DNA from round 1.

Overall, 92% of genotypes were validated and an additional 11 singleton sites were validated, but found to be homozygous rather than heterozygous for the alternative allele. To be conservative, sites that were validated only in WGA DNA (336 sites) were excluded from further analysis. After validation experiments were complete, dbSNP 137 was released. We cross-referenced all 441 non-validated sites with dbSNP 137, and saw that 76 sites were in dbSNP 137. Four sites were clearly refuted in our validation experiments, the remaining 72 sites were either not covered well in validation procedures (five sites) or validated only in WGA (67 sites). We re-included these 72 sites to have a total of 1,852 variants for final analysis.

***Weighted Burden Test Analysis Methods***

We used a weighted sum score based linear model to test the collective effect of multiple rare variants within a gene.

$$y=\alpha+\beta\left(\sum_{i=1}^{m} w_{i}g_{i}\right)+\sum_{j=1}^{k} \gamma_{j}x_{j}+\varepsilon$$

*y* is the observed quantitative phenotype, *α* the grand mean (or intercept), *m* the number of selected rare variants in a gene, *g*_i_ the copy number (0, 1 or 2) of minor allele of variant *i, w*_i_ the weight of variant *i*. The term Σ*w*_i_*g*_i_ in the model is the weighted sum score of multiple variants and *β* is the collective effect coefficient for the score. *k* is the number of covariates, *x*_j_ the *j*-th covariate*, γ*_j_ the effect of the *j*-th covariate, *ε* the residual effect. In the CMC method , *w_i_* is set to 1 for all rare variants and the sum score Σ*w*_i_*g*_i_ is limited to 1 if it is larger than 1. Analyses used the first 5 PCs as covariates.

***References***

1. Rantakallio P (1969) Groups at risk in low birth weight infants and perinatal mortality. Acta Paediatr Scand 193: Suppl 193:191+.

2. Pouta A, Hartikainen AL, Sovio U, Gissler M, Laitinen J, et al. (2004) Manifestations of metabolic syndrome after hypertensive pregnancy. Hypertension 43: 825-831.

3. Taponen S, Martikainen H, Jarvelin MR, Sovio U, Laitinen J, et al. (2004) Metabolic cardiovascular disease risk factors in women with self-reported symptoms of oligomenorrhea and/or hirsutism: Northern Finland Birth Cohort 1966 Study. J Clin Endocrinol Metab 89: 2114-2118.

4. Bennett A, Sovio U, Ruokonen A, Martikainen H, Pouta A, et al. (2005) No association between insulin gene variation and adult metabolic phenotypes in a large Finnish birth cohort. Diabetologia 48: 886-891.

5. Valle T, Tuomilehto J, Bergman RN, Ghosh S, Hauser ER, et al. (1998) Mapping genes for NIDDM. Design of the Finland-United States Investigation of NIDDM Genetics (FUSION) Study. Diabetes Care 21: 949-958.

6. Silander K, Scott LJ, Valle TT, Mohlke KL, Stringham HM, et al. (2004) A large set of Finnish affected sibling pair families with type 2 diabetes suggests susceptibility loci on chromosomes 6, 11, and 14. Diabetes 53: 821-829.

7. Kathiresan S, Melander O, Guiducci C, Surti A, Burtt NP, et al. (2008) Six new loci associated with blood low-density lipoprotein cholesterol, high-density lipoprotein cholesterol or triglycerides in humans. Nat Genet 40: 189-197.

8. Willer CJ, Sanna S, Jackson AU, Scuteri A, Bonnycastle LL, et al. (2008) Newly identified loci that influence lipid concentrations and risk of coronary artery disease. Nat Genet 40: 161-169.

9. Dupuis J, Langenberg C, Prokopenko I, Saxena R, Soranzo N, et al. (2010) New genetic loci implicated in fasting glucose homeostasis and their impact on type 2 diabetes risk. Nat Genet 42: 105-116.

10. Teslovich TM, Musunuru K, Smith AV, Edmondson AC, Stylianou IM, et al. (2010) Biological, clinical and population relevance of 95 loci for blood lipids. Nature 466: 707-713.

11. Sabatti C, Service SK, Hartikainen AL, Pouta A, Ripatti S, et al. (2009) Genome-wide association analysis of metabolic traits in a birth cohort from a founder population. Nat Genet 41: 35-46.

12. Pruim RJ, Welch RP, Sanna S, Teslovich TM, Chines PS, et al. (2010) LocusZoom: regional visualization of genome-wide association scan results. Bioinformatics 26: 2336-2337.

13. Babirak SP, Iverius PH, Fujimoto WY, Brunzell JD (1989) Detection and characterization of the heterozygote state for lipoprotein lipase deficiency. Arteriosclerosis 9: 326-334.

14. Havel RJ, Gordon RS, Jr. (1960) Idiopathic hyperlipemia: metabolic studies in an affected family. J Clin Invest 39: 1777-1790.

15. Li H, Durbin R (2009) Fast and accurate short read alignment with Burrows-Wheeler transform. Bioinformatics 25: 1754-1760.

16. Koboldt DC, Zhang Q, Larson DE, Shen D, McLellan MD, et al. (2012) VarScan 2: somatic mutation and copy number alteration discovery in cancer by exome sequencing. Genome Res 22: 568-576.

17. Li H, Ruan J, Durbin R (2008) Mapping short DNA sequencing reads and calling variants using mapping quality scores. Genome Res 18: 1851-1858.

18. Larson DE, Harris CC, Chen K, Koboldt DC, Abbott TE, et al. (2012) SomaticSniper: identification of somatic point mutations in whole genome sequencing data. Bioinformatics 28: 311-317.

19. Genomes Project C, Abecasis GR, Altshuler D, Auton A, Brooks LD, et al. (2010) A map of human genome variation from population-scale sequencing. Nature 467: 1061-1073.

20. Adzhubei IA, Schmidt S, Peshkin L, Ramensky VE, Gerasimova A, et al. (2010) A method and server for predicting damaging missense mutations. Nat Methods 7: 248-249.

21. Sherry ST, Ward MH, Kholodov M, Baker J, Phan L, et al. (2001) dbSNP: the NCBI database of genetic variation. Nucleic Acids Res 29: 308-311.

22. Hsu F, Kent WJ, Clawson H, Kuhn RM, Diekhans M, et al. (2006) The UCSC Known Genes. Bioinformatics 22: 1036-1046.

23. Li B, Leal SM (2008) Methods for detecting associations with rare variants for common diseases: application to analysis of sequence data. Am J Hum Genet 83: 311-321.
